# Supplementary material for: The Trajectory Statistics of Biological Exploratory Dynamics
Source: bioRxiv. 2026 Sep 17:2026.09.16.750450. Preprint. [Version 1] doi: 10.64898/2026.09.16.750450 (PMC13596309; doi:10.64898/2026.09.16.750450)
Supplement: Supplementary file 1 [file NIHPP2026.09.16.750450v1-supplement-1.pdf]

bioRxiv preprint doi: https://doi.org/10.64898/2026.09.16.750450; this version posted September 17, 2026. The copyright holder for this preprint (which was not certified by peer review) is the author/funder, who has granted bioRxiv a license to display the preprint in perpetuity. It is made available under a CC-BY 4.0 International license.

Supplemental Material for *The Trajectory Statistics of Biological Exploratory Dynamics*

A Quixotic exploration through random walks . . . 2
1 Impact of confinement and finite system sizes . . . 9
2 Conclusions about bare random-walks . . . 12
3 Helpfulness of resets in random walks of higher dimensionality . . . 12
B First-passage properties of ordinary random walks . . . 12
1 First passage times by lattice counts . . . 14
2 Interpretations and continuum approximations . . . 19
3 Survival probability . . . 19
C Analytics of random walks with deterministic reset . . . 19
1 Probability distribution of random searcher at position x at time t . . . 20
2 Survival probability of random searcher. . . . 21
3 Mean first passage time of random searcher . . . 21
D Average hitting times as explicit averages over trajectories . . . 23
E Resets confine the search of the random walker . . . 24
1 Resets place an upper bound on the spread of the distribution . . . 24
2 Equivalence of asymptotics of mean first passage times . . . 25
F Additional results for random walks with resetting . . . 27
1 Additional sweep of behavior of random walk under resets from simulation . . . 27
2 The geometric distribution as a language of exploration with memoryless resets . . . 28
G Dependence of the mean first-passage time on the reset rate r in 1D with diffusion and drift . . . 30
H Random walk with reset and exponential cue . . . 32
1 Nondimensionalization of the exponential-cue backward equation . . . 32
2 Solving the backward equation for the mean first-passage time . . . 33
3 Asymptotic checks for the exponential cue solution . . . 36
a Large Pe for fixed $\tilde{\ell}$ and $\tilde{T}_R$ . . . 36
b Large $\tilde{T}_R$ for fixed $\tilde{\ell}$ and Pe . . . 38
4 Existence of a minimum for the optimal reset time . . . 41
I The Near-Target Limit for a Single Checkpoint . . . 44
J Parallel search with multiple independent searchers . . . 44
1 Mean earliest-arrival time from the collective survival probability . . . 44
2 Derivation of the backward equation for the survival probability . . . 45
3 Characteristic earliest-arrival time and relation to the mean . . . 46
4 Short-time and large-N asymptotics of the earliest-arrival time . . . 48
5 Approach to the reset-independent large-N regime . . . 51
6 Optimal number of searchers in the presence of a resource cost . . . 52
K Additional results for parallel search . . . 53
1 Large-N behavior of the Mth arrival . . . 53
2 Approach to the reset-independent regime for the Mth arrival . . . 54
3 Long-time survival and the last arrival . . . 55
L Derivation of the first-passage Brownian bridge statistics . . . 56
1 Conditional probability density . . . 56
2 Mean and variance . . . 57
3 Monotonicity and maximal excursion . . . 57
M First-passage Brownian bridges with stochastic resetting . . . 59
1 Conditional probability density and local transition kernel . . . 59
2 Decomposition into failed and successful excursions . . . 61
3 Mean temporal organization of reset-conditioned trajectories . . . 62
4 Mean number of resets in the conditioned first-passage ensemble . . . 63

bioRxiv preprint doi: https://doi.org/10.64898/2026.09.16.750450; this version posted September 17, 2026. The copyright holder for this preprint (which was not certified by peer review) is the author/funder, who has granted bioRxiv a license to display the preprint in perpetuity. It is made available under a CC-BY 4.0 International license.

A: Quixotic exploration through random walks

In principle, organisms, cells, and molecules *could* navigate spaces in pursuit of target states by the simplest algorithm possible: at each moment in time, flip an independent coin that decides where to step next, assembling an unguided random walk that diffuses searchers throughout their physical or behavioral spaces. Indeed, many chemical reactions in cells proceed dependably enough by diffusive, mass-action encounters in the crowded compartments of cells or organisms.

However, for some search processes, this type of unstructured diffusive motion is often *not* the means by which biological systems explore or sample physiological variation. Instead, as prospectively hinted at in Fig. 2 and Fig. 3 of the main text (§III), many examples of exploratory dynamics are decorated by additional mechanisms (such as the surprising presence of "resets" aborting even imminently successful trajectories; rescues from these resets via checkpoints; parallelism; directional drift; signaling and sensing via cues; and beyond). Why? What makes these modifications to the most primordial search algorithm necessary or useful, given that these modifications often cost energy or impose architectural complexity?

dimension	probability of ever hitting target	mean time to hit target
$d = 1$	$p(\text{hit target}) = 1$	$\langle T \rangle = \infty$
$d = 2$	$p(\text{hit target}) = 1$	$\langle T \rangle = \infty$
$d \geq 3$	$p(\text{hit target}) < 1$	$\langle T \rangle = \infty$

TABLE S1: Properties of ordinary random walks. See (for instance) Refs.[16,17,21].

In short, one hypothesis is that such ordinary random walks can be too slow to rely on to find targets, or even fail to do so completely. One explicit biological example where unmodified random walk searches might have biologically forbidding slow speeds, or failure rates, is the concrete setting of the capture of chromosomes by microtubules in cell division[12]. Specifically, the centrosome must capture all chromosomes within a time acceptable to the pace of mitosis. Since the dynamic instability of microtubules underlying this process requires constant injection of biochemical energy, the time to accomplish these searches has biological and bioenergetic implications.

To appreciate the stakes (and motivate our journey through various mechanisms in the escapades of this paper), we now tour briskly some mathematical facts to this effect. First, consider the typical time that trajectories take to reach a target. As summarized by the third column of Table S1, even in one or two dimensions, the distribution of first-passage times is sufficiently broad that the mean time to hit a target $\langle T \rangle$ is *infinite*, at least in an unbounded region.

This latter chronological catastrophe may be appreciated from many angles; one is to directly calculate the probability of a walker arriving at a target in one dimension by exact lattice counts, as discussed explicitly and pedagogically in Appendix B. The resulting probability $f(t)dt$ of first arriving at a target near time t can be described in a continuum approximation as scaling with $f(t) \sim t^{-3/2}$ in one dimension (see the Appendix, eqn. S32), anticipating a divergent mean first arrival time to any target.

Next, consider the probability of ever arriving at a particular target location in spaces of increasing dimension, as summarized by the second column of Table S1. In one or two dimensions, an ordinary random walk will always eventually arrive at a target at any finite separation from a starting point. However, in spaces of three or more dimensions, the situation is spectacularly worse. Trajectories of such walks may never reach a specific target's location at all (namely, they show a hitting or recurrence probability of less than one)!

This remarkable dimensionality-dependent behavior motivates the memorable quip, "A drunk man will eventually find his way home, but a drunk bird may get lost forever," as limned by Shizuo Kakutani[2,9]. (Here inebriation is a proxy for genuinely random wandering in either two or three dimensions.) Pòlya proved this subtle and counterintuitive fact in a celebrated recurrence theorem[21,24]. Amusingly (relatably?), Pòlya was originally motivated to consider the mathematical problem by force of social embarrassment. Going for a (two-dimensional) walk in his town of Zurich, he happened to encounter a student with their lover, then—his chagrin growing—met them again and again over the same walk. Pòlya wondered whether this property of two random walks meeting could be expected to be generic[22] ("Two Incidents" p. 582–585); see also Refs.[15,18,21]. First, he realized that the question of whether two random walks could be expected to meet eventually is equivalent to the question of whether a single walk starting at the origin can be expected to return there. To appreciate this equivalence, take two walks that *do* encounter each other at some point; reverse the steps of one of the walks; and append it to the other walk at their meeting place. The new longer walk definitely returns to the origin, as illustrated by Fig. S1. This operation maps every pair of walks that encounter each other to some other walk that accomplishes a return to the origin[6]. Empowered to focus on this question of recurrence, classic and modern sources[16,17] (including Pòlya's original arguments[21]) typically establish the dimensional breakdown in the certainty of visiting a site using heavy technical formalisms such as the machinery of generating functions, or else ingenious, but intricate, analogies to electrical circuits[8].

Instead, we now briefly trace a heuristic argument, avoiding technical fussiness to display the fundamental essence, for why the probability of ever visiting some site located at a finite distance $\mathbf{x}_T$ (a distance R away) is less than one for spaces of dimensionalities three or larger. We will argue this as follows. First, we recognize that the desired probability of ever visiting a site is at most the marginal probability that it visits the site at a particular time t, summed over all possible times from zero to

bioRxiv preprint doi: https://doi.org/10.64898/2026.09.16.750450; this version posted September 17, 2026. The copyright holder for this preprint (which was not certified by peer review) is the author/funder, who has granted bioRxiv a license to display the preprint in perpetuity. It is made available under a CC-BY 4.0 International license.

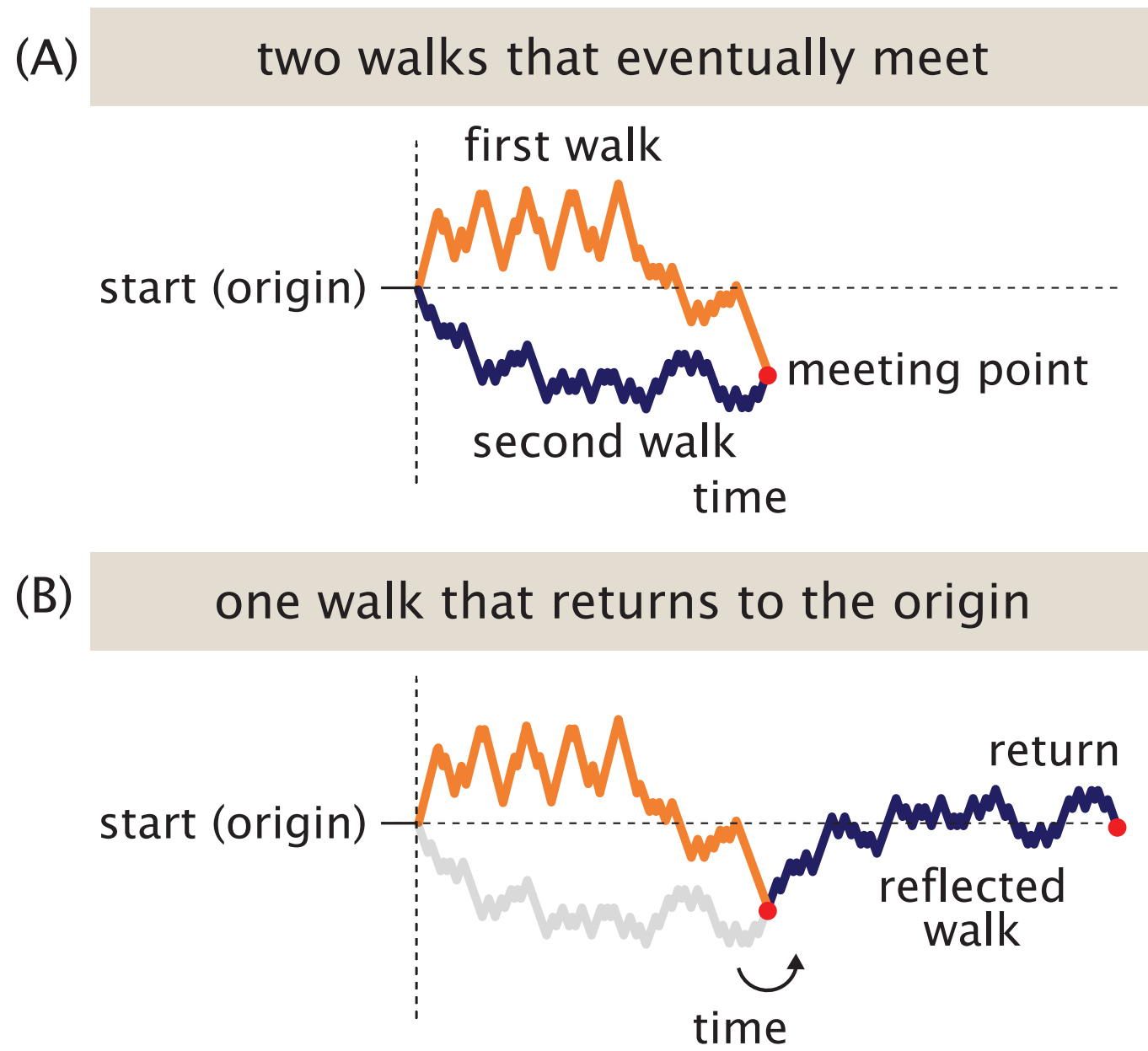

FIG. S1: Equivalence of the event that two separate random walks ever meet with the event that one random walk ever returns to the origin. (A) A pair of random walks (respectively, orange and navy) that meet at some point (in red). (B) Taking one of the random walks, swapping all positive steps for negative steps and vice versa, and appending to the other walk at the meeting point yields a new longer walk that accomplishes a return to the starting point.

infinity. (By marginal probability, we mean the probability of being at a certain position at a given time, regardless of whether or how many times this site has been visited before in a trajectory.) This is a useful wedge into the problem, because we are already armed with knowing how this marginal probability behaves. Intuitively, this decreases very steeply with the dimensionality d of the space the walk is performed in, since there are many more possible sites that a walker could be present at in spaces of larger dimension. Specifically this decay of probability steepens with dimensionality according to a power law prefactor $\sim t^{-d/2}$. Next we recall the other identifying characteristic of random walks: visiting sites a distance R away typically requires $\sim R^2$ timesteps, no matter the dimensionality; so that governing sum over all times essentially turns on only for times $t \gtrsim R^2$, meaning the whole sum can be approximated as something proportional to $\sim R^{2-d}$. This competition between the sites plausibly visited by random walks in any allotted time, and the (larger) d-dimensional volume of sites that are candidates to be visited, exposes the essence of why every site in spaces of dimensions higher than two is not certainly visited by random walks.

In slightly more detail: let $\mathbf{x}_T = [x_1, \ldots, x_d]$ be the location of a target of interest in d-dimensional space, relative to start at the origin, a distance $R = \sqrt{x_1^2 + x_2^2 + \cdots + x_d^2}$ away. The desired probability $p_{\text{hit}}(\mathbf{x}_T)$ that a walker *ever* hits $\mathbf{x}_T$ is complicated to think about. Yet, we already know many things about the marginal probability $p(\mathbf{x}_T, t)$ that a walk is found at the target $\mathbf{x}_T$ at some *particular* time t. Specifically, the probability $p_{d=1}(x_T, t)$ that a random walk is found at a particular one-dimensional position x_T is well approximated by a familiar normal distribution with mean position zero and variance t. (This behavior can be recalled simply by remembering that the walker steps left (giving a change in position $\Delta x = -1$) with equal probability as stepping right (giving $\Delta x = +1$), so the expected value of the squared update to position (yielding the variance) increases by 1 at each time step, while the expected (mean) position of the walk remains unchanged as zero; see also Appendix B for context for this behavior in terms of explicit lattice counts.) That is, we can write the probability density as

$$p_{d=1}(x_T, t) \approx \mathcal{N}\left(x_T \middle| 0, t\right) \tag{S1}$$

$$\approx \sqrt{\frac{1}{2\pi t}} \exp\left[-\frac{x_T^2}{2t}\right]. \tag{S2}$$

This statement is approximate in the same sense that the central limit theorem is approximate; it holds increasingly exactly for long walks t manifesting a continuous limit. In spaces of higher dimensionality $d > 1$, the time spent wandering is split among the d possible spatial coordinates. This means that any individual coordinate's position x_i at time t is set by a one-dimensional random walk typically with only $\approx t/d$ timesteps. Accordingly, each coordinate is well described by a normal distribution with

bioRxiv preprint doi: https://doi.org/10.64898/2026.09.16.750450; this version posted September 17, 2026. The copyright holder for this preprint (which was not certified by peer review) is the author/funder, who has granted bioRxiv a license to display the preprint in perpetuity. It is made available under a CC-BY 4.0 International license.

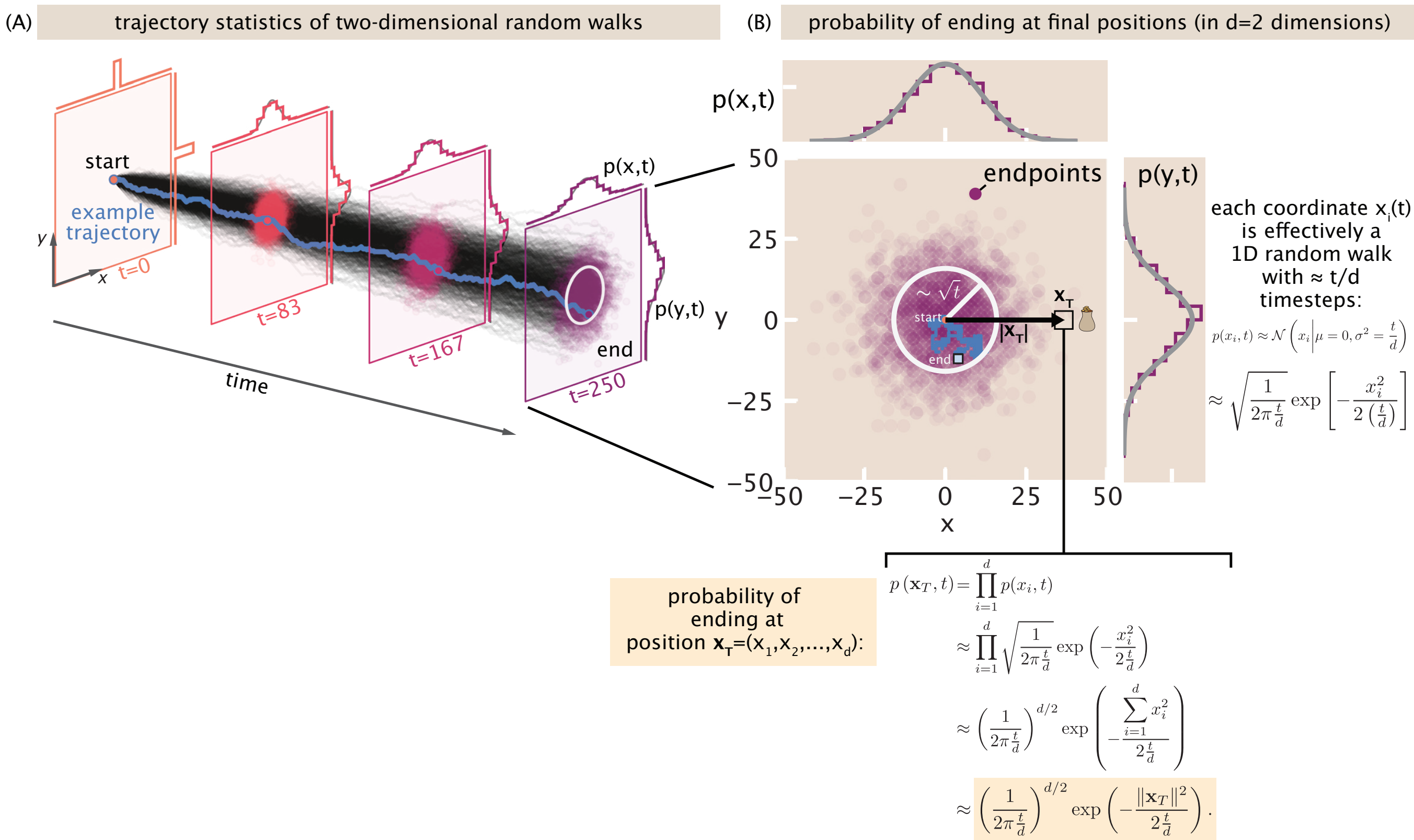

FIG. S2: Spreading of random walks over time and dimension. (A) A few thousand individual symmetric random walks illustrated in $d = 2$ dimensions visualized over time as black traces. A representative trajectory is shown in the pale blue line. The distributions of the positions of walks at a sequence of times are shown at the colored planes. (B) A representative temporal slice of the ensemble of random walks, corresponding to the final time in (A). Histograms (empirical in purple; theoretical, according to eqn. S4, in grey) show how each spatial coordinate effectively receives a fraction $1/d$ of t timesteps, thus effectively obeying an independent one dimensional walk's limiting normal distribution in position. Together these coordinates specify the distribution over d-dimensional positions.

variance t/d, or

$$p(x_i,t) \approx \mathcal{N}\left(x_i \middle| \mu = 0, \sigma^2 = \frac{t}{d}\right) \tag{S3}$$

$$\approx \sqrt{\frac{1}{2\pi\frac{t}{d}}}\exp\left[-\frac{x_i^2}{2\frac{t}{d}}\right]. \tag{S4}$$

This description for the marginal distributions of coordinate positions is illustrated for the case of $d = 2$ dimensions in Fig. S2. As a consequence, the vectorial position of walks in all d dimensions spreads radially over time, with a typical mean squared displacement of t or radius of $\sqrt{t}$. This is conspicuous in the widening ensemble of walks shown Fig. S2(A) in two dimensions, or the spreads visualized in one, two, and three dimensions in Fig. S3.

More algebraically, this behavior is explained by understanding that the probability of being at a vectorial position $\mathbf{x}_T$ is the product of the probabilities of finding each coordinate at their positions, over all d dimensions. As shown by Fig. S2(B), all this reasoning leads to the understanding that the marginal probability of finding a walk at position $\mathbf{x}_T$ at time t in d dimensions is,

$$p(\mathbf{x}_T,t) \approx \left(\frac{1}{2\pi\frac{t}{d}}\right)^{d/2}\exp\left(-\frac{\|\mathbf{x_T}\|^2}{2\frac{t}{d}}\right) \tag{S5}$$

$$= \left(\frac{d}{2\pi}\right)^{d/2} t^{-d/2}\exp\left[-\frac{dR^2}{2}\,\frac{1}{t}\right]. \tag{S6}$$

Importantly, this probability of finding a walker at a site a distance R from the origin is very small until times of order $t \gtrsim R^2$, after which the probability falls again, as witnessed by Fig. S4(A). This behavior reflects two competing physical effects. When

bioRxiv preprint doi: https://doi.org/10.64898/2026.09.16.750450; this version posted September 17, 2026. The copyright holder for this preprint (which was not certified by peer review) is the author/funder, who has granted bioRxiv a license to display the preprint in perpetuity. It is made available under a CC-BY 4.0 International license.

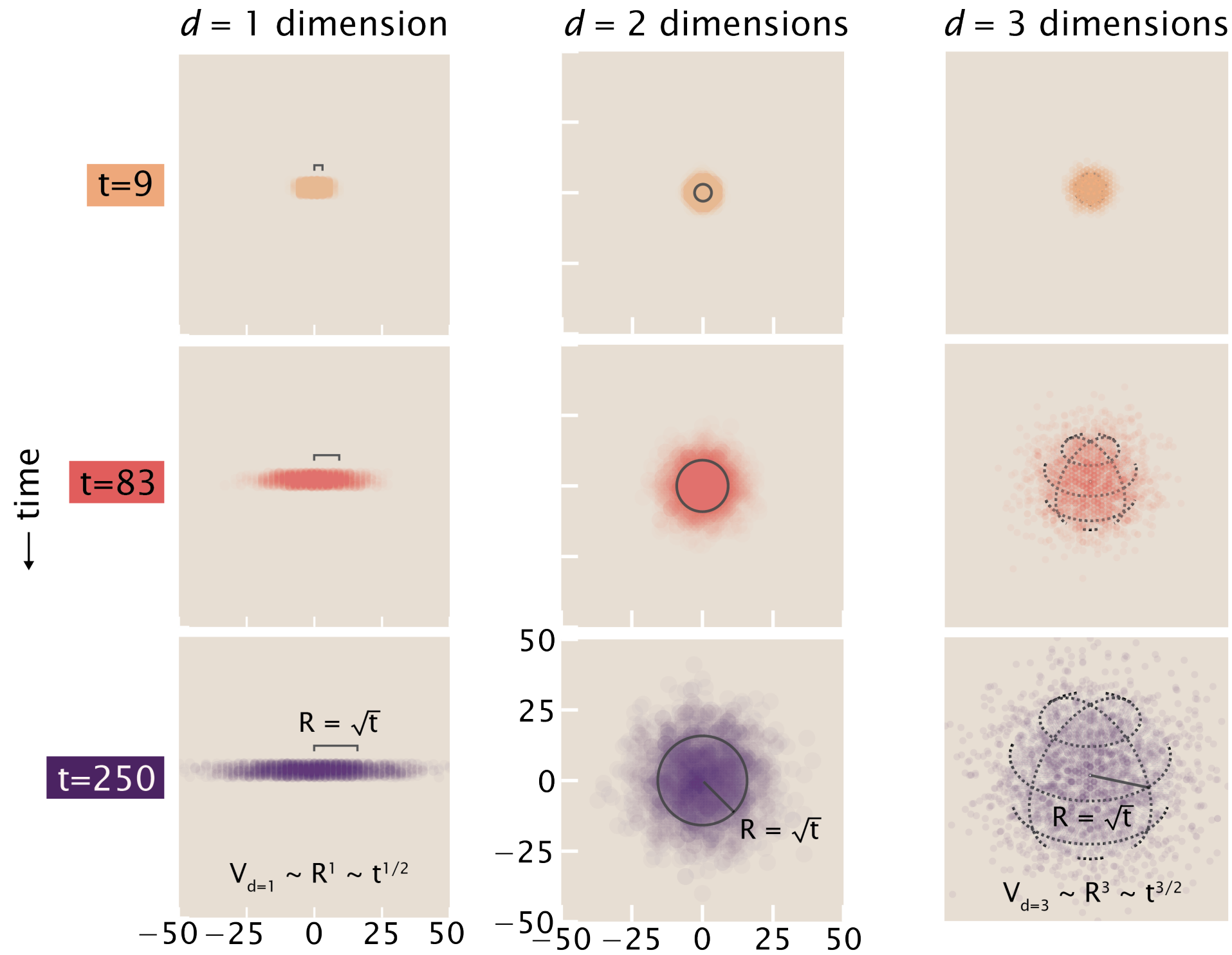

FIG. S3: Sampled distributions of positions of random walks in one, two, and three dimensional spaces over time.

t is too small, the target position is at too great a distance for walks to have meaningfully sampled; this effect is expressed by the dominance of the $\exp\left[-\frac{dR^2}{2}\,\frac{1}{t}\right]$ factor of eqn. S6, plotted in blue in Fig. S4(B). At long times, however, the fact that the walks have wandered over a burgeoning d-dimensional volume means that the probability that a walker is found at a particular site within this volume shrinks; this volume dilution is reflected by a dominance of the $t^{-d/2}$ factor, plotted in red in Fig. S4(B). The probability is peaked when these factors have fractional changes in time that match in size, as tracked by crossover in the logarithmic derivatives shown in Fig. S4(C).

Armed with this understanding given by eqn. S6, we return to tackle our big question of finding the desired probability that a walk ever visits such a target site at any time, $p_{\text{hit}}(\mathbf{x}_T)$. These quantities are related in the following simple sense. If we add up the (now, known) probabilities $p(\mathbf{x}_T,t)$ of the walk being at the target for all possible times t, we would get close to expressing the desired probability $p_{\text{hit}}(\mathbf{x}_T)$ of ever hitting the target. But we will have overcounted: for instance, this double-counts trajectories that are both present at the target at one time t and also another time t', which contribute to both the marginal probabilities $p(\mathbf{x}_T,t)$ and $p(\mathbf{x}_T,t')$ but actually contribute only once to $p_{\text{hit}}(\mathbf{x}_T)$, as visualized in the example of Fig. S5. This just equips us to safely write the upper bound,

$$p_{\text{hit}}(\mathbf{x}_T) \leq \sum_{t=0}^{\infty} p(\mathbf{x}_T,t). \tag{S7}$$

(More generally, this simple statement is just a particular instance of the general so-called "union bound" of probabilities, saying that the total probability $p(\cup_i A_i)$ of any of a set of k events $\{A_1,\ldots,A_k\}$ is at most the sum of their probabilities. For example, it is simplest to see this for the case of two events $\{A_1,A_2\}$: the probability of seeing either of these two events (their union) is $P(A_1 \cup A_2) = P(A_1) + P(A_2) - P(A_1 \cap A_2)$, or in pictures, 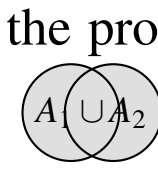 $=$ 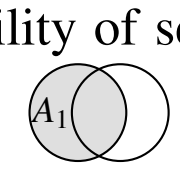 $+$ 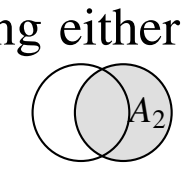 $-$ 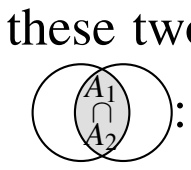: neglecting the latter intersection piece $P(A_1 \cap A_2)$, which has positive probability, gives an upper bound. In our setting, the events A_1 and A_2 can mean a walk being found at time t or t', respectively.)

Now we apply our understanding in eqn. S6 of how the marginal probabilities $p(\mathbf{x}_T,t)$ of finding a walk at position R behave over time: in effect, these are strongly suppressed for $t \ll R^2$, turn on for $t \gtrsim R^2$, and are modulated by a sharp power law in the

bioRxiv preprint doi: https://doi.org/10.64898/2026.09.16.750450; this version posted September 17, 2026. The copyright holder for this preprint (which was not certified by peer review) is the author/funder, who has granted bioRxiv a license to display the preprint in perpetuity. It is made available under a CC-BY 4.0 International license.

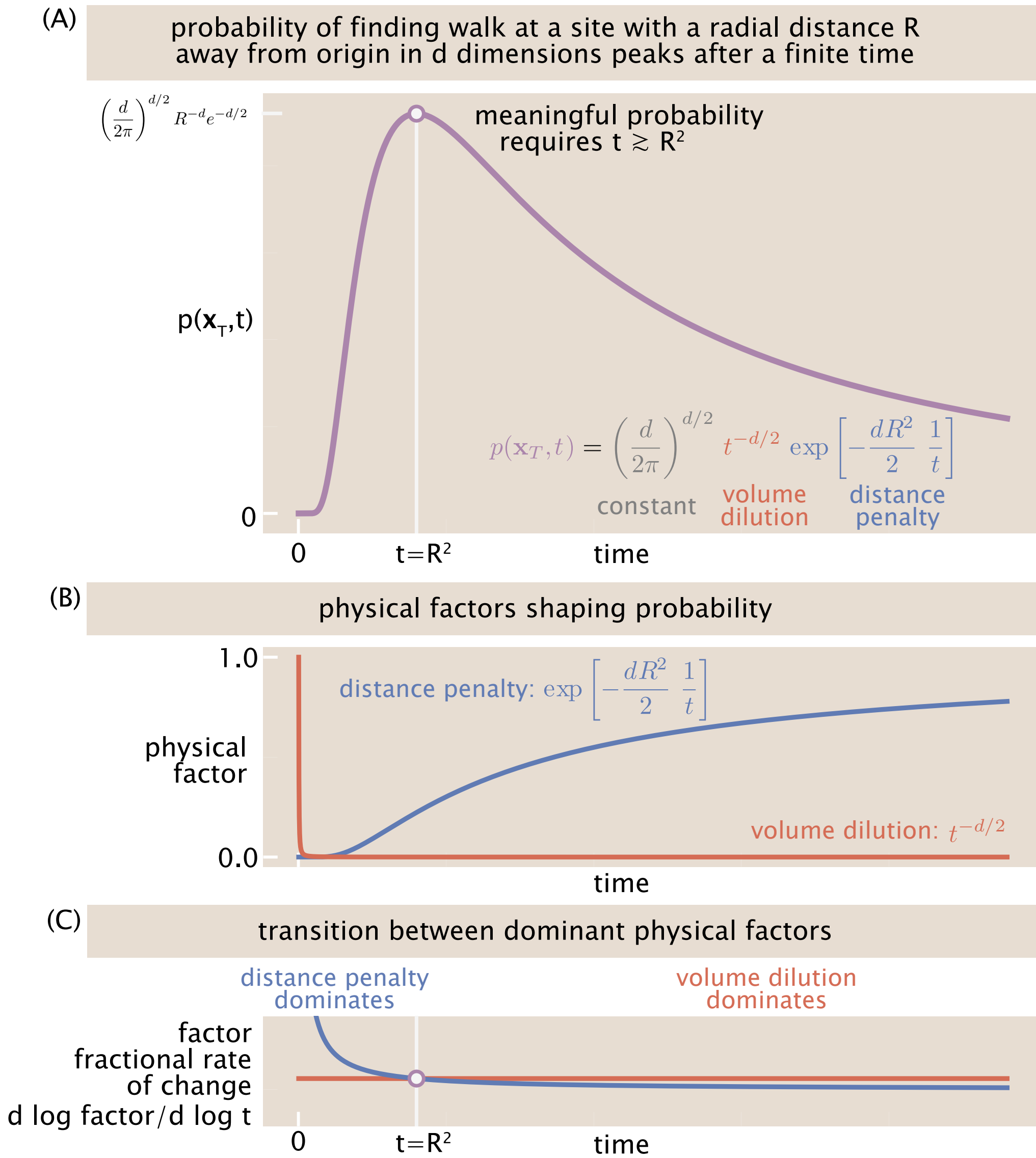

FIG. S4: The probability of a random walk occupying a site at a distance R from the origin in d dimensions. (A) The marginal probability $p(\mathbf{x}_T, t)$, as described by eqn. S6, is strongly peaked in time; observing a walk at a radial position R is small until a time $t \gtrsim R^2$, then declines again. (B) This probability is shaped by whether or not enough time has elapsed for walks to have a chance of reaching the radial position R, given by a distance penalty factor shown in blue, competing with the dilution over all possible sites walks could be found in the d-dimensional volume at time t, given by the volume dilution factor in red. (C) The dominance of these two effects switches at a peak time $t = R^2$; the fractional rates of change of these factors mark the crossover.

dimensionality d. This emboldens us to more daringly approximate the bound of eqn. S10 as,

$$p_{\text{hit}}(\mathbf{x}_T) \leq \sum_{t=0}^{\infty} p(\mathbf{x}_T, t) \tag{S8}$$

$$\leq \sum_{t=0}^{\infty} \left(\frac{d}{2\pi}\right)^{d/2} t^{-d/2} \exp\left[-\frac{dR^2}{2}\,\frac{1}{t}\right] \tag{S9}$$

$$\lesssim C_d \sum_{t=R^2}^{\infty} t^{-d/2}. \tag{S10}$$

The replacement in the last line imposes two core features we observed in Fig. S4 and reflects a scaling behavior (not a pointwise relation). First, the exponential distance penalty $\exp\left[-\frac{dR^2}{2}\,\frac{1}{t}\right]$ acts as a soft threshold function in time, making it very unlikely

bioRxiv preprint doi: https://doi.org/10.64898/2026.09.16.750450; this version posted September 17, 2026. The copyright holder for this preprint (which was not certified by peer review) is the author/funder, who has granted bioRxiv a license to display the preprint in perpetuity. It is made available under a CC-BY 4.0 International license.

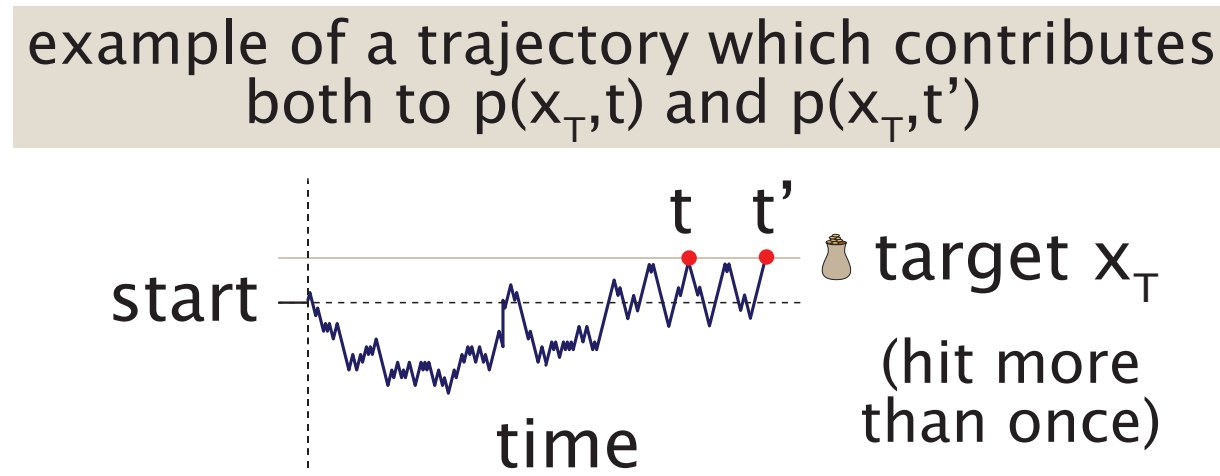

FIG. S5: Example of a trajectory which makes the union bound loose, namely, eqn. S10 generally a strict inequality.

to find a walk at the radial position R much before time $t \lesssim R^2$. Second, after this peak time, the exponential distance penalty's effect saturates and does not change the main scaling behavior (as shown in Fig. S4(B)). More precisely, we can squeeze it between two simple constants that depend only on the dimensionality, namely $\exp[-d/2] \leq \exp\left[-\frac{dR^2}{2}\frac{1}{t}\right] \leq 1$; so the behavior of the whole probability function is dominated by the $t^{-d/2}$ volume dilution behavior, up to some approximately constant factor C_d close to one. That is, the distance penalty $\exp\left[-\frac{dR^2}{2}\frac{1}{t}\right]$ is bounded from above and below by constants that are close to one, and just depend on the dimensionality d of the space the random walk explores. These behaviors are visualized in Fig. S6. The takeaway is that the remaining $t^{-d/2}$ behavior dominates the time dependence after the peak, $t > R^2$.

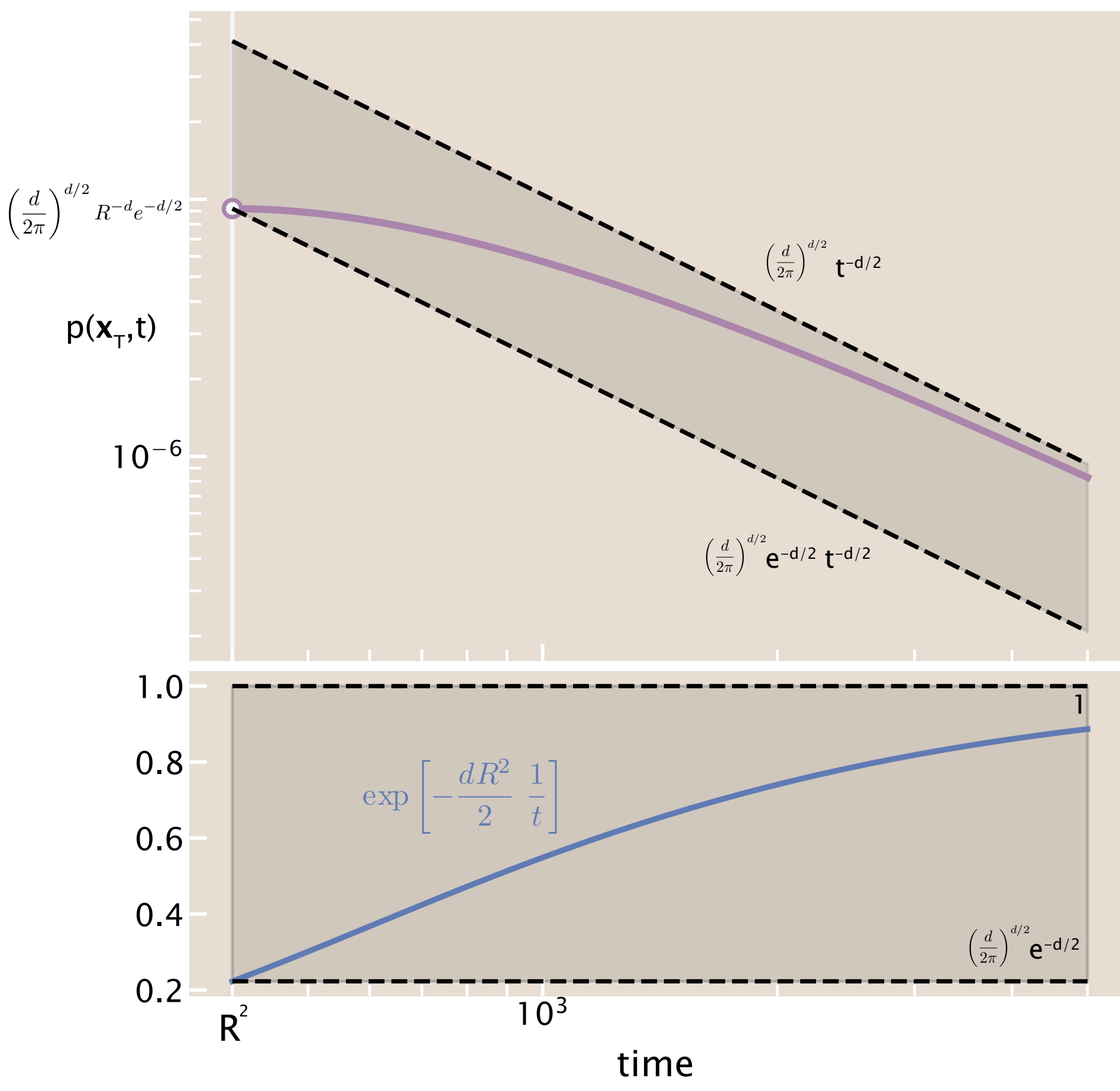

FIG. S6: Illustration of the bounds sandwiching the exponential distance penalty, $\exp[-d/2] \leq \exp\left[-\frac{dR^2}{2}\frac{1}{t}\right] \leq 1$. For illustration, this plot reflects the same purple and blue curves as in Fig. S4(A) and (B) of the main text except for times appearing just after the peak $t \geq R^2$ and with axes on a log-log basis to illustrate the agreement in power law exponents. Specifically, these plots reflect the case of a $d = 3$ dimensional space at a target located at a site $R = 25$ away from the origin.

Next, to appreciate the core behavior of the summation in Eqn. S10, we realize it adopts the form of a Riemann sum and can

bioRxiv preprint doi: https://doi.org/10.64898/2026.09.16.750450; this version posted September 17, 2026. The copyright holder for this preprint (which was not certified by peer review) is the author/funder, who has granted bioRxiv a license to display the preprint in perpetuity. It is made available under a CC-BY 4.0 International license.

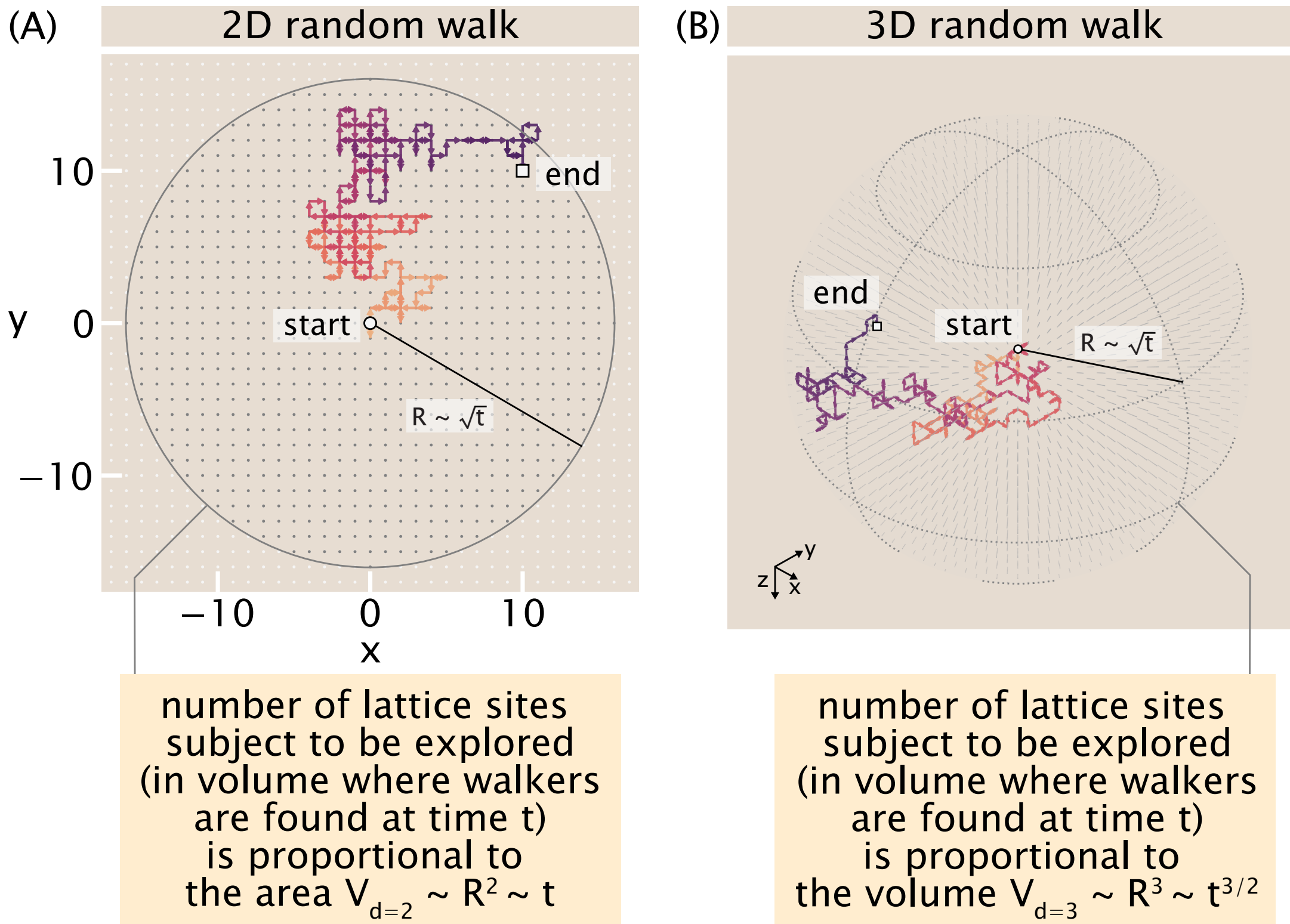

FIG. S7: The number of spatial locations standing to be explored at time t explodes with dimensionality. This growing set of lattice sites standing to be explored are shown by gray dots found within a radius $R \sim \sqrt{t}$, describing the region where walkers are expected to reside.

be expressed as approaching an integral (particularly for distant targets with large R), giving,

$$p_{\text{hit}}(\mathbf{x}_T) \lesssim C_d \int_{R^2}^{\infty} t^{-d/2}\, dt \tag{S11}$$

$$\lesssim C_d'\, R^{2-d}, \tag{S12}$$

with C_d' some suitable constant.

At last, eqn. S12 exposes the critical dimensionality-dependence of recurrence or the probability of ever hitting a target. When $d > 2$, the power law behavior $\sim R^{2-d}$ definitely shrinks to zero for sites with large enough R: this is enough to establish that these sites are not visited with certainty. (By itself, note that this expression eqn. S12 does *not* yet comment forcefully that the optimistic converse is true for $d = 1$ or $d = 2$ dimensions, since the integral diverges (giving a vacuous bound on a hitting probability); additional theoretical ingredients are needed to appreciate that certain arrivals do happen in these lower dimensions.)

The foregoing analysis justifies, intuitively but concretely, why random walks explore so differently in one or two dimensional spaces, compared to higher dimensional spaces. Another vantage on this core transition in behavior with respect to dimensionality is to ask about the number of new sites actually visited, relative to the number of sites that could feasibly be visited, up to time t, for random walks in various dimensions. To estimate the number of candidate sites to be explored, as we have argued, the typical region where a random walk is found has a characteristic radial size of $R \sim \sqrt{t}$. This means that random walks in dimensionality d explore a volume

$$V_d \sim R^d \sim t^{d/2} \tag{S13}$$

of possible candidate sites. As visualized for the humble cases of two and three dimensions in Fig. S7, this volume of candidate sites to be explored grows very rapidly with dimensionality d. In stark contrast, the largest number of new sites a random walk can ever visit up to a time t is at most t (when every timestep leads to a new site). This competition between the limited rate of actually visiting fresh sites (optimistically linear in t) compared to the exploding volume of them that stands to be visited (a power law with exponent $d/2$ in dimension d) already anticipates that the latter can outpace the former near a marginal

bioRxiv preprint doi: https://doi.org/10.64898/2026.09.16.750450; this version posted September 17, 2026. The copyright holder for this preprint (which was not certified by peer review) is the author/funder, who has granted bioRxiv a license to display the preprint in perpetuity. It is made available under a CC-BY 4.0 International license.

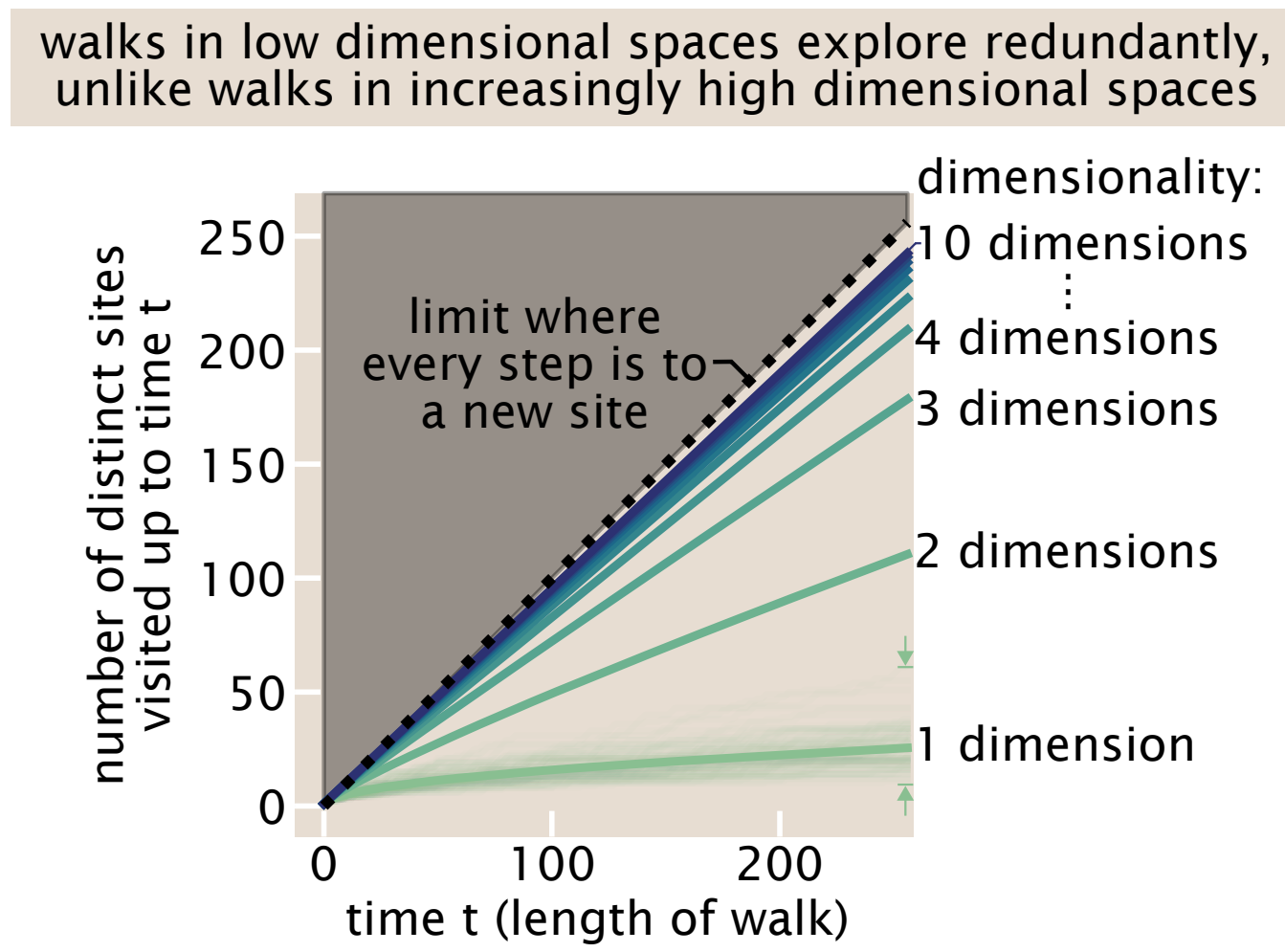

FIG. S8: Average coverage of new sites by random walks over time in spaces of varying dimensionalities. Specifically, for each dimensionality, 2400 random walks were performed; in each walk, the number of unique lattice sites visited up to time t was counted, and the averages over these walks are reported as thick solid lines. (As a guide to the eye, the variability in this coverage is shown in the set of individual stochastic coverages in one dimension, shown as faint thin lines; arrows mark the extent of this variability.)

dimension around $d = 2$. Making this point precise, define the expected density of visited sites in d-dimensions as $\rho_d(t)$: then,

$$\rho_d \leq \frac{t^1}{t^{d/2}} \sim t^{1-d/2}, \tag{S14}$$

which evinces that the density of visited sites decreases in time for $d \geq 3$.

In fact, the situation is more extreme than this picture captures: bare random walks do not sample fresh sites as fast as a linear rate in time. Instead, only a fraction of timesteps yield exposure to a new site not yet visited before, and this coverage rate itself depends heavily on dimension. This is shown explicitly by simulation in Fig. S8, where we perform walks in spaces up to ten dimensions, track the number of new locations sampled up to a given time t, and average over thousands of walks. Strikingly, particularly in spaces of low dimension such as $d = 1$ or $d = 2$, walks explore sites very thoroughly and redundantly as stressed by the starkly sublinear coverage curves. Only in spaces of more than a few dimensions does coverage begin to substantially resemble the ideal limit where every step leads to a fresh location. However, this coverage can still never exceed the speed limit of the $\sim t^1$ scaling shown as a black dotted line in Fig. S8. The fundamental gulf between the fresh coverage a walk could ever expect to have, and the number of sites standing to be explored, expresses the essence of why bare random walks in high dimensional spaces may fundamentally fail to reach a target.

1. Impact of confinement and finite system sizes

We just earned a feel for why unbounded random walks take an infinite time to reach a finite target, and why the probability of hitting a target becomes lower than one in spaces of sufficient dimensionality. However, an immediate, reasonable, and rich question arises. How does the effectiveness of random search change when we acknowledge that cells and organisms are not unbounded systems (for which we should be grateful), but rather have finite sizes?

Of course, like many cells, the Zurich that Pólya walked in and found motivating was also finite. The original idealization to an infinite grid was very intentional: in Pólya's own words, "If the question is conceived too narrowly, too realistically, *au pied de la lettre*, with the actual data about the network of winding footpaths behind the [Zurich] hotel, it becomes unmanageably complicated and, moreover, uninteresting. Yes, the problem must be simplified. . .The boundary of such a regular network may be irregular—but let the city be very big, unlimited: this is the simplest case and the most interesting, too"[22]. Whether a cell or organism regards the math of infinite grids indeed "most interesting," or instead feels more strongly finite-size effects imposed by confinement, depends on the degree of confinement. This surely varies richly across the spectrum of biology between systems that are small enough for random walks to search well enough, to those systems that are large or high-dimensional enough for further mechanisms to be helpful.

Technically, any such finite system size makes visiting a target site bound to happen eventually (giving $p_{\text{hit}}(\mathbf{x}_T) = 1$) for any dimensionality, at least if given enough time, in contrast to the recurrence probability behaviors discussed earlier for searches in

bioRxiv preprint doi: https://doi.org/10.64898/2026.09.16.750450; this version posted September 17, 2026. The copyright holder for this preprint (which was not certified by peer review) is the author/funder, who has granted bioRxiv a license to display the preprint in perpetuity. It is made available under a CC-BY 4.0 International license.

infinite spaces. Such confinement also makes the mean time to arrive at a target finite.

However, biological explorations must also occur within times acceptable to organisms. For instance, to elicit its function, a protein diffusing in a cell must encounter its destination or binding partner before being degraded (by active machinery or passive injury). Similarly, a starving bacterium or worm crawling through soil is in a race against time to encounter nutrients before their internal reserves run out. Growing neurons must encounter their synaptic partners in tolerable developmental times. Limited time also constrains decisions in higher dimensional space of cognitive behaviors. Accordingly, it is more revealing to ask about how finite physical sizes affect biological searches while acknowledging their accompanying finite time limits.

First, consider the case of one dimension: how does confinement matter? Happily, this question is already analyzed by the main text's analysis of the heuristic that random walks with reset can be approximated as walking within a reflecting box. This led to eqn. 35 stating that in a one dimensional box where a reflecting boundary is located at $x = -L_R$, a target is located at $x = x_T$, and a random walk begins at the origin $x = 0$, the expected time to first hit the target is

$$T(0) = \frac{x_T^2}{2D} + \frac{L_R x_T}{D} \tag{S15}$$

$$\approx \frac{L_R x_T}{D}. \tag{S16}$$

While the main text used the distance L_R to reason about the effect of resets, here we can instead take this to mean the characteristic lengthscale of confinement we are interested in understanding the impact on. In summary, then, in one dimension, keeping the target location x_T fixed but expanding the domain of confinement L_R makes the average time to hit the target increase approximately linearly with L_R. Or else if the target's location is a fixed fraction of the system size $\sim L_R$ then as the system's size grows, the time to hit the target grows quadratically with the system size. Specifically, when the location of the target x_T is some fixed fraction α of the distance L_R from the origin to the reflecting wall, namely $x_T = \alpha L_R$, then, $T(0) = \alpha\left(1+\frac{\alpha}{2}\right)\frac{L_R^2}{D} \propto \frac{L_R^2}{D}$ that is, varying with the characteristic L_R^2/D timescale one can anticipate just on dimensional grounds.

Some biological systems enjoy a small enough characteristic size (and thus L_R) that the time to encounter targets is functionally acceptable: for instance, small molecules (diffusing at $D \gtrsim$ few $\mu\text{m}^2/s$ in a cytosol) move across the length of a bacterium $L_R = 1\ \mu m$ in a few tenths of a second, or a typical mammalian tissue cell with $L_R = 10\ \mu$m in a few tens of seconds. It takes over an hour for such a molecule to diffuse across a human egg cell with $L_R \approx 120\ \mu$m. It would take almost a day to diffuse across a *Drosophila* embryo of length $L_R = 500\ \mu$m (comparable to the full time it actually takes for the larva to develop and hatch!), and if axons relied on diffusion alone to operate then transport of one molecule along their few centimeter lengths would take of order 10 years. In sum, even for one dimensional systems in physical space, some systems are small enough for bare random walks to be acceptable and others motivate the introduction of mechanisms such as drift (directed transport by motors), cues, and other contrivances.

Incidentally, this calculation also gives us a route to understand when resets can still be dynamically relevant when the biological space already enjoys confinement. Specifically, two length scales compete—the resets impose a lengthscale $L_{R,\text{reset}}$ and the system's size imposes a lengthscale $L_{R,\text{system}}$. If the system is already confined to a small enough $L_{R,\text{system}}$ then the resets may be slow enough (giving a long enough $L_{R,\text{reset}}$) not to matter significantly. Specifically, in this one-dimensional context, we can compare the average time T_{reset} to reach the target when the search is effectively confined by resetting with the time T_{system} when it is instead confined by the finite size of the system. These times compare according to the ratio,

$$\frac{T_{\text{reset}}}{T_{\text{system}}} \approx \frac{\frac{L_{R,\text{reset}} x_T}{D}}{\frac{L_{R,\text{system}} x_T}{D}} \tag{S17}$$

$$\approx \frac{L_{R,\text{reset}}}{L_{R,\text{system}}} \tag{S18}$$

$$\approx \frac{C\sqrt{DT_R}}{L_{R,\text{system}}}. \tag{S19}$$

This makes clear that resetting matters in shortening the arrival time to the target ($\frac{T_{\text{reset}}}{T_{\text{system}}} \lesssim 1$) exactly when,

$$T_R \lesssim \frac{L_{R,\text{system}}^2}{D}. \tag{S20}$$

Namely, in one dimension, reset can be relevant to help when the interval between resets is shorter than the characteristic time to diffuse across the system's finite length.

bioRxiv preprint doi: https://doi.org/10.64898/2026.09.16.750450; this version posted September 17, 2026. The copyright holder for this preprint (which was not certified by peer review) is the author/funder, who has granted bioRxiv a license to display the preprint in perpetuity. It is made available under a CC-BY 4.0 International license.

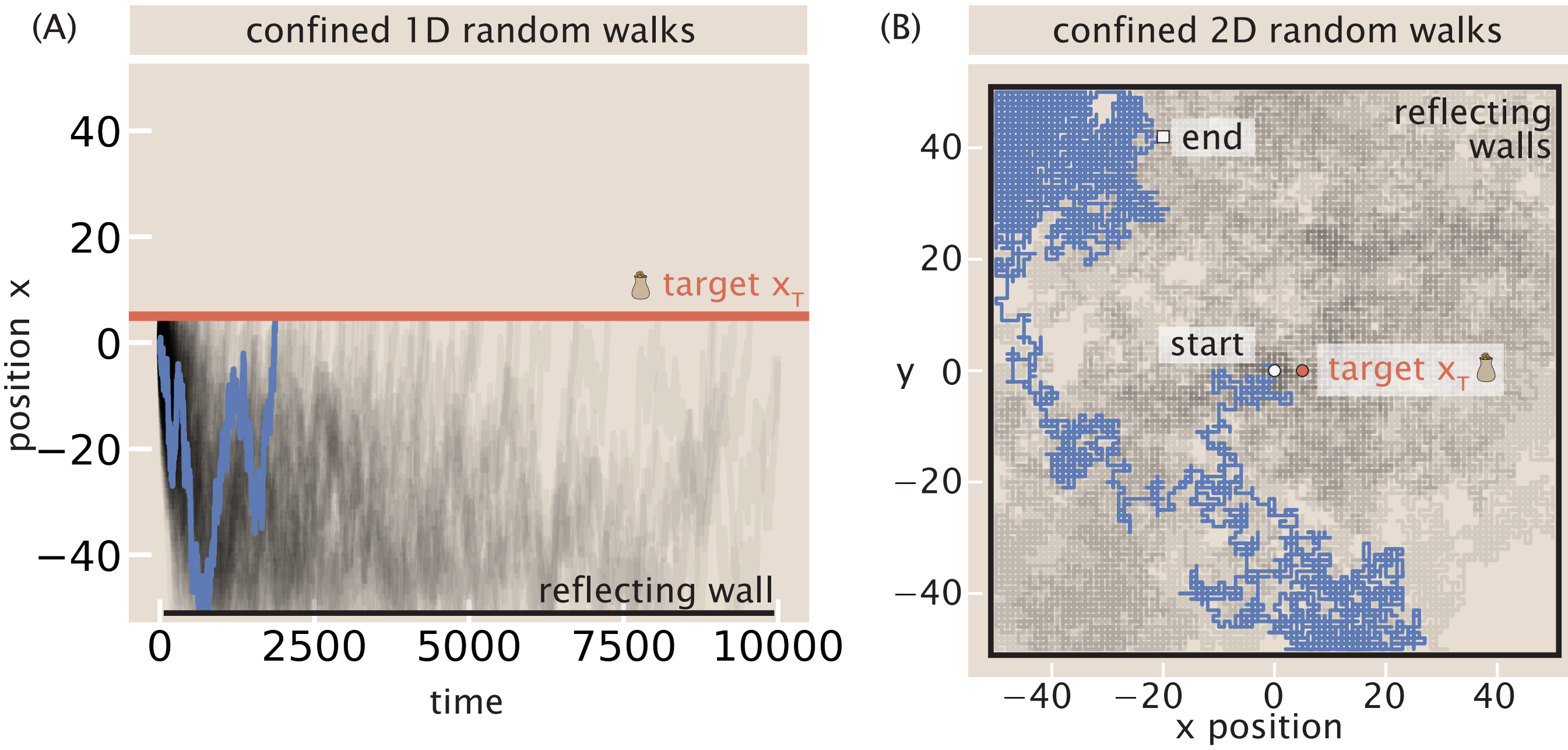

FIG. S9: Random walks in finite domains. This visualizes the confined case with $L_R = 50$ in each coordinate dimension, small enough that walks can interact with the barrier before first arriving at the target. A target at $x_T = 5$ in 1D (A), or at $\mathbf{x}_T = [5,0]^\top$ in 2D (B), is shown as a red line or a red dot, respectively. Every walk displayed is allowed up to $T = 10000$ steps to find the target if it does so.

In higher dimensional spaces, such as two-dimensional or three-dimensional positions or larger state and behavioral spaces, the strong impact of dimensionality hinted at from our infinite-systems discussion persists in essence. To give a feel for such confined search processes, Fig. S9 illustrates simulated trajectories in boxes of size $L_R = 50$ in one or two dimensions, with a target located only $x_T = 5$ units away from the starting position of random walks. This system size is small enough for walks to often interact with the reflecting boundaries before reaching the target, as visualized by two representative walks in blue in Fig. S9(A) and (B).

How many such walks reach the target five units away from the start in spaces of increasing dimensionality? Fig. S10 reports the fraction of such fortunate walks under various budgets in allotted time. While in one dimension, nearly all walks arrive at the target within a generous time $t = 10,000$ steps (as visualized by the dark purple line in Fig. S10), this drops to just above half in $d = 2$ dimensions and only less than 6% of such walks arrive at the target in three dimensions. More constrained time budgets (still large compared to the shortest possible time of $t = 5$ required of the luckiest trajectories ballistically arriving at the target) drive this success probability dramatically down, as visualized by the lighter orange lines in Fig. S10. In summary, these exercises affirm that even in confined spaces, larger dimensionalities of spaces can punish the effectiveness of ordinary random walks.

bioRxiv preprint doi: https://doi.org/10.64898/2026.09.16.750450; this version posted September 17, 2026. The copyright holder for this preprint (which was not certified by peer review) is the author/funder, who has granted bioRxiv a license to display the preprint in perpetuity. It is made available under a CC-BY 4.0 International license.

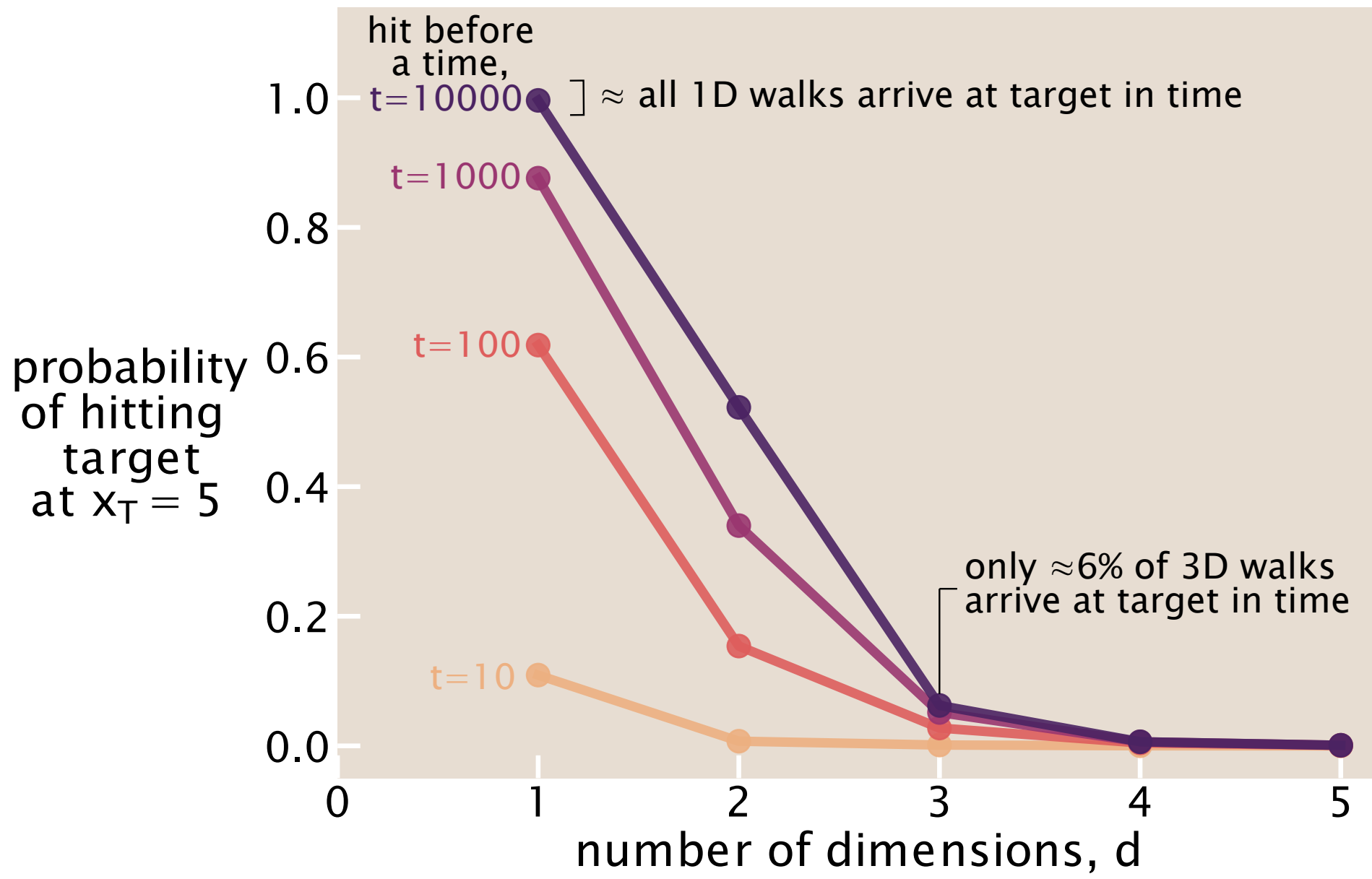

FIG. S10: Random walks in finite domains of varying dimensionalities. A target is localized at $\mathbf{x}_T = [5, 0, 0, \ldots, 0]$ in d-dimensional space. For each dimensionality d, an ensemble of 10000 random walks was performed, and the fraction of these walks that had hit the target by each allotted time is reported. The lines of increasingly orange color from top to bottom indicate the allowed times $t \in \{10, 100, 1000, 10000\}$ steps. (The width of the reflecting box was $L = 50$ in each coordinate dimension.)

2. Conclusions about bare random-walks

Taken together, these stark mathematical behaviors could explain why biological systems often do not rely on ordinary random walks alone. Crucially, the behavioral and phase spaces that biological searchers explore are often much richer than literal (one, two, or three dimensional) physical positions. Cells express thousands of genes and so gene expression traverses spaces of thousands of dimensions; epigenetic modifications on polymers explore combinatorial spaces; the cytosolic soup bubbles with thousands of distinct chemical species (and adjusts their counts in regulatory spaces of comparable dimensionality). At a higher level, animal behaviors, let alone underlying cognition, are set by the exquisite coordination of myriad degrees of freedom. Accordingly, instead of such bare random walks, biological searchers may use superficially surprising modifications to this type of motion to accomplish searches much more effectively, as discussed with the case studies of resets, drift, cues, and checkpoints, as shown in the main text.

3. Helpfulness of resets in random walks of higher dimensionality

The formidable influence of dimensionality on random walks also raises the significance of how resets might accelerate the time to encounter targets, as explored in the case of $d = 1$ dimension in §III in the main text. Since the failure of a seeker to ever find a target at all (or within a feasible time) is more likely in higher dimensional spaces, we might expect that the potential remedies of resets could give even starker benefits there than in lower dimensional spaces. Indeed, Ref.[11] insightfully analyzed a lattice model to find the optimal rates for reset in d dimensions and found that more aggressive reset rates, approximately linearly scaling with d, manifest in higher dimensions. Much more thinking is warranted on such dynamics and policies, and whether richer effects unique to higher dimensional spaces emerge. But we expect these provocations are sure to be relevant to the large state, parameter, and behavioral spaces of biology.

B: First-passage properties of ordinary random walks

Here, we discuss transparently and explicitly how the probability of a random walker trajectory reaching a target for the first time can be calculated by an exact enumeration of the relevant lattice paths. Our spirit here is to be very concrete and explicit, and to provide details, illustrations, and remarks usually omitted or left implicit as homework for the reader in other wonderful

bioRxiv preprint doi: https://doi.org/10.64898/2026.09.16.750450; this version posted September 17, 2026. The copyright holder for this preprint (which was not certified by peer review) is the author/funder, who has granted bioRxiv a license to display the preprint in perpetuity. It is made available under a CC-BY 4.0 International license.

foundational literature. Specifically, the approach shown here has an august history dating to Feller, Andre, Chandrasekhar, and other giants of probability.

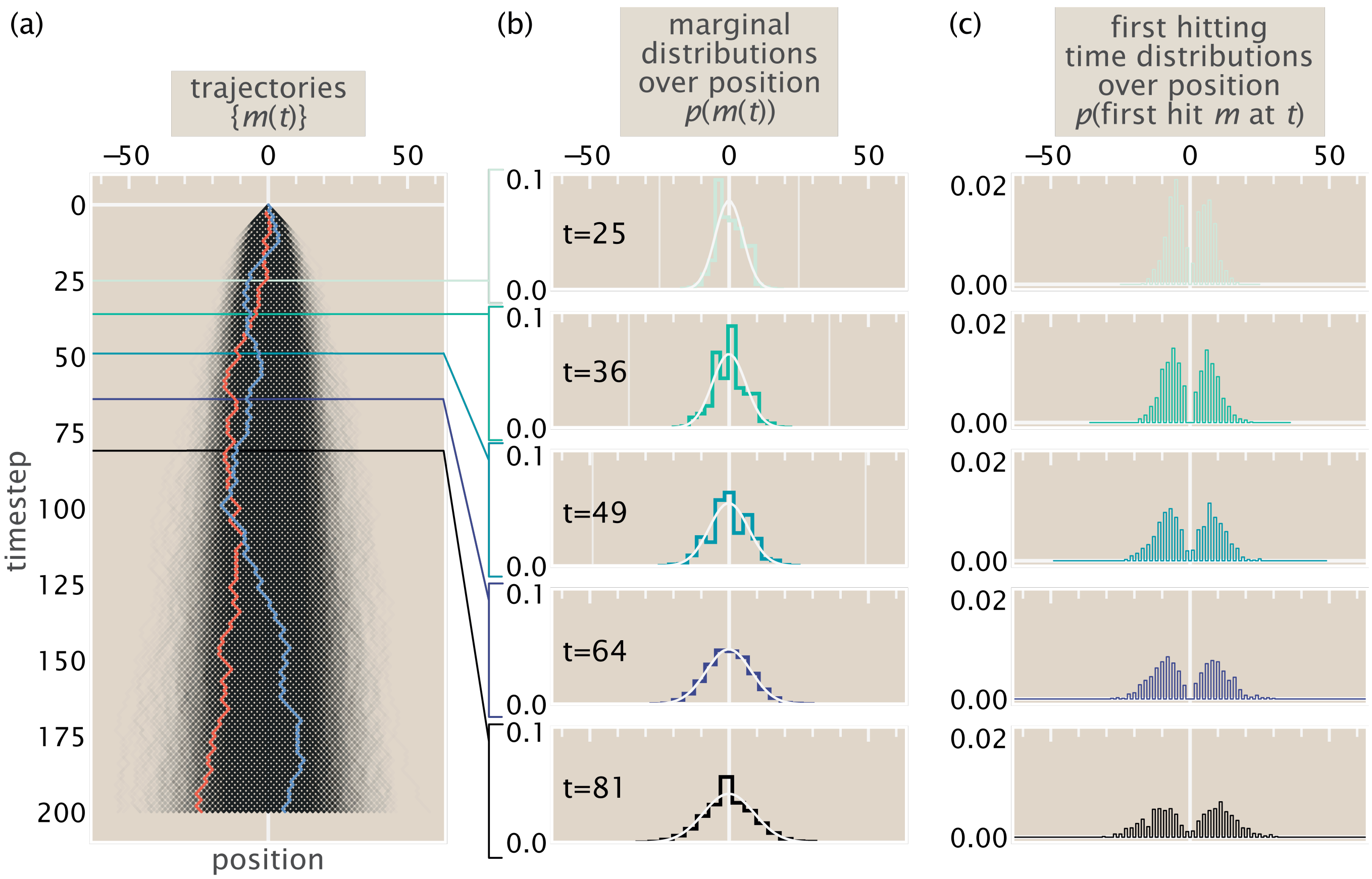

FIG. S11: Unbiased random walks in one dimension. (a) Trajectories of an ensemble of 8000 random walks over time (drawn in black, except for two arbitrary exemplary trajectories in blue or red, which respectively typify trajectories that succeed at visiting a target at $m = 10$ or fail to do so within the indicated time window). (b) The marginal probability $p(m(t))$ of finding walkers at position $m(t)$ sliced at timepoints $t \in \{5^2, 6^2, 7^2, 8^2, 9^2\}$ (shown as increasingly deeply teal colored histograms corresponding to the horizontal lines in (a)); these timepoints are quadratically spaced to show how the variance scales approximately quadratically in time. The corresponding Gaussian density approximation with the corresponding mean and variance of the distribution is shown as a light white line for reference. (c) The empirical fraction of trajectories that hit position m for the first time at time t for these same example timepoints in (b). (Note the even-odd parity oscillation: if m and t have different parities, the probability of first hitting m at time t is zero.)

What is the probability the walker is found at a position m at time t? This net position is the difference between the number of rightwards steps R accumulated up to time t, and the number of leftwards steps L up to time t, namely

$$m(t) = R - L;$$

and since every step must be right or left,

$$t = R + L.$$

(Accordingly, note that $R = \frac{t+m}{2}$ and $L = \frac{t-m}{2}$: the latter expression requires that the total time t and the net position m must be both even or both odd, since their difference divided by two must be even because L is an integer.)

The R independent right steps have each occurred with probability p, and the left steps each with probability $1-p$; a particular sequence of these steps thus has probability $p^R(1-p)^L = p^R(1-p)^{t-R}$. However, there are many ways of arranging these total number of rightwards and leftwards steps; specifically there are $\binom{t}{R} = \binom{t}{L}$ ways, so the total probability of all ways to take R

bioRxiv preprint doi: https://doi.org/10.64898/2026.09.16.750450; this version posted September 17, 2026. The copyright holder for this preprint (which was not certified by peer review) is the author/funder, who has granted bioRxiv a license to display the preprint in perpetuity. It is made available under a CC-BY 4.0 International license.

steps to the right in a total trajectory of length t is

$$\mathbb{P}(X(t) = m) = \mathbb{P}(R - L = m) \tag{S21}$$
$$= \mathbb{P}(R \text{ steps among } t) = \binom{t}{R} p^R (1-p)^{t-R} \tag{S22}$$
$$= \binom{t}{\frac{t+m}{2}} p^{\frac{t+m}{2}} (1-p)^{\frac{t-m}{2}} \tag{S23}$$

Naturally, this is the Binomial distribution with success parameter p for t trials; it has mean $\langle m(t) \rangle = tp$ and variance $\sigma_m^2 = \langle m^2 \rangle - \langle m \rangle^2 = tp(1-p)$. For familiar reference, we plot an ensemble of unbiased (e.g. $p = 1/2$) random walks in Figure S11(A).

1. First passage times by lattice counts

Next we ask a different question than the probability of the walker's instantaneous position: what is the probability that the walker visits site m *for the first time* at time t? To simplify this discussion, we now specialize to the unbiased walk case $p = 1/2$, which clarifies our problem as one of purely *counting* desirable trajectories (that successfully terminate at m but never have visited it before time t).

To proceed, we choose to generalize the reasoning involved in a popular proof for the form of the Catalan numbers, commonly attributed to André[14] (p. 276-278, ch. 9) and (separately) Feller[23] (p. 138, §A2). To lay the groundwork for our argument, as a clearer pedagogical example, we first give a very brief account of this tactic here.

The Catalan numbers count special paths. The Catalan numbers are a sequence of numbers that show up with spooky regularity across counting problems that look completely different from each other ([14,23]). The nth Catalan number is defined as,

$$C_n \equiv \frac{1}{n+1}\binom{2n}{n} \tag{S24}$$
$$= \binom{2n}{n} - \binom{2n}{n-1}. \tag{S25}$$

One beautiful combinatorial interpretation of the nth Catalan number is the number of lattice paths of length $2n$ connecting an origin $(0,0)$ to a point (n,n) at an upper right corner of a two-dimensional grid, in such a way that the paths do not cross (but may touch) the $y = x$ diagonal, and where paths consist only of up $(0,+1)$ or right $(+1,0)$ steps. These path requirements are visualized in Fig. S12, where an example of a good (valid) path not crossing the $y = x$ diagonal is shown in purple and a bad path that crosses this diagonal is shown in light orange.

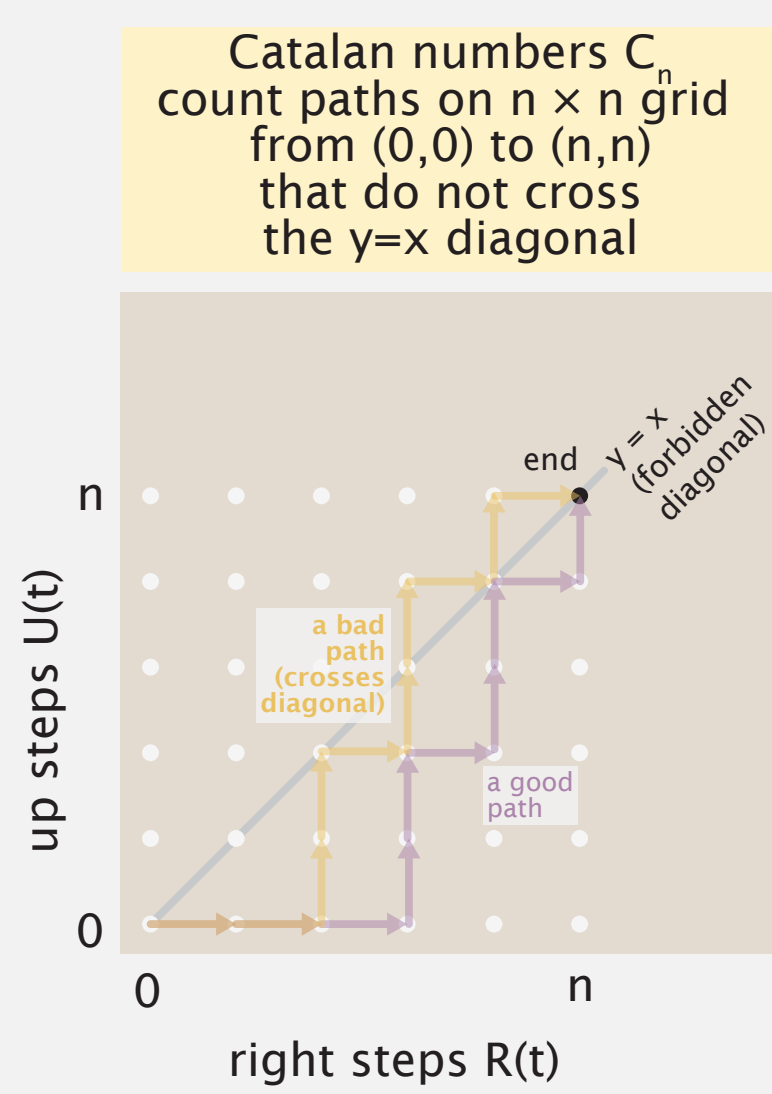

FIG. S12: Examples of good paths counted (and bad paths not counted) by the Catalan numbers.

bioRxiv preprint doi: https://doi.org/10.64898/2026.09.16.750450; this version posted September 17, 2026. The copyright holder for this preprint (which was not certified by peer review) is the author/funder, who has granted bioRxiv a license to display the preprint in perpetuity. It is made available under a CC-BY 4.0 International license.

These special lattice path objects seem like a very annoying set to count tidily; it is not obvious that they would somehow be accounted for by the concise algebraic definition in eqn. S24. However, the equivalent definition eqn. S25 of the Catalan numbers gives clearer understanding. Specifically the subtraction in eqn. S25 anticipates that the desired special objects can be accounted for by an easy count of a lager set of paths they belong to, minus some bad paths that violate their particular requirements. (This tactic will recur in later throughout our random walk analysis.)

To count these special permitted paths, the number of "bad" paths (that touch the diagonal prematurely at least once) can be subtracted from the total number of paths from $(0,0)$ to (n,n) (good or bad). This latter total number of paths is straightforward to count, being simply the number of ways of choosing n rightwards steps among the total number of steps $2n$, namely $\binom{2n}{n}$. The essential trickiness afflicts the number of bad paths. André (or Feller's) beautiful gambit, sometimes known as the *reflection method*, is to count these bad paths by building a one-to-one mapping to *another* set of paths that are much easier to count. We illustrate the important steps of this approach in Figure S13.

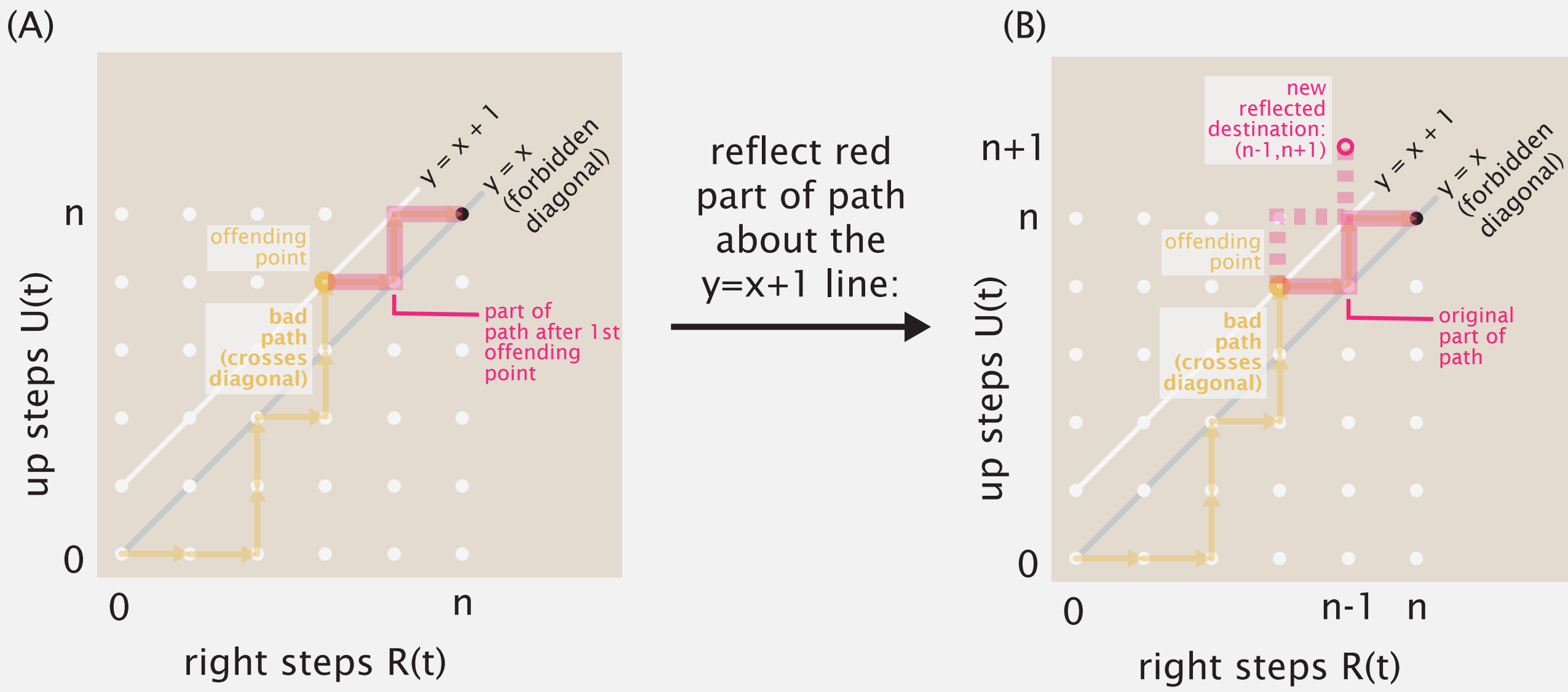

FIG. S13: Reflection procedure to count bad paths to exclude from Catalan counts. (a) Divide a bad path into parts before and after the point ○ after it has first offendingly crossed the $y = x$ diagonal (the former subpath marked as an orange line —, the latter emphasized as a thick pink line ▬). (b) Reflect the part of this path ▪▪▪ after the offending point ○ about the $y = x+1$ line. This reflected subpath (emphasized with a thick pink dashed line ▪▪▪), together with the other original part of the path —, creates a new path from $(0,0)$ to a new point $(n-1, n+1)$, ○.

First, as shown in Fig. S13(A), take a bad path and find the first premature point ○ where it has just naughtily crossed the diagonal $y = x$. This offending point ○ lies on the line $y = x+1$. Divide the bad path into two parts: as shown in Fig. S13(A), that subpath before this offending point, —, and that subpath after, ▬. Then, reflect the ▬ part of the path after the offending point about the $y = x+1$ diagonal that it is touching (by swapping every rightward move for an upward one): the outcome is visualized as a dashed pink line ▪▪▪ in Fig. S13(B). This new path has a different ultimate destination ○ than the original terminal point (n,n), because by virtue of having *crossed* the diagonal, the offending point shows an imbalance of rightward and upward moves. Specifically, since each path is $2n$ steps long (e.g. is even), and the unreflected — part of the path had one more upwards move than rightwards move (having arrived at the $y = x+1$ line), the reflected part of the path ▪▪▪ reaches the new destination $(n-1, n+1)$, ○. By taking this reflected second part of the path (together with the unreflected initial part of the path), a new path from $(0,0)$ to $(n-1, n+1)$ has been created. These reflected paths from $(0,0)$ to $(n-1, n+1)$ (as an unrestricted set) are one-to-one with the original bad paths from $(0,0)$ to (n,n) (since the reflection operation can be uniquely reversed); this bijection means that these sets of paths have the same count. There are $\binom{2n}{n-1}$ such unrestricted paths from $(0,0)$ to $(n-1, n+1)$, so the total number of inoffensive good paths is $\boxed{C_n = \binom{2n}{n} - \binom{2n}{n-1}}$.

Now, we adapt the essence of this tactic to confront our present problem (counting the 1D random walk paths that arrive at a net position m for the first time after exactly t steps). The gambit is the same as above, save for some key details.

bioRxiv preprint doi: https://doi.org/10.64898/2026.09.16.750450; this version posted September 17, 2026. The copyright holder for this preprint (which was not certified by peer review) is the author/funder, who has granted bioRxiv a license to display the preprint in perpetuity. It is made available under a CC-BY 4.0 International license.

Generalized counting of arbitrary first arrival paths. Counting the number of walk trajectories that terminate at a final position of $m = R(t) - L(t)$ in a total of $t = R(t) + L(t)$ steps, crucially *without* visiting m beforehand, shares the structure of the classic Catalan counting problem above, but with two key differences. First, the forbidden line that permissible trajectories must avoid is not the identity diagonal $R = L$ (e.g. $y = x$) line, but instead the $m = R - L$ line. Second, whereas the Catalan counting problem permitted touches to the diagonal line—and in particular identified all bad paths exactly by the property that they all crossed it—our setting demands we further exclude paths that even merely touch the $m = R - L$ line. Summarizing in other words, the Catalan problem counts all paths with $R > L$ as bad; our current problem (regarding the first passage of a random walk) counts all paths with $R \geq L + m$ as bad.

Accordingly, we change the procedure successful for the Catalan problem to this setting as follows. As before, we operate in the two-dimensional Cartesian lattice defined by the running number of rightward $R(t)$ and left $L(t)$ steps, where moves are depicted by right and upwards arrow moves in the $(R(t), L(t))$ lattice, respectively. Recall that in this lattice, descending diagonal lines of slope -1 together with y-intercept of $L(t) = t$ describe all endpoints of paths of total length t; conversely, ascending lines of slope $+1$ define the final positions $m = R(t) - L(t)$ of trajectories (as illustrated in Fig. S14). Thus, all valid trajectories of interest terminate at the intersection of these two lines $R(t) - L(t) = m$ and $R(t) + L(t) = t$, denoted by the black dot • in Fig. S14.

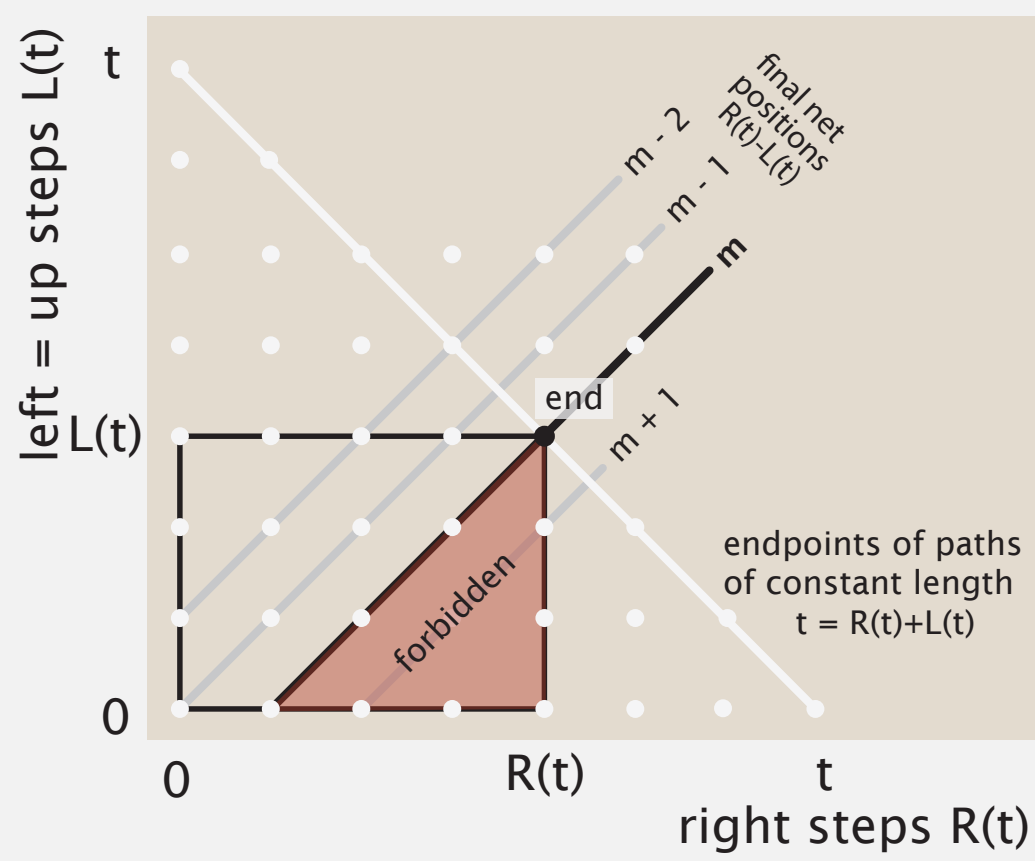

FIG. S14: Properties of the lattice of the running number of rightward steps $R(t)$ against the running number of left steps $L(t)$ (plotted as upwards in the lattice). Descending slope-one lines characterize trajectories of different total duration $t = R(t) + L(t)$, and ascending slope-one lines give the possible net endpoints $m = R(t) - L(t)$ of paths. (For example, by way of illustration, here all trajectories terminating at the black dot • endpoint satisfy $R(t) = 4$, $L(t) = 3$, $m = 1$, and $t = 7$.)

To accommodate the new requirements, we first recognize that all good paths terminating in such a net position m (let $m > 0$ without loss of generality) necessarily end in a rightwards move, $\rightarrow$, as shown in Fig. S15(A).[?] (Specifically, note that obviously there are possible paths ending at the endpoint using a leftwards move; but by necessity all of these paths will have already transgressed with respect to the forbidden $m = R - L$ line at some earlier point, as shown by Fig. S15(B).) Thus, instead of counting paths terminating at $(R(t), L(t))$, we specialize instead to counting those paths that terminate at the new endpoint of $(R(t) - 1, L(t))$ (shown as a magenta dot ○ in Fig. S15(C)) and then deterministically take the required final rightwards step. So, our count is actually confined to a smaller sublattice from the origin $(0,0)$ to the effective new endpoint $(R(t) - 1, L(t)) = \left(\frac{t+m}{2} - 1, \frac{t-m}{2}\right)$ (a region outlined as a magenta box in Fig. S15(C)). This leaves some remaining nontrivial bad paths to account for, as in Fig. S15(D).

bioRxiv preprint doi: https://doi.org/10.64898/2026.09.16.750450; this version posted September 17, 2026. The copyright holder for this preprint (which was not certified by peer review) is the author/funder, who has granted bioRxiv a license to display the preprint in perpetuity. It is made available under a CC-BY 4.0 International license.

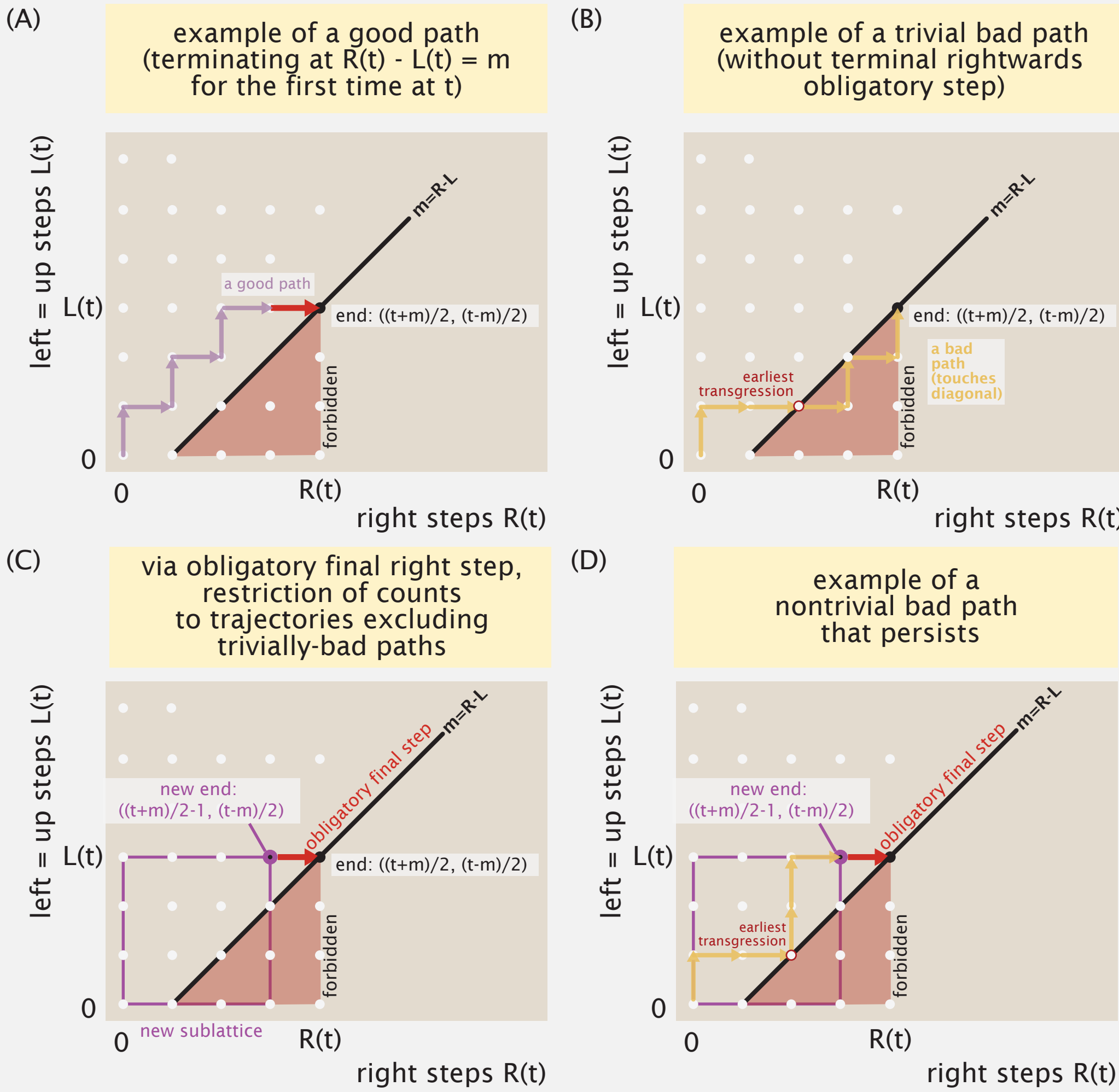

FIG. S15: Structure of valid paths. (A) Example of good path showing the required final rightwards steps. (B) Example of a trivially bad path, which we can anticipate is automatically problematic since it lacks the required final rightwards move. (C) Counting valid paths to the destination $(R(t), L(t)) = \left(\frac{t+m}{2}, \frac{t-m}{2}\right)$, denoted with the black dot •, can be narrowed to the count of valid paths to $(R(t)-1, L(t)) = \left(\frac{t+m}{2}-1, \frac{t-m}{2}\right)$, ○, that then commit to the only feasible final rightwards step, →. (D) Example of a nontrivial bad path that manifests this required final rightwards move, but still earlier transgresses.

Now our gambit is largely similar to the Catalan numbers, up to the change in the offending diagonal we care about (avoiding crossing). Specifically, the total number of all paths (good or bad) are those ending at the new endpoint $(R(t)-1, L(t)) = \left(\frac{t+m}{2}-1, \frac{t-m}{2}\right)$, ○, giving a total count $\binom{t-1}{\frac{t-m}{2}}$, again counting all choices of where rightward steps appear among all $t-1$ timesteps of the relevant trajectory.

We now count the nontrivial bad paths among these by establishing a bijection with easier-to-count paths, again by choosing a suitable reflection. As shown by the two examples in Fig. S16, first find the earliest point where a bad path offensively touches the relevant diagonal $R(t) - L(t) = m$ line. Consider all moves subsequent to this offending point (highlighted in thick pink ▬ in Fig. S16), and for every rightwards move swap out a leftwords move, and vice versa, to accomplish a local rotation about the diagonal. The resulting path, highlighted in dashed pink ▬ ▬ ▬ in Fig. S16, will always map the previous terminal point ○ at $\left(\frac{t+m}{2}-1, \frac{t-m}{2}\right)$ to a new reflected destination point $\left(\frac{t+m}{2}, \frac{t-m}{2}-1\right)$, denoted as ○ in Fig. S16. This new reflected destination does not vary over bad paths, as illustrated by Fig. S16. (This same destination point results because the former destination point $\left(\frac{t+m}{2}-1, \frac{t-m}{2}\right)$ definitionally has an extra leftwards move compared to all points on the diagonal $m = R - L$, so a reflection about this line decreases the number of leftwards moves and increases the number of rightwards moves by one.)

bioRxiv preprint doi: https://doi.org/10.64898/2026.09.16.750450; this version posted September 17, 2026. The copyright holder for this preprint (which was not certified by peer review) is the author/funder, who has granted bioRxiv a license to display the preprint in perpetuity. It is made available under a CC-BY 4.0 International license.

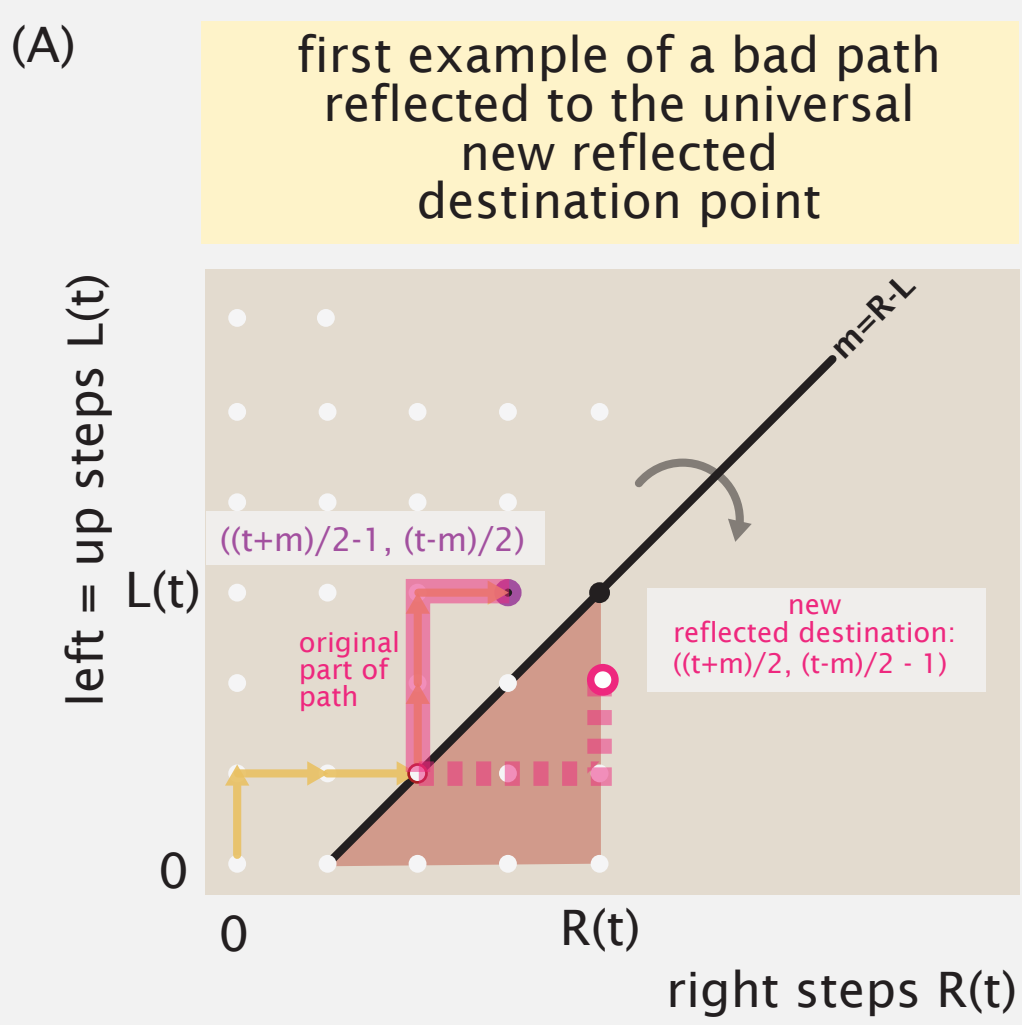

FIG. S16: Two examples of applying a reflection to map distinct bad paths to the same reflected destination point, enabling counting. Given an example of a bad path as in (A) or (B), find the first point offending point where the path intersects the diagonal line $m = R(t) - L(t)$ prematurely, and retain the path's moves before this offending point, denoted by an orange line —. Reflect the subsequent steps outlined in pink ▬ with respect to the diagonal, yielding the resulting reflected subpath given by the dashed pink line ▪▪▪. No matter where the original bad paths (and in particular where their earliest transgressions specifically occur), the resulting destination of the reflected subpath arrives at the same new reflected destination point, always located at $\left(\frac{t+m}{2}, \frac{t-m}{2} - 1\right)$, denoted as ○.

Since this reflection process is unique and reversible, we have found a correspondence the bad paths of interest to all lattice trajectories from the origin $(0,0)$ to the point $\left(\frac{t+m}{2}, \frac{t-m}{2} - 1\right)$. These lattice paths are still length $(t-1)$, and so there are $\binom{t-1}{\frac{t+m}{2}}$ bad paths.

In sum, we just established that the number of paths that arrive at position $m = R(t) - L(t)$ for the first time exactly at time t is

$$\#(m,t) = \binom{t-1}{\frac{t-m}{2}} - \binom{t-1}{\frac{t+m}{2}} \tag{S26}$$

$$= \boxed{\frac{m}{t}\binom{t}{\frac{t-m}{2}}}. \tag{S27}$$

Note some special cases of this eqn. S27:

- As noted early on, this relation is evaluated only for integer numbers of left or right steps, so we will regard this count as returning 0 when t and m differ in parity;
- $t = m$ returns $\#(m,t) = 1 \times \binom{t}{0} = 1$ path, as we intuitively require (the only way to get to position m in exactly t steps is to go there ballistically for t e.g. consecutive rightwards steps).

So for a random walk that steps right with probability p, the probability of first hitting m at t is just this degeneracy times the probability of taking the requisite number of rightwards steps,

$$\mathbb{P}(m,t) = \begin{cases} \frac{m}{t}\binom{t}{\frac{t-m}{2}} p^{(t+m)/2}(1-p)^{(t-m)/2} & \text{if } t \equiv m \mod 2, \\ 0 & \text{else.} \end{cases} \tag{S28}$$

or in the case of an unbiased random walk with $p = (1-p) = 1/2$,

$$\mathbb{P}(m,t) = \begin{cases} \frac{m}{t}\binom{t}{\frac{t-m}{2}} \frac{1}{2^t} & \text{if } t \equiv m \mod 2, \\ 0 & \text{else.} \end{cases} \tag{S29}$$

bioRxiv preprint doi: https://doi.org/10.64898/2026.09.16.750450; this version posted September 17, 2026. The copyright holder for this preprint (which was not certified by peer review) is the author/funder, who has granted bioRxiv a license to display the preprint in perpetuity. It is made available under a CC-BY 4.0 International license.

2. Interpretations and continuum approximations

To help interpret the first hitting probability specified by eqn. S28, we note that the total marginal probability of visiting site m at time t, regardless of whether it has been visited before, is just,

$$\mathbb{P}_{\text{marginal}}(m,t) = \binom{t}{\frac{t-m}{2}} p^{(t+m)/2}(1-p)^{(t-m)/2}. \tag{S30}$$

(This follows from simply counting the paths of length t with an excess of m rightwards steps, without any further requirements.) This expression reveals an interesting connection: the first-hitting probability eqn. S28 is m/t times the overall probability of the marginal probability,

$$\mathbb{P}(m,t) = \frac{m}{t}\mathbb{P}_{\text{marginal}}(m,t).$$

This says that of all the paths that successfully end at site m in time t, a fraction m/t of them do so for the first time.

The marginal distribution of a random walker's position $\mathbb{P}_{\text{marginal}}(m,t)$ famously approaches a Gaussian distribution in the small-timestep, large t continuum limit. Specifically, for the unbiased $p = 1/2$ case,

$$\mathbb{P}_{\text{marginal}}(m,t) \approx \frac{1}{\sqrt{2\pi t}} \exp\left[-\frac{m^2}{2t}\right]. \tag{S31}$$

This limit for the marginal distribution gives the analogous limit for the first-hitting distribution as,

$$\mathbb{P}(m,t) \approx \frac{m}{\sqrt{2\pi} t^{3/2}} \exp\left[-\frac{m^2}{2t}\right]. \tag{S32}$$

3. Survival probability

The probability that a walker has *not* hit position m by a time t is equal to the chance it has not first hit m at all preceding times t', namely these nasty expressions,

$$\mathbb{P}(m,>t) = 1 - \sum_{t'=m}^{t} \mathbb{P}(m,t') \tag{S33}$$

$$= 1 - \sum_{t'=m}^{t} \frac{m}{t'}\binom{t'}{\frac{t'-m}{2}}\frac{1}{2^{t'}} \qquad \text{(now reindex: define } 2\ell \equiv t-m \text{ as time exceeding the ballistic trajectory)} \tag{S34}$$

$$= 1 - \frac{m}{2^m}\left(\sum_{\ell=0}^{\ell=\frac{t-m}{2}} \frac{1}{m+2\ell}\frac{1}{2^{2\ell}}\binom{m+2\ell}{\ell}\right) \tag{S35}$$

$$= 1 - \frac{m}{2^m}\left(\frac{1}{m} + \frac{1}{m+2}\frac{1}{2^2}(m+2) + \frac{1}{m+4}\frac{1}{2^4}\frac{(m+4)!}{(m+2)!2!} + \cdots\right) \tag{S36}$$

$$= 1 - \frac{m}{2^m}\left(\frac{1}{m} + \frac{1}{2^2} + \cdots + \frac{1}{t-2}\frac{1}{2^{t-m-2}}\binom{t-2}{\frac{t-m}{2}} + \frac{1}{t}\frac{1}{2^{t-m}}\binom{t}{\frac{t-m}{2}}\right) \tag{S37}$$

These expressions smoothly capture the algebraic cases discussed in the main text, predicting the mean first passage time for the special cases of $r = m = 5$, and $r = 6 = m+1$. There we discussed that in both cases, the survival probability is $31/32 = 1-(1/2)^5$. We can also see this from this equation eqn. S37: starting from $t = m$, the summation only contains one term, $\ell = 0$, giving $P(m,>m) = 1 - \frac{1}{2^m}$. For the case $t = m+1$, the probability is unchanged: this follows since nonzero terms in the summation advance by increments of two (as required by matching parity of m and t), adding just one extra step does not change the survival probability, $\mathbb{P}(m,>t) = \mathbb{P}(m,>t+1)$.

C: Analytics of random walks with deterministic reset

Figure 10 of the main text compares the mean first passage times of random walks with different reset policies. Here, we present a brief exposition on the mean first passage time for a random walker subject to deterministic reset, which has previously been derived by Refs.[3,4].

bioRxiv preprint doi: https://doi.org/10.64898/2026.09.16.750450; this version posted September 17, 2026. The copyright holder for this preprint (which was not certified by peer review) is the author/funder, who has granted bioRxiv a license to display the preprint in perpetuity. It is made available under a CC-BY 4.0 International license.

Our general strategy is to first derive an analytical solution to the probability density $p(x,t)$ that the random walker will be at location x at time t. Then, the integral $\int_{-\infty}^{\text{target}} p(x,t)dx$ gives the survival probability, from which the mean first passage time can be calculated.

1. Probability distribution of random searcher at position x at time t

Let the random walker be on the one dimensional line, seeking a target located at $x = x_T$, periodically reset to the origin at every time period T_R. Between resets, the only dynamic at play is the diffusion of the random searcher, subject to an absorbing boundary condition at the target x_T. It is instructive to write the evolution of the probability distribution between resets iteratively. Consider then the time period $t = 0$ up to (but not including) $t = T_R$. The probability of a random searcher being at location x and time t under such a diffusion-absorption paradigm is given by

$$p_0(x,t) = \frac{1}{\sqrt{4\pi Dt}}\left(e^{-\frac{x^2}{4Dt}} - e^{\frac{-(x-2x_T)^2}{4Dt}}\right), \qquad 0 \le t < T_R, \tag{S38}$$

where the subscript 0 in $p_0(x,t)$ reminds us that the expression is valid before the first reset occurs.

At time $t = T_R$, the reset clock ticks. Any probability mass not absorbed at the target, given by $S(T_R) = \int_{-\infty}^{x_T} p_0(x,T_R)dx$, is reset back to the origin. The integral to obtain the survival probability evaluates to the closed form

$$S(T_R) = \int_{-\infty}^{x_T} p_0(x,T_R)dx = \text{erf}\left(\frac{x_T}{\sqrt{4DT_R}}\right), \tag{S39}$$

where $\text{erf}(\cdot)$ is the error function.

From the time interval $t = T_R$ upto (but not including) $t = 2T_R$, the random walker is once again subjected to the diffusion-absorption paradigm, and the probability distribution of the searcher is given by

$$p_1(x,t) = \underbrace{\text{erf}\left(\frac{x_T}{\sqrt{4DT_R}}\right)}_{\text{survival probability of searcher at } t=T_R} \underbrace{\frac{1}{\sqrt{4\pi D(t-T_R)}}\left(e^{-\frac{x^2}{4D(t-T_R)}} - e^{\frac{-(x-2x_T)^2}{4D(t-T_R)}}\right)}_{\text{diffusion-absorption during } T_R \le t < 2T_R}, \qquad T_R \le t < 2T_R, \tag{S40}$$

where the subscript 1 in $p_1(x,t)$ reminds us that the expression is valid only between the first and second resets. In the above expression, the survival probability $\text{erf}\left(\frac{x_T}{\sqrt{4DT_R}}\right)$ accounts for the probability that the random searcher did *not* find its target and was reset to the origin at $t = T_R$. The remaining terms account for the diffusion-absorption dynamics *after* the first reset.

At time $t = 2T_R$, the reset clock ticks once again. The probability mass that survived is given by

$$\begin{aligned} S(2T_R) &= \int_{-\infty}^{x_T} p_1(x,2T_R)dx = \text{erf}\left(\frac{x_T}{\sqrt{4DT_R}}\right)\int_{-\infty}^{x_T} \frac{1}{\sqrt{4\pi DT_R}}\left(e^{-\frac{x^2}{4DT_R}} - e^{\frac{-(x-2x_T)^2}{4DT_R}}\right)dx, \\ &= \text{erf}\left(\frac{x_T}{\sqrt{4DT_R}}\right)^2, \end{aligned} \tag{S41}$$

and once again, the random searcher that has survived thus far is subjected to the diffusion-absorption paradigm over the time period $t = 2T_R$ upto (but not including) $t = 3T_R$. The probability distribution over this time period is given by

$$p_2(x,t) = \underbrace{\text{erf}\left(\frac{x_T}{\sqrt{4DT_R}}\right)^2}_{\text{survival probability of searcher at } t=2T_R} \underbrace{\frac{1}{\sqrt{4\pi D(t-2T_R)}}\left(e^{-\frac{x^2}{4D(t-2T_R)}} - e^{\frac{-(x-2x_T)^2}{4D(t-2T_R)}}\right)}_{\text{diffusion-absorption during } 2T_R \le t < 3T_R}, \qquad 2T_R \le t < 3T_R. \tag{S42}$$

Writing the solutions over the zeroth, first, and second time periods one on top of another,

$$p_0(x,t) = \frac{1}{\sqrt{4\pi Dt}}\left(e^{-\frac{x^2}{4Dt}} - e^{\frac{-(x-2x_T)^2}{4Dt}}\right), \qquad 0 \le t < T_R, \tag{S43}$$

$$p_1(x,t) = \text{erf}\left(\frac{x_T}{\sqrt{4DT_R}}\right) \frac{1}{\sqrt{4\pi D(t-T_R)}}\left(e^{-\frac{x^2}{4D(t-T_R)}} - e^{\frac{-(x-2x_T)^2}{4D(t-T_R)}}\right), \qquad T_R \le t < 2T_R, \tag{S44}$$

$$p_2(x,t) = \text{erf}\left(\frac{x_T}{\sqrt{4DT_R}}\right)^2 \frac{1}{\sqrt{4\pi D(t-2T_R)}}\left(e^{-\frac{x^2}{4D(t-2T_R)}} - e^{\frac{-(x-2x_T)^2}{4D(t-2T_R)}}\right), \qquad 2T_R \le t < 3T_R, \tag{S45}$$

bioRxiv preprint doi: https://doi.org/10.64898/2026.09.16.750450; this version posted September 17, 2026. The copyright holder for this preprint (which was not certified by peer review) is the author/funder, who has granted bioRxiv a license to display the preprint in perpetuity. It is made available under a CC-BY 4.0 International license.

and so on, we see that the solution can be generally written as

$$p(x,t) = \mathrm{erf}\left(\frac{x_T}{\sqrt{4DT_R}}\right)^n p_0(x,t-nT_R)\,, \qquad nT_R \le t < (n+1)\,T_R. \tag{S46}$$

Figure S17 shows an example of the dynamics of the probability distribution of a random walker subject to deterministic reset.

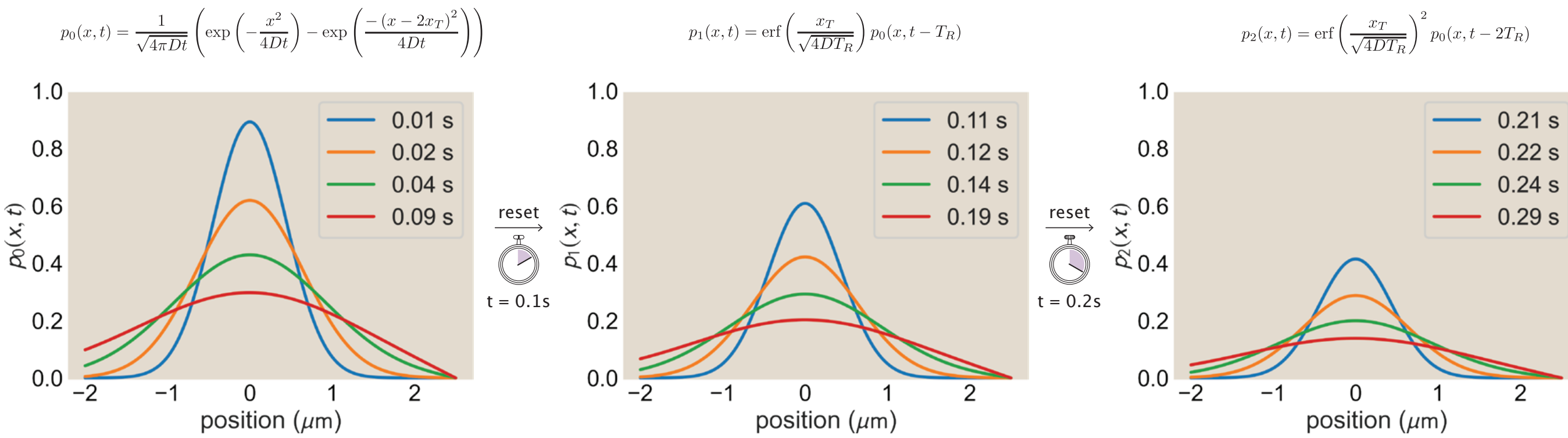

FIG. S17: Probability distribution $p(x,t)$ of random walker subject to deterministic reset for a diffusion coefficient $D = 10\,\mu\mathrm{m}^2$/s and reset time period $T_R = 0.1$ seconds, with the target placed $2.5\,\mu$m to the right of the origin.

2. Survival probability of random searcher

We are now interested in evaluating the survival probability $S(t)$ by integrating $p(x,t)$ from $x = -\infty$ to $x = x_T$,

$$S(t) = \int_{-\infty}^{x_T} p(x,t)dx\,, \tag{S47}$$

$$= \int_{-\infty}^{x_T} \left(\mathrm{erf}\left(\frac{x_T}{\sqrt{4DT_R}}\right)\right)^n p_0(x,t-nT_R)dx\,, \qquad nT_R \le t < (n+1)\,T_R\,. \tag{S48}$$

Pulling the error function outside of the integral,

$$S(t) = \left(\mathrm{erf}\left(\frac{x_T}{\sqrt{4DT_R}}\right)\right)^n \int_{-\infty}^{x_T} p_0(x,t-nT_R)dx\,, \qquad nT_R \le t < (n+1)\,T_R\,. \tag{S49}$$

Using eqn. S39, the survival probability evaluates to

$$S(t) = \left(\mathrm{erf}\left(\frac{x_T}{\sqrt{4DT_R}}\right)\right)^n \mathrm{erf}\left(\frac{x_T}{\sqrt{4D\,(t-nT_R)}}\right)\,, \qquad nT_R \le t < (n+1)\,T_R\,. \tag{S50}$$

That is, the survival probability at any time t is the survival probability at time $t = nT_R$ (after n resets) multiplied by the survival probability after duration $t - nT_R$.

Figure S18 depicts the survival probability for the dynamics shown in Figure S17.

3. Mean first passage time of random searcher

The evaluation of the mean first passage time requires some careful algebra. The probability of hitting the target $f(t)$ at time t is related to the survival probability as

$$f(t) = \frac{d(1-S)}{dt} = -\frac{dS}{dt}\,. \tag{S51}$$

Then, the mean first passage time is given by

$$T = \mathbb{E}[t] = \int_0^\infty t f(t)dt\,. \tag{S52}$$

bioRxiv preprint doi: https://doi.org/10.64898/2026.09.16.750450; this version posted September 17, 2026. The copyright holder for this preprint (which was not certified by peer review) is the author/funder, who has granted bioRxiv a license to display the preprint in perpetuity. It is made available under a CC-BY 4.0 International license.

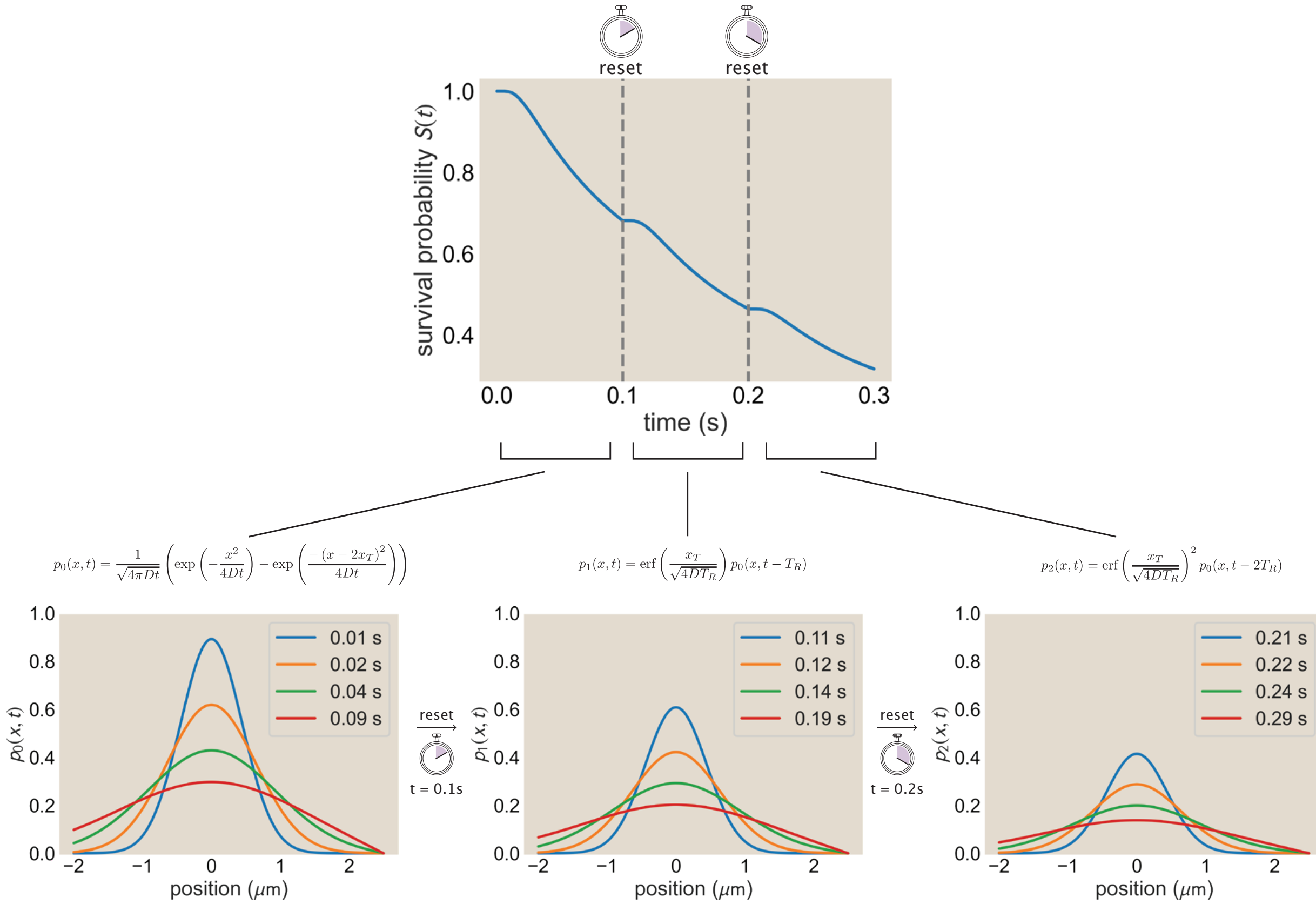

FIG. S18: Survival probability and probability distribution of a random walker subject to deterministic reset for a diffusion coefficient $D = 10\,\mu\text{m}^2/\text{s}$ and reset time period $T_R = 0.1$ seconds, with the target placed $2.5\,\mu\text{m}$ to the right of the origin.

Substituting the expression for $f(t)$ from eqn. S51,

$$T = -\int_0^\infty t\frac{dS}{dt}dt\,. \tag{S53}$$

Performing integration by parts, we get

$$T = -\left[tS - \int Sdt\right]_0^\infty = \int_0^\infty Sdt\,, \tag{S54}$$

where we have used the fact that eventually the survival probability is zero for $t \to \infty$. The above equation directly relates the survival probability to the mean first passage time.

Substituting the expression for the survival probability for random walks with deterministic reset in eqn S50,

$$T = \sum_{n=0}^{\infty} \int_{nT_R}^{(n+1)T_R} \left(\text{erf}\left(\frac{x_T}{\sqrt{4DT_R}}\right)\right)^n \text{erf}\left(\frac{x_T}{\sqrt{4D(t-nT_R)}}\right) dt. \tag{S55}$$

Splitting up the integral over the time periods between resets, we get

$$T = \int_0^{T_R} \text{erf}\left(\frac{x_T}{\sqrt{4Dt}}\right) dt + \int_{T_R}^{2T_R} \text{erf}\left(\frac{x_T}{\sqrt{4DT_R}}\right) \text{erf}\left(\frac{x_T}{\sqrt{4D\,(t-T_R)}}\right) dt + \int_{2T_R}^{3T_R} \left(\text{erf}\left(\frac{x_T}{\sqrt{4DT_R}}\right)\right)^2 \text{erf}\left(\frac{x_T}{\sqrt{4D\,(t-2T_R)}}\right) dt + \ldots. \tag{S56}$$

Applying the transformation $\tau = t - nT_R$ for the nth integral, where $n = 0, 1, 2\ldots$,

$$T = \int_0^{T_R} \text{erf}\left(\frac{x_T}{\sqrt{4D\tau}}\right) d\tau + \int_0^{T_R} \text{erf}\left(\frac{x_T}{\sqrt{4DT_R}}\right) \text{erf}\left(\frac{x_T}{\sqrt{4D\tau}}\right) d\tau + \int_0^{T_R} \left(\text{erf}\left(\frac{x_T}{\sqrt{4DT_R}}\right)\right)^2 \text{erf}\left(\frac{x_T}{\sqrt{4D\tau}}\right) d\tau + \ldots. \tag{S57}$$

bioRxiv preprint doi: https://doi.org/10.64898/2026.09.16.750450; this version posted September 17, 2026. The copyright holder for this preprint (which was not certified by peer review) is the author/funder, who has granted bioRxiv a license to display the preprint in perpetuity. It is made available under a CC-BY 4.0 International license.

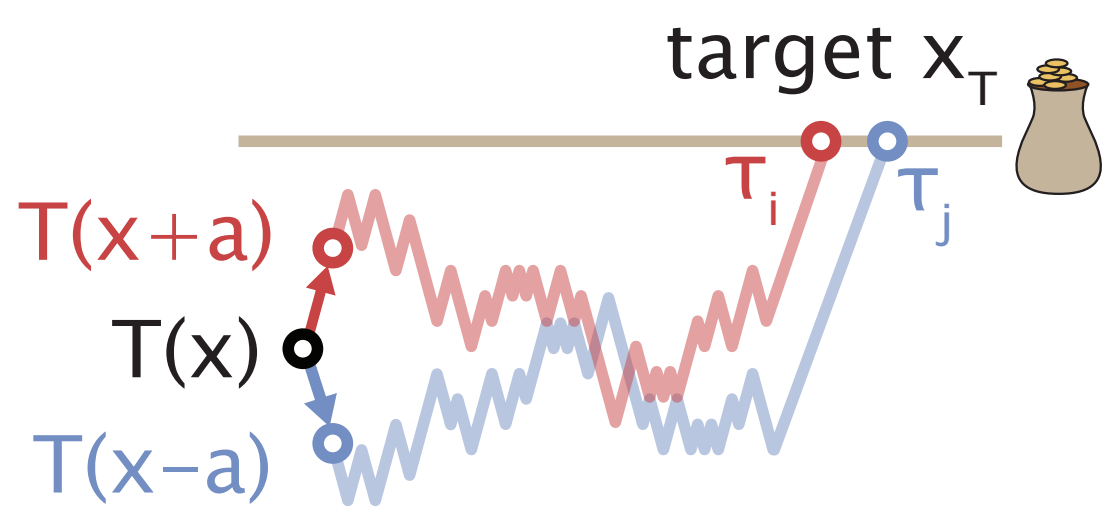

FIG. S19: One way to view the recursion relations for mean first passage time (for instance eqn. 22)—which are also called Kolmogorov backwards equations—is in terms of explicit averages over trajectories.

The integral can be factorized into an infinite sum of a geometric series multiplied by the integral of the error function over the time period between resets,

$$T = \left(1 + \text{erf}\left(\frac{x_T}{\sqrt{4DT_R}}\right) + \left(\text{erf}\left(\frac{x_T}{\sqrt{4DT_R}}\right)\right)^2 \dots\right) \int_0^{T_R} \text{erf}\left(\frac{x_T}{\sqrt{4D\tau}}\right) d\tau\,. \tag{S58}$$

For a geometric series $1, r, r^2, r^3, \dots$, the infinite sum is given by $1/(1-r)$, where the multiplicative factor r satisfies the condition $r < 1$. Here, the multiplicative factor is $r = \text{erf}\left(\frac{x_T}{\sqrt{4DT_R}}\right)$, whose value is strictly less than 1. Thus, the mean first passage time becomes

$$T = \frac{1}{1 - \text{erf}\left(\frac{x_T}{\sqrt{4DT_R}}\right)} \int_0^{T_R} \text{erf}\left(\frac{x_T}{\sqrt{4D\tau}}\right) d\tau\,. \tag{S59}$$

It turns out that the fraction $1/\left(1 - \text{erf}\left(\frac{x_T}{\sqrt{4DT_R}}\right)\right)$ measures the mean number of attempts taken to reach the target. The integral $\int_0^{T_R} \text{erf}\left(\frac{x_T}{\sqrt{4D\tau}}\right) d\tau$ can be interpreted as the mean first passage time within the time interval between resets. That is to say, the mean first passage time is schematically given by

$$T \quad = \quad \text{average number of attempts} \quad \times \quad \text{average time to reach target between two resets}\,. \tag{S60}$$

The reader may recognize the parallels between the above expression and eqn. 8 of the main text. This is because random walks with deterministic resets can be approached via the language of the geometric distribution, which is explored further in Appendix F 2.

In fact, the structure of eqn. S60 can be generalized using Wald's identity, briefly explained below.

Wald's Identity[5]. Consider a sequence of independent and identically drawn real-valued random variables, $(X_1, X_2, X_3, \dots)$, with a common expectation $\mathbb{E}[X]$. Let N be an integer random variable with finite mean, such that an event occurring at discrete time $N = n$ is determined entirely by the history of the sequence, $(X_1, X_2, X_3, \dots X_n)$, and does not depend on future values of the sequence, $X_{n+1}, X_{n+2}, \dots$ and so forth. Such a random variable N is called a stopping time.

Then, Wald's identity states

$$\mathbb{E}\left[X_1 + X_2 + X_3 + \dots X_N\right] = \mathbb{E}\left[N\right] \mathbb{E}\left[X\right]\,. \tag{S61}$$

Note that Wald's identity generalizes eqn. 8 to stopping criteria that may depend on the entire history of the sequence. Overlaying Wald's identity on the mean first passage time for random walks with deterministic reset,

$$T = \underbrace{\frac{1}{1 - \text{erf}\left(x_T/\sqrt{4DT_R}\right)}}_{\mathbb{E}[N]} \underbrace{\int_0^{T_R} \text{erf}\left(\frac{x_T}{\sqrt{4D\tau}}\right) d\tau}_{\mathbb{E}[\text{duration of one attempt}]}\,. \tag{S62}$$

D: Average hitting times as explicit averages over trajectories

Here we briefly explain another vantage on the why the mean first hitting time for a target $T(x)$ starting at position x obeys a recursion relation or local average (Kolmogorov's backwards equations) with those of its neighbors, which explicitly centers

bioRxiv preprint doi: https://doi.org/10.64898/2026.09.16.750450; this version posted September 17, 2026. The copyright holder for this preprint (which was not certified by peer review) is the author/funder, who has granted bioRxiv a license to display the preprint in perpetuity. It is made available under a CC-BY 4.0 International license.

trajectories. Imagine that a set of N random walks are performed starting at position x until they hit the target. Call τ_i the time that the ith trajectory finally reaches the target. Then the empirical mean first hitting time of these is just the average of these N hitting time values,

$$T(x) \approx \frac{1}{N}\sum_{i=1}^{N}\tau_i. \tag{S63}$$

Consider the first step of each of these walks. As we are reminded by Fig. S19, either the trajectory stepped to $x+a$ or else the trajectory stepped to $x-a$. This bins all the trajectories into these two classes, which means we may split the above average sum into two parts,

$$T(x) \approx \frac{1}{N}\left(\sum_{i:\text{ step to }x+a}^{N}\tau_i + \sum_{j:\text{ step to }x-a}^{N}\tau_j\right), \tag{S64}$$

If N_+ of these trajectories first stepped to $x+a$, and N_- stepped to $x-a$, then we may further rewrite the average as,

$$T(x) \approx \frac{N_+}{N}\left(\frac{1}{N_+}\sum_{i:\text{ step to }x+a}^{N}\tau_i\right) + \frac{N_-}{N}\left(\frac{1}{N_-}\sum_{j:\text{ step to }x-a}^{N}\tau_j\right). \tag{S65}$$

Notice that $\frac{N_+}{N}$ represents the realized probability of stepping to $x+a$, and analogously $\frac{N_-}{N}$ represents the probability of stepping to $x-a$. Further, $\left(\frac{1}{N_+}\sum_{i:\text{ step to }x+a}^{N}\tau_i\right)$ represents the average arrival time of the trajectory conditioned on it first stepping to $x+a$, which we may identify as $T(x+a)$, and analogously $\left(\frac{1}{N_-}\sum_{j:\text{ step to }x-a}^{N}\tau_j\right)$ as $T(x-a)$. For a symmetric random walk where stepping left or right has equal probability, this allows us to rewrite the average further as,

$$T(x) \approx \frac{1}{2k} + \frac{1}{2}T(x+a) + \frac{1}{2}T(x-a), \tag{S66}$$

where we acknowledged the time required for the first step. Over many trajectories $N \to \infty$, this perspective recovers the eqn. 22 of the main text reasoned about directly from conditional probability.

E: Resets confine the search of the random walker

When is a random walk with reset equivalent to a random walk with confinement? The answer depends on our notion of "equivalence". In Ref.[10], the authors show this equivalence in the sense that the steady-state distribution of a diffusing particle with Poissonian reset is equivalent to that of a diffusing particle subject to a confining potential *without* reset, even though their steady-state probability currents are not equivalent. The steady-state distribution of a diffusing particle subject to a confining potential is equilibrium in nature, while that of a diffusing particle subject to Poissonian reset is non-equilibrium in nature.

In Appendix § E 1, we discuss equivalence of resets to confinement in the sense that it places an upper bound on the spread of the random walker's probability distribution. In Appendix § E 2, we discuss equivalence of resets to confinement in the sense that the asymptotic mean first passage time of a random walker with deterministic reset approaches that of a random walker under confinement, with the reflecting boundary being appropriately placed. Interestingly, for the same condition, the dynamics of the probability distributions for deterministic reset approaches that of confined diffusion.

1. Resets place an upper bound on the spread of the distribution

In Appendix § C 1, we showed that the probability distribution of a random walker subjected to periodic reset is prescribed as

$$p(x,t) = \text{erf}\left(\frac{x_T}{\sqrt{4DT_R}}\right)^n p_0(x, t-nT_R), \qquad nT_R \le t < (n+1)\,T_R, \tag{S67}$$

where n is the number of resets that occurred by time t, and

$$p_0(x, t-nT_R) = \frac{1}{\sqrt{4\pi D\,(t-nT_R)}}\left(e^{-\frac{x^2}{4D(t-nT_R)}} - e^{\frac{-(x-2x_T)^2}{4D(t-nT_R)}}\right), \qquad nT_R \le t < (n+1)\,T_R, \tag{S68}$$

bioRxiv preprint doi: https://doi.org/10.64898/2026.09.16.750450; this version posted September 17, 2026. The copyright holder for this preprint (which was not certified by peer review) is the author/funder, who has granted bioRxiv a license to display the preprint in perpetuity. It is made available under a CC-BY 4.0 International license.

describes the probability distribution of a random walker subject to an absorbing boundary condition at $x = x_T$ after the nth reset occurs. Let us shift the time frame by $\tau = t - nT_R$, so that at $t = nT_R$, $\tau = 0$. The solution becomes

$$p(x,t) = \text{erf}\left(\frac{x_T}{\sqrt{4DT_R}}\right)^n \frac{1}{\sqrt{4\pi D\tau}}\left(e^{-\frac{x^2}{4D\tau}} - e^{\frac{-(x-2x_T)^2}{4D\tau}}\right) \qquad 0 \le \tau < T_R, \tag{S69}$$

where n still tracks the number of resets that have occurred by time $\tau = t - nT_R$. The error function $\text{erf}\left(\frac{x_T}{\sqrt{4DT_R}}\right)^n$ quantifies the probability mass that survived up to the nth reset and does not contribute to any spatial variation. Factoring out the surviving probability mass by dividing both sides of the previous equation by $S(nT_R) = \text{erf}\left(\frac{x_T}{\sqrt{4DT_R}}\right)^n$,

$$\frac{p(x,nT_R+\tau)}{S(nT_R)} = \frac{1}{\sqrt{4\pi D\tau}}\left(e^{-\frac{x^2}{4D\tau}} - e^{\frac{-(x-2x_T)^2}{4D\tau}}\right) \qquad 0 \le \tau < T_R, \tag{S70}$$

we are left only with the dynamics of a random walker subject to an absorbing boundary condition at $x = x_T$ within the time period of a reset. Since the reset clock ticks at $\tau = T_R$, the spread of the above distribution is capped at $\sqrt{DT_R}$.

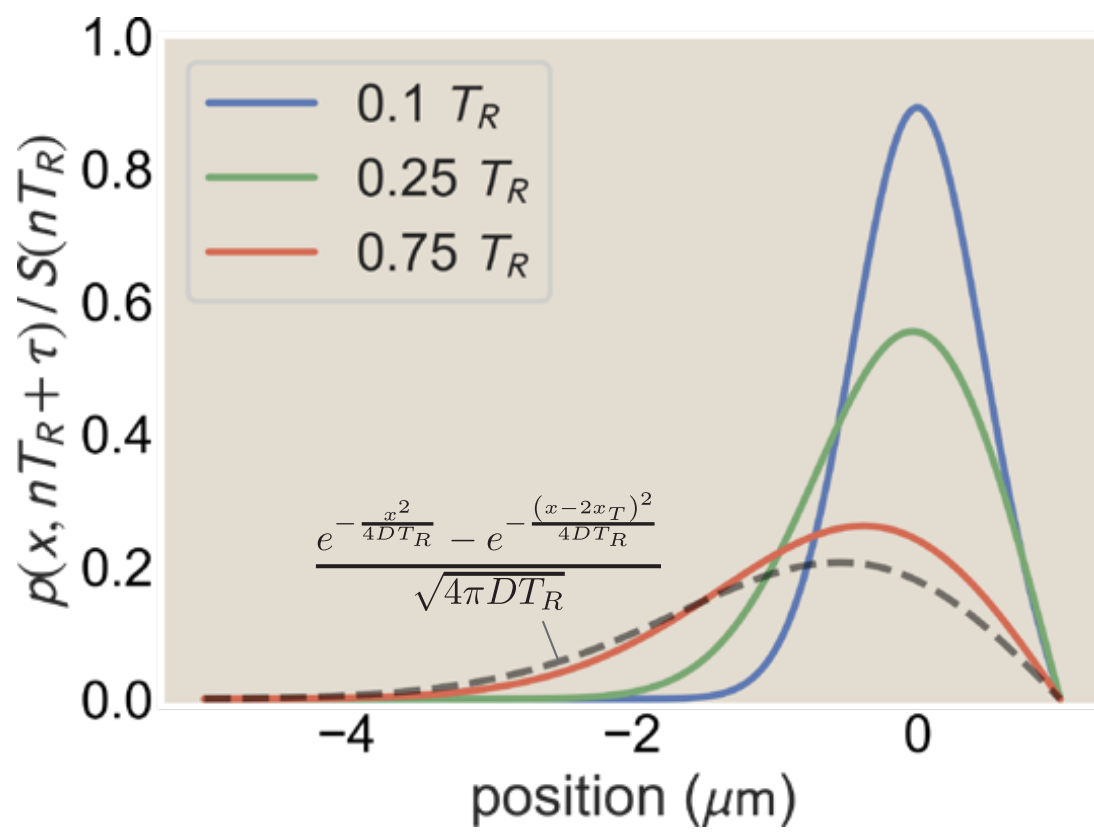

FIG. S20: The probability distribution spread of a random walker between two resets is strictly lower than that of a random walker without reset at $t = T_R$. Here, diffusion coefficient $D = 10\,\mu\text{m}^2$/s and reset time period $T_R = 0.1$ seconds, with the target placed $1\,\mu$m to the right of the origin.

2. Equivalence of asymptotics of mean first passage times

Thus far, we have discussed how resets endow an upper bound to the probability distribution spread of the random walker between any two resets. Under stronger conditions, before the first reset, the dynamics of a random walker with deterministic reset are seemingly equivalent to that of a confined walker without reset for all time. Intuitively, this is true if the resetting random walker (without confinement) speedily finds its target both before the first reset time T_R and before the confined random walker (without reset) interacts with the boundary L_R.

Let us quantify the above intuition. The mean first passage time for a random walker subject to reset was derived in Appendix C 3,

$$T = \frac{1}{1-\text{erf}\left(\frac{x_T}{\sqrt{4DT_R}}\right)} \int_0^{T_R} \text{erf}\left(\frac{x_T}{\sqrt{4D\tau}}\right) d\tau\,. \tag{S71}$$

bioRxiv preprint doi: https://doi.org/10.64898/2026.09.16.750450; this version posted September 17, 2026. The copyright holder for this preprint (which was not certified by peer review) is the author/funder, who has granted bioRxiv a license to display the preprint in perpetuity. It is made available under a CC-BY 4.0 International license.

The integral is of the form $\int_0^T \mathrm{erf}\left(\sqrt{b/t}\right) dt$. With the change of variable $z = \sqrt{b/t}$, the integral becomes

$$\int_0^T \mathrm{erf}\left(\sqrt{b/t}\right) dt = 2b \int_{\sqrt{b/T}}^{\infty} \mathrm{erf}(z)/z^3 dz, \tag{S72}$$

$$= 2b\left(\frac{\mathrm{erf}(z)}{2z^2} + \frac{1}{\sqrt{\pi}}\int_{\sqrt{b/T}}^{\infty} \frac{e^{-z^2}}{z^2} dz\right), \tag{S73}$$

$$= 2b\left(\frac{\mathrm{erf}(z)}{2z^2} + \frac{e^{-b/T}}{\sqrt{\pi}\sqrt{b/T}} - 1 + \mathrm{erf}\left(\sqrt{b/T}\right)\right), \tag{S74}$$

$$= T\left(\left(2\frac{b}{T}+1\right)\mathrm{erf}\left(\sqrt{\frac{b}{T}}\right) + 2\sqrt{\frac{b}{\pi T}}e^{-b/T} - 2\frac{b}{T}\right), \tag{S75}$$

where eqn. S73 arises from eqn. 14 of the tabulation[19] (with $n = 3$ and $a = 1$), eqn. S73 is obtained after calculating the integral, and eqn. S75 is obtained after a rearrangement of terms. Using S75, the mean first passage time becomes

$$T = \frac{T_R\left(\left(1+2\left(\frac{x_T}{2\sqrt{DT_R}}\right)^2\right)\mathrm{erf}\left(\frac{x_T}{2\sqrt{DT_R}}\right) + \frac{2}{\sqrt{\pi}}\left(\frac{x_T}{2\sqrt{DT_R}}\right)\exp\left(-\left(\frac{x_T}{2\sqrt{DT_R}}\right)^2\right) - 2\left(\frac{x_T}{2\sqrt{DT_R}}\right)^2\right)}{1-\mathrm{erf}\left(\frac{x_T}{2\sqrt{DT_R}}\right)}, \tag{S76}$$

In the limit of infinite reset time $T_R \to \infty$, for fixed target location x_T, the fraction $x_T/\sqrt{DT_R}$ is much smaller than 1. In this limit, we have the approximations $\mathrm{erf}\left(x_T/2\sqrt{DT_R}\right) \sim \frac{2x_T/2\sqrt{DT_R}}{\sqrt{\pi}}$, $1-\mathrm{erf}\left(x_T/2\sqrt{DT_R}\right) \sim 1$, $\left(x_T/2\sqrt{DT_R}\right)^2 \sim 0$, and $\exp\left(-\left(x_T/2\sqrt{DT_R}\right)^2\right) \sim 1$. The mean first passage time becomes

$$T \sim \frac{2}{\sqrt{\pi}} x_T \sqrt{\frac{T_R}{D}}. \tag{S77}$$

Compare the above to eqn 36 of Section III B, the mean first passage time for a random walker confined between the boundaries $x = -L_R$ and $x = x_T$ without any reset,

$$T_{\text{confined}}(0) \sim \frac{x_T L_R}{D} = \frac{2}{\sqrt{\pi}} x_T \sqrt{\frac{T_R}{D}} \text{ under confinement without reset}, \qquad T \sim \frac{2}{\sqrt{\pi}} x_T \sqrt{\frac{T_R}{D}} \text{ under periodic reset}, \tag{S78}$$

where the reflecting boundary was placed at $L_R = \frac{2}{\sqrt{\pi}}\sqrt{DT_R}$ in eqn 39 of Section III B following arguments of extremal value statistics.

Thus, the mean first passage time under reset scales similarly to that of a confined random walker, provided the target is much closer to the initial condition than the lengthscale L_R.

We see similar agreement of the dynamics of the probability distributions in Figure S21. Here, the probability distribution for a random walker under confinement was approximated by the solution

$$p_{\text{confined}}(x,t) = \frac{2}{L_R\left(1+\frac{x_T}{L_R}\right)} \sum_{n=0}^{\infty} \cos\left(\frac{(2n+1)\pi}{2}\frac{1+\frac{x}{L_R}}{1+\frac{x_T}{L_R}}\right)\cos\left(\frac{(2n+1)\pi}{2}\frac{1}{1+\frac{x_T}{L_R}}\right)\exp\left(-\frac{D}{L_R^2}\left(\frac{(2n+1)\pi}{2\left(1+\frac{x_T}{L_R}\right)}\right)^2 t\right), \tag{S79}$$

which is the solution to the diffusion equation with the reflecting boundary condition $\frac{\partial}{\partial} p\Big|_{x=-L_R} = 0$ and the absorbing boundary condition $p(x_T,t) = 0$ in the absence of reset. The plot corresponds to simulations of 500 modes.

bioRxiv preprint doi: https://doi.org/10.64898/2026.09.16.750450; this version posted September 17, 2026. The copyright holder for this preprint (which was not certified by peer review) is the author/funder, who has granted bioRxiv a license to display the preprint in perpetuity. It is made available under a CC-BY 4.0 International license.

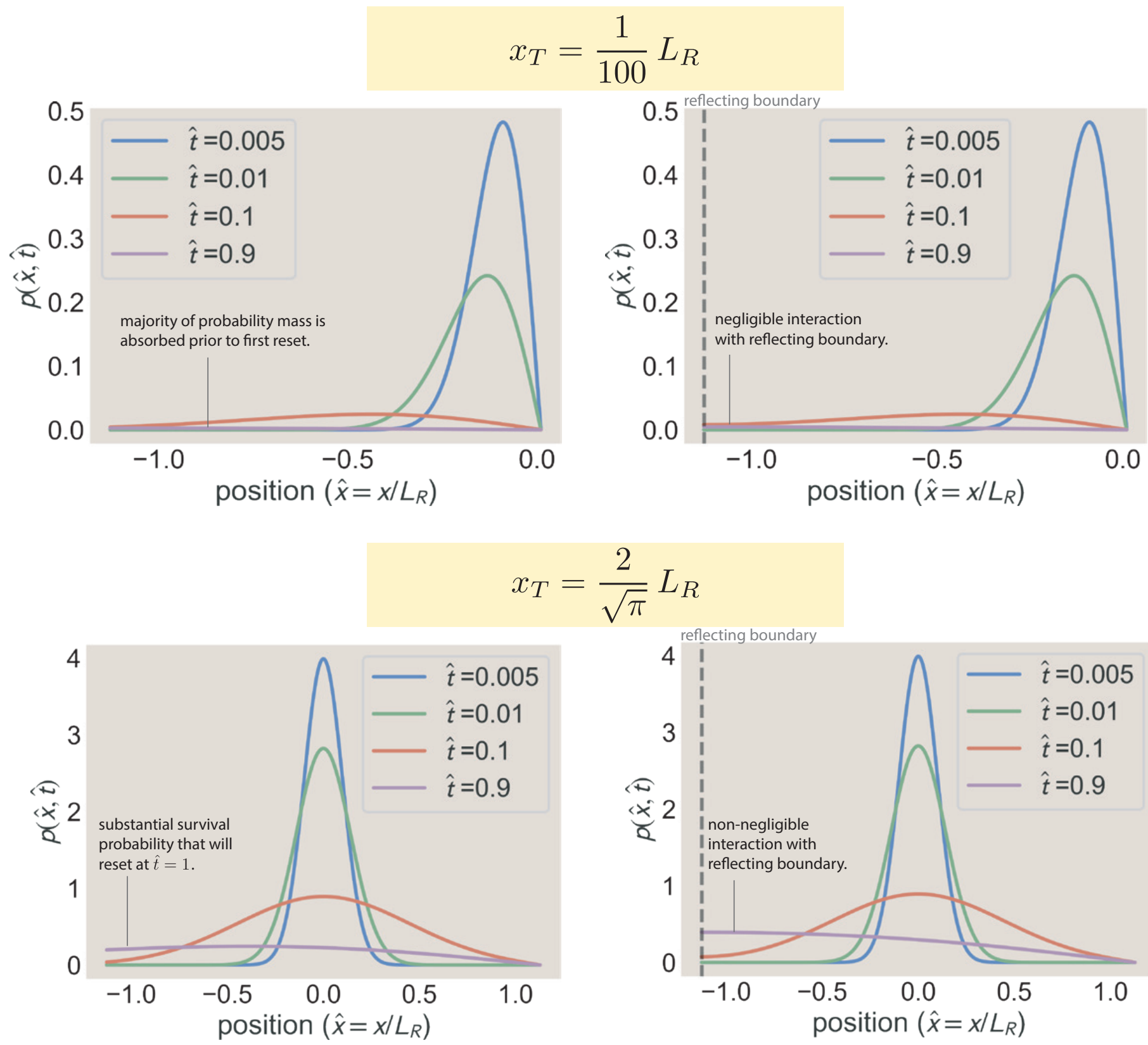

FIG. S21: The probability distribution of a random walker prior to the first reset (left column) approaches that of a random walker with a reflecting boundary at $x = -L_R$ (right column), provided $x_T \ll L_R$, where $L_R = \frac{2}{\sqrt{\pi}}\sqrt{DT_R}$ is the effective confinement length. When $x_T = 0.01L_R$, we see that the majority of the probability mass is absorbed at the target $x = x_T$ before the reset time T_R *and* before the probability mass substantially diffuses to the reflecting boundary. When $x_T = \pi L_R$, a non-negligible probability mass will be reset at time T_R, thus significantly deviating from the dynamics of diffusion without reset. Here, the diffusion coefficient is $D = 10\,\mu\text{m}^2/\text{s}$, the reset time period is $T_R = 0.1$ seconds, and time has been normalized using $\hat{t} = \frac{t}{T_R}$.

F: Additional results for random walks with resetting

1. Additional sweep of behavior of random walk under resets from simulation

In the main text, exploring how random walks with resets operate, we dissected the example of a particular target location $x_T = 5$ using simulations that prompted theoretical understanding. Here we briefly display the joint landscape of how both target locations and reset rates determine the mean time to successfully reach targets.

bioRxiv preprint doi: https://doi.org/10.64898/2026.09.16.750450; this version posted September 17, 2026. The copyright holder for this preprint (which was not certified by peer review) is the author/funder, who has granted bioRxiv a license to display the preprint in perpetuity. It is made available under a CC-BY 4.0 International license.

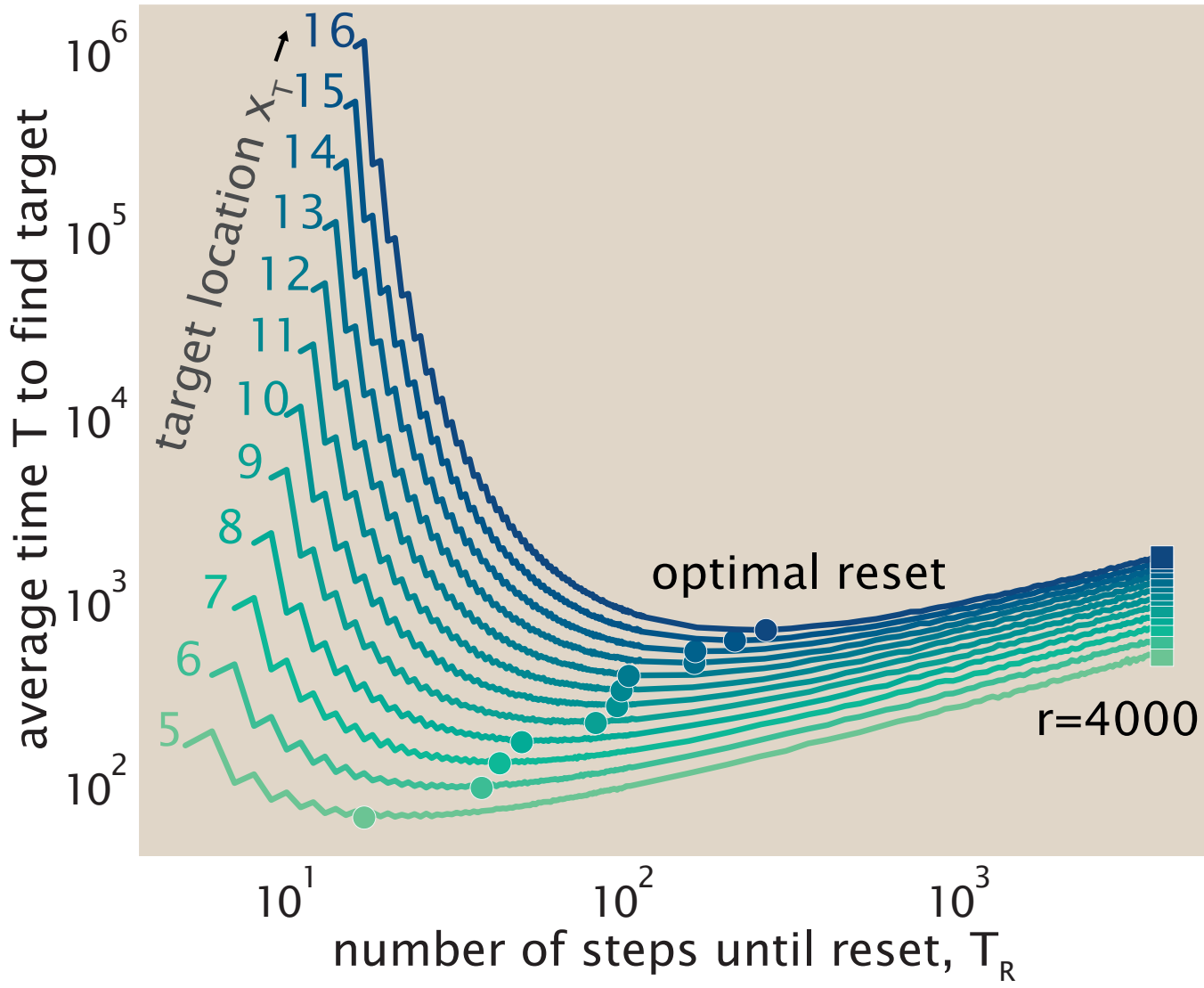

FIG. S22: Average time for seekers to reach a target, subject to different deterministic reset periods T_R (on the x-axis) and different target locations x_T (shown as lines with different hues of sea green to dark blue), using numerical simulations.

2. The geometric distribution as a language of exploration with memoryless resets

In the work of Kondev *et al.*[13], the first-passage problem with resetting is interpreted in terms of a geometric distribution. When resetting returns the system to the same initial state, any trajectory leading to success can be decomposed into a sequence of independent attempts, consisting of several failed attempts followed by a final successful one.

We denote by T_0 the first-passage time to the target in the absence of resetting, with probability density $f_0(t)$. We denote by R the time of the first reset event after the beginning of an attempt. Since resets occur as a Poisson process with constant rate r, R is exponentially distributed,

$$p_R(t) = re^{-rt}, \tag{S80}$$

and the probability that no reset has occurred by time t is

$$\mathbb{P}(R > t) = e^{-rt}. \tag{S81}$$

An attempt is successful when the target is reached before the next reset. We therefore define the success probability

$$q = \mathbb{P}(T_0 < R). \tag{S82}$$

Conditioning on the value of the no-reset first-passage time gives

$$q = \int_0^\infty \mathbb{P}(T_0 < R \mid T_0 = t)\, f_0(t)\, dt \tag{S83}$$

$$= \int_0^\infty \mathbb{P}(R > t)\, f_0(t)\, dt \tag{S84}$$

$$= \int_0^\infty e^{-rt} f_0(t)\, dt. \tag{S85}$$

Thus,

$$q = \mathbb{E}\left[e^{-rT_0}\right], \tag{S86}$$

so that the probability of success during a single reset-delimited attempt is simply the Laplace transform of the no-reset first-passage-time distribution evaluated at the reset rate r.

We next characterize the durations of successful and failed attempts. The duration of a successful attempt is

$$T_s = T_0 \,|\, T_0 < R, \tag{S87}$$

bioRxiv preprint doi: https://doi.org/10.64898/2026.09.16.750450; this version posted September 17, 2026. The copyright holder for this preprint (which was not certified by peer review) is the author/funder, who has granted bioRxiv a license to display the preprint in perpetuity. It is made available under a CC-BY 4.0 International license.

whereas the duration of a failed attempt is

$$T_f = R \,|\, R < T_0. \tag{S88}$$

For the successful attempts, Bayes' rule gives

$$\mathbb{E}[T_s] = \int_0^\infty t\,\mathbb{P}(T_0 = t \mid T_0 < R)\,dt \tag{S89}$$

$$= \frac{\int_0^\infty t\,\mathbb{P}(T_0 < R \mid T_0 = t)\,f_0(t)\,dt}{\mathbb{P}(T_0 < R)}. \tag{S90}$$

Using $\mathbb{P}(R > t) = e^{-rt}$, we obtain

$$\mathbb{E}[T_s] = \frac{\mathbb{E}\left[T_0 e^{-rT_0}\right]}{q}. \tag{S91}$$

We proceed similarly for the duration of a failed attempt. By definition,

$$\mathbb{E}[T_f] = \int_0^\infty t\,\mathbb{P}(R = t \mid R < T_0)\,dt \tag{S92}$$

$$= \frac{\int_0^\infty t\,re^{-rt}\,\mathbb{P}(T_0 > t)\,dt}{\mathbb{P}(R < T_0)}. \tag{S93}$$

Since the two possible outcomes of an attempt are success or failure,

$$\mathbb{P}(R < T_0) = 1 - q. \tag{S94}$$

Integrating the numerator by parts gives

$$\mathbb{E}[T_f] = \frac{1}{r} - \frac{\mathbb{E}\left[T_0 e^{-rT_0}\right]}{1-q}. \tag{S95}$$

Each attempt succeeds independently with probability q. Therefore, the number N_f of failed attempts before the first successful attempt is a geometric random variable,

$$\mathbb{P}(N_f = n) = (1-q)^n q, \qquad n = 0, 1, 2, \ldots, \tag{S96}$$

with mean

$$\mathbb{E}[N_f] = \frac{1-q}{q}. \tag{S97}$$

The total first-passage time is the sum of the durations of all failed attempts and the duration of the final successful attempt. Its mean is therefore

$$\langle T \rangle = \mathbb{E}[N_f]\,\mathbb{E}[T_f] + \mathbb{E}[T_s]. \tag{S98}$$

Substituting eqns. (S91) and (S95), we obtain

$$\langle T \rangle = \frac{1-q}{q}\left[\frac{1}{r} - \frac{\mathbb{E}\left[T_0 e^{-rT_0}\right]}{1-q}\right] + \frac{\mathbb{E}\left[T_0 e^{-rT_0}\right]}{q} \tag{S99}$$

$$= \frac{1-q}{rq}. \tag{S100}$$

Thus, a first-passage process with memoryless resetting can be coarse-grained into a sequence of independent attempts governed by a geometric distribution. All of the microscopic first-passage dynamics enter this description through the single-attempt success probability

$$q = \mathbb{E}\left[e^{-rT_0}\right]. \tag{S101}$$

bioRxiv preprint doi: https://doi.org/10.64898/2026.09.16.750450; this version posted September 17, 2026. The copyright holder for this preprint (which was not certified by peer review) is the author/funder, who has granted bioRxiv a license to display the preprint in perpetuity. It is made available under a CC-BY 4.0 International license.

G: Dependence of the mean first-passage time on the reset rate r in 1D with diffusion and drift

In §III E ("Random Walk With Resets and Drift") of the main text, we discussed how the prudence of resetting in the presence of a constant cue can be organized by the Péclet number. Here we derive this condition.

We recall that the mean first-passage time to reach $x_T > 0$ starting from the origin in the presence of stochastic ("Poisson") resetting at rate r can be written as,

$$\langle T\rangle(x_T|0) = \frac{1}{r}\left(e^{x_T/x^*} - 1\right), \tag{S102}$$

where the effective length scale is

$$x^* = \frac{2D/v}{\sqrt{1+\frac{4Dr}{v^2}} - 1}. \tag{S103}$$

We consider here $v > 0$, corresponding to drift toward the target, and determine when the mean first-passage time admits an optimum at a finite reset rate. It is convenient to introduce the dimensionless quantity

$$s(r) = \sqrt{1+\frac{4Dr}{v^2}} \tag{S104}$$

together with the additional dimensionless partner,

$$\chi = \frac{vx_T}{2D}. \tag{S105}$$

(Note that this variable already looks suspiciously like a Péclet number, as we will soon see.)

Expressing eqn. (S103) in terms of these definitions, one finds

$$\frac{x_T}{x^*} = \frac{vx_T}{2D}(s-1) = \chi(s-1). \tag{S106}$$

Since $s^2 - 1 = \frac{4Dr}{v^2}$, the prefactor $1/r$ in the mean first passage time can be rewritten in terms of s, resulting in the expression

$$\langle T\rangle(x_T|0) = \frac{4D}{v^2}\,\frac{e^{\chi(s-1)} - 1}{s^2 - 1}. \tag{S107}$$

Our goal is to ask whether the mean first passage time $\langle T\rangle$ has any minimum with respect to a finite value of the resetting rate r. A sufficient condition for this to be true is $\frac{d\langle T\rangle}{dr} = 0$ for some $r > 0$. This condition can be translated in terms of the natural variable s, since the map $r \mapsto s(r)$ is strictly increasing for $r \geq 0$, so extrema of $\langle T\rangle$ as a function of r correspond one-to-one with extrema as a function of s. Thus, our question translates to whether there exists a solution $s > 1$ to this equation,

$$\frac{d}{ds}\left(\frac{e^{\chi(s-1)} - 1}{s^2 - 1}\right) = 0. \tag{S108}$$

This equation itself reduces to the implicit stationarity condition,

$$\frac{\chi e^{\chi(s-1)}}{e^{\chi(s-1)} - 1} = \frac{2s}{s^2 - 1}, \qquad s > 1. \tag{S109}$$

To help analyze when eqn. (S109) admits a solution, we rewrite this condition in terms of yet another parameter, given by $t = s - 1 > 0$ (not to be confused ~~at all~~ with time). This conveniently rewrites the condition as,

$$\frac{\chi}{1 - e^{-\chi t}} = \frac{2(1+t)}{t(2+t)}. \tag{S110}$$

Multiplying both sides by t yields

$$b(\chi t) = a(t), \qquad t > 0, \tag{S111}$$

bioRxiv preprint doi: https://doi.org/10.64898/2026.09.16.750450; this version posted September 17, 2026. The copyright holder for this preprint (which was not certified by peer review) is the author/funder, who has granted bioRxiv a license to display the preprint in perpetuity. It is made available under a CC-BY 4.0 International license.

where we have defined

$$b(x) = \frac{x}{1-e^{-x}}, \qquad a(t) = \frac{2(1+t)}{2+t}. \tag{S112}$$

It is convenient to recast eqn. (S111) as a root-finding problem. We therefore define

$$F(t) = b(\chi t) - a(t), \qquad t \geq 0, \tag{S113}$$

so that stationary points correspond to solutions of $F(t) = 0$ with $t > 0$. The function $a(t)$ is strictly increasing and bounded on $t \geq 0$,

$$a(0) = 1, \qquad \lim_{t\to\infty} a(t) = 2, \qquad a'(t) = \frac{2}{(2+t)^2} > 0. \tag{S114}$$

The function $b(x)$ is strictly increasing and unbounded on $x \geq 0$,

$$\lim_{x\to 0} b(x) = 1, \qquad \lim_{x\to\infty} b(x) = \infty, \tag{S115}$$

and $b'(x) > 0$ for all $x > 0$ since

$$b'(x) = \frac{1-e^{-x}(1+x)}{(1-e^{-x})^2} > 0, \tag{S116}$$

using $(1+x)e^{-x} \leq 1$ for $x \geq 0$. In addition, differentiating once more shows that b is convex on $x \geq 0$,

$$b''(x) = \frac{e^{-x}\left[x-2+(x+2)e^{-x}\right]}{(1-e^{-x})^3} \geq 0, \qquad x \geq 0, \tag{S117}$$

with strict inequality for $x > 0$. Similarly, a is concave on $t \geq 0$ since

$$a''(t) = -\frac{4}{(2+t)^3} < 0. \tag{S118}$$

From eqn. (S113) we have

$$F''(t) = \chi^2 b''(\chi t) - a''(t). \tag{S119}$$

Using $b''(x) \geq 0$ for $x \geq 0$ and $-a''(t) = \frac{4}{(2+t)^3} > 0$, we obtain

$$F''(t) \geq \frac{4}{(2+t)^3} > 0, \qquad t \geq 0. \tag{S120}$$

Thus F is strictly convex on $[0,\infty)$ and $F'(t)$ is strictly increasing. At $t = 0$ we have $F(0) = b(0) - a(0) = 1 - 1 = 0$. The behavior near $t = 0^+$ (which is to say, approaching $t = 0$ starting from positive values to the right of zero) is controlled by the first-order expansion

$$b(\chi t) = 1 + \frac{\chi}{2} t + O(t^2), \qquad a(t) = 1 + \frac{1}{2} t + O(t^2), \tag{S121}$$

which implies

$$F(t) = \frac{\chi - 1}{2} t + O(t^2), \qquad t \to 0^+. \tag{S122}$$

Hence $F(t) > 0$ for all sufficiently small $t > 0$ if $\chi > 1$, whereas $F(t) < 0$ for all sufficiently small $t > 0$ if $\chi < 1$. For large t, eqn. (S115) gives $b(\chi t) \sim \chi t$, while eqn. (S114) gives $a(t) \to 2$. Therefore

$$\lim_{t\to\infty} F(t) = +\infty. \tag{S123}$$

Since $F''(t) > 0$, the derivative $F'(t)$ is strictly increasing on $[0,\infty)$. From the small-t expansion in eqn. (S122), we have

$$F'(0) = \frac{\chi - 1}{2}. \tag{S124}$$

bioRxiv preprint doi: https://doi.org/10.64898/2026.09.16.750450; this version posted September 17, 2026. The copyright holder for this preprint (which was not certified by peer review) is the author/funder, who has granted bioRxiv a license to display the preprint in perpetuity. It is made available under a CC-BY 4.0 International license.

When $\chi \geq 1$, we have $F'(0) \geq 0$. Since F' is strictly increasing, $F'(t) > 0$ for all $t > 0$. Together with $F(0) = 0$, this implies

$$F(t) > 0, \qquad t > 0, \tag{S125}$$

and therefore no extremum exists at finite reset rate.

When $\chi < 1$, we instead have $F'(0) < 0$, so $F(t) < 0$ for sufficiently small positive t. On the other hand,

$$\lim_{t\to\infty} F(t) = +\infty. \tag{S126}$$

By continuity, F must therefore cross zero at some $t^\star > 0$. Moreover, because F' is strictly increasing, F has at most one minimum and can cross zero only once after leaving the origin. Thus there exists a unique solution $t^\star > 0$ when $\chi < 1$. In summary, a finite optimal reset rate exists if and only if

$$\chi = \frac{v x_T}{2D} < 1, \tag{S127}$$

in which case the minimizer is unique. Since the Péclet number over the target distance is

$$\mathrm{Pe} = \frac{v x_T}{D}, \tag{S128}$$

this condition is equivalently

$$\mathrm{Pe} < 2. \tag{S129}$$

Thus, a finite optimal reset rate exists only when diffusion is sufficiently strong relative to drift.

Another route to appreciate that the Péclet number's value relative to 2 sets the prudence of reset can be seen more directly via the immediate expansion of eqn. 107 of the main text. The foregoing analysis is a more complete and rigorous articulation of this core behavior.

H: Random walk with reset and exponential cue

1. Nondimensionalization of the exponential-cue backward equation

In §III F of the main text ("Random Seeker with an Exponential Cue"), we tamed a little of the complexity of learning how different reset policies vary with the properties of directional cues (whose strength also vary with position) by expressing the differential equation for the first passage time in terms of a smaller number of non-dimensional parameters.

Here we explicitly nondimensionalize this eqn. 113 of the main text. We first measure position in units of the location of the target and time in units of the time to diffuse to the target, yielding the dimensionless positions and first passage times,

$$\tilde{x} = \frac{x}{x_T}, \qquad \tilde{T}(\tilde{x}) = \frac{D}{x_T^2} T(x), \tag{S130}$$

or equivalently,

$$x = x_T \tilde{x}, \qquad T(x) = \frac{x_T^2}{D} \tilde{T}(\tilde{x}). \tag{S131}$$

We first remind ourselves how derivatives with respect to x are related to derivatives with respect to $\tilde{x}$. We have

$$\frac{dx}{d\tilde{x}} = x_T. \tag{S132}$$

Starting from

$$\tilde{T}(\tilde{x}) = \frac{D}{x_T^2} T(x_T \tilde{x}), \tag{S133}$$

we differentiate with respect to $\tilde{x}$. By the chain rule,

$$\frac{d\tilde{T}}{d\tilde{x}} = \frac{D}{x_T^2} \frac{dT}{dx} \frac{dx}{d\tilde{x}} \tag{S134}$$

$$= \frac{D}{x_T^2} T'(x) x_T \tag{S135}$$

$$= \frac{D}{x_T} T'(x). \tag{S136}$$

bioRxiv preprint doi: https://doi.org/10.64898/2026.09.16.750450; this version posted September 17, 2026. The copyright holder for this preprint (which was not certified by peer review) is the author/funder, who has granted bioRxiv a license to display the preprint in perpetuity. It is made available under a CC-BY 4.0 International license.

Therefore,

$$T'(x) = \frac{x_T}{D}\tilde{T}'(\tilde{x}). \tag{S137}$$

We can apply the same procedure once more to obtain the second derivative. Differentiating the previous relation with respect to $\tilde{x}$ gives

$$\frac{d^2\tilde{T}}{d\tilde{x}^2} = \frac{D}{x_T}\frac{d}{d\tilde{x}}T'(x) \tag{S138}$$

$$= \frac{D}{x_T}\frac{dT'}{dx}\frac{dx}{d\tilde{x}} \tag{S139}$$

$$= \frac{D}{x_T}T''(x)x_T \tag{S140}$$

$$= DT''(x). \tag{S141}$$

Hence,

$$T''(x) = \frac{1}{D}\tilde{T}''(\tilde{x}). \tag{S142}$$

Substituting these relations, together with $\tilde{\ell} = \ell_c/x_T$, into eqn. 113 gives

$$0 = 1 + \tilde{T}''(\tilde{x}) + \frac{v_* x_T}{D}e^{-(1-\tilde{x})/\tilde{\ell}}\tilde{T}'(\tilde{x}) - \frac{rx_T^2}{D}\tilde{T}(\tilde{x}) + \frac{rx_T^2}{D}\tilde{T}(0). \tag{S143}$$

Using the dimensionless parameters,

$$\mathrm{Pe} = \frac{v_* x_T}{D}, \qquad \tilde{T}_R = \frac{D}{rx_T^2}, \tag{S144}$$

we obtain the dimensionless backward equation

$$0 = 1 + \tilde{T}''(\tilde{x}) + \mathrm{Pe}\, e^{-(1-\tilde{x})/\tilde{\ell}}\tilde{T}'(\tilde{x}) - \frac{\tilde{T}(\tilde{x})}{\tilde{T}_R} + \frac{\tilde{T}(0)}{\tilde{T}_R}. \tag{S145}$$

This is the desired dimensionless eqn. 116 of the main text.

2. Solving the backward equation for the mean first-passage time

We now solve the backward equation associated with the exponentially localized cue. To make further progress understanding this equation, we now introduce an auxiliary function $\phi(\tilde{x})$ that captures the position-dependent part of the mean first-passage time, thus expressing the dynamics as

$$\tilde{T}(\tilde{x}) = \left[\tilde{T}(0) + \tilde{T}_R\right]\left[1 - \phi(\tilde{x})\right]. \tag{S146}$$

Substituting this change of variables into eqn. 116, the constant terms cancel and ϕ satisfies the homogeneous equation

$$\phi''(\tilde{x}) + \mathrm{Pe}\, e^{-(1-\tilde{x})/\tilde{\ell}}\phi'(\tilde{x}) - \frac{1}{\tilde{T}_R}\phi(\tilde{x}) = 0. \tag{S147}$$

The absorbing boundary condition $\tilde{T}(1) = 0$ gives

$$\phi(1) = 1. \tag{S148}$$

Evaluating eqn. S146 at the starting point $\tilde{x} = 0$ gives

$$\tilde{T}(0) = \tilde{T}_R\left(\frac{1}{\phi(0)} - 1\right). \tag{S149}$$

bioRxiv preprint doi: https://doi.org/10.64898/2026.09.16.750450; this version posted September 17, 2026. The copyright holder for this preprint (which was not certified by peer review) is the author/funder, who has granted bioRxiv a license to display the preprint in perpetuity. It is made available under a CC-BY 4.0 International license.

As $\tilde{x} \to -\infty$, the cue vanishes exponentially and eqn. S147 reduces to

$$\phi'' - \frac{1}{\tilde{T}_R}\phi = 0. \tag{S150}$$

Requiring the solution to remain bounded as $\tilde{x} \to -\infty$ selects the decaying exponential branch,

$$\phi(\tilde{x}) \propto e^{\tilde{x}/\sqrt{\tilde{T}_R}} \qquad (\tilde{x} \to -\infty). \tag{S151}$$

When the cue is absent, this far-field equation holds everywhere, and the normalization $\phi(1) = 1$ of eqn. S148 gives

$$\phi(\tilde{x}) = e^{(\tilde{x}-1)/\sqrt{\tilde{T}_R}}. \tag{S152}$$

Substituting $\phi(0) = e^{-1/\sqrt{\tilde{T}_R}}$ into eqn. S149 recovers the result obtained previously for diffusion with resetting of eqn. 57. For the exponential cue, eqn. S147 admits a closed-form solution for $\phi(\tilde{x})$ in terms of hypergeometric functions that we now derive.

We show here that this equation can be transformed into Kummer's equation and therefore solved in terms of confluent hypergeometric functions[1].

We introduce the change of variables

$$z = \tilde{\ell}\,\mathrm{Pe}\, e^{-(1-\tilde{x})/\tilde{\ell}}. \tag{S153}$$

For $\tilde{\ell} > 0$ and $\mathrm{Pe} > 0$, this function is smooth and monotone. Since

$$\frac{dz}{d\tilde{x}} = \frac{z}{\tilde{\ell}}, \tag{S154}$$

we have

$$\frac{d\phi}{d\tilde{x}} = \frac{z}{\tilde{\ell}}\frac{d\phi}{dz}, \tag{S155}$$

and

$$\frac{d^2\phi}{d\tilde{x}^2} = \frac{z}{\tilde{\ell}^2}\frac{d\phi}{dz} + \left(\frac{z}{\tilde{\ell}}\right)^2 \frac{d^2\phi}{dz^2}. \tag{S156}$$

Substituting these expressions into eqn. S147 gives

$$z^2\phi''(z) + z(z+1)\phi'(z) - \nu^2\phi(z) = 0, \tag{S157}$$

where

$$\nu = \frac{\tilde{\ell}}{\sqrt{\tilde{T}_R}}. \tag{S158}$$

To reduce this equation to the Kummer equation, we write

$$\phi(z) = z^\nu u(z). \tag{S159}$$

Then

$$\phi'(z) = \nu z^{\nu-1}u(z) + z^\nu u'(z), \tag{S160}$$

and

$$\phi''(z) = \nu(\nu-1)z^{\nu-2}u(z) + 2\nu z^{\nu-1}u'(z) + z^\nu u''(z). \tag{S161}$$

Substitution into eqn. S157 yields

$$zu''(z) + (2\nu+1+z)u'(z) + \nu u(z) = 0. \tag{S162}$$

Finally, defining $s = -z$, we obtain

$$su''(s) + (2\nu+1-s)u'(s) - \nu u(s) = 0. \tag{S163}$$

bioRxiv preprint doi: https://doi.org/10.64898/2026.09.16.750450; this version posted September 17, 2026. The copyright holder for this preprint (which was not certified by peer review) is the author/funder, who has granted bioRxiv a license to display the preprint in perpetuity. It is made available under a CC-BY 4.0 International license.

This is exactly Kummer's equation[1], defined as,

$$su''(s) + (b-s)u'(s) - au(s) = 0, \tag{S164}$$

with parameters

$$a = \nu, \qquad b = 2\nu + 1. \tag{S165}$$

A standard solution is the confluent hypergeometric function of the first kind[1],

$$M(a,b,s) = \sum_{n=0}^{\infty} \frac{(a)_n}{(b)_n} \frac{s^n}{n!}, \tag{S166}$$

where $(a)_0 = 1$ and

$$(a)_n = a(a+1)\cdots(a+n-1) \tag{S167}$$

is the rising factorial. A second linearly independent solution is given by Tricomi's confluent hypergeometric function $U(a,b,s)$[1]. Thus the general solution can be written as

$$u(s) = C_1 M(a,b,s) + C_2 U(a,b,s). \tag{S168}$$

Returning to the original variable $\tilde{x}$, we find

$$\begin{aligned}\phi(\tilde{x}) = D_1 e^{-(1-\tilde{x})/\sqrt{\tilde{T}_R}} M\left(\nu, 2\nu+1, -\tilde{\ell}, \mathrm{Pe}, e^{-(1-\tilde{x})/\tilde{\ell}}\right) \\ + D_2 e^{-(1-\tilde{x})/\sqrt{\tilde{T}_R}} U\left(\nu, 2\nu+1, -\tilde{\ell}, \mathrm{Pe}, e^{-(1-\tilde{x})/\tilde{\ell}}\right),\end{aligned} \tag{S169}$$

where D_1 and D_2 are constants.

The physically relevant solution is selected by considering the far-left limit $\tilde{x} \to -\infty$, where the cue vanishes. In this limit, the solution should reduce to the decaying no-cue form

$$\phi(\tilde{x}) \propto e^{-(1-\tilde{x})/\sqrt{\tilde{T}_R}}. \tag{S170}$$

Since $M(\nu, 2\nu+1, 0) = 1$, the M branch has precisely this behavior. By contrast, the U branch is singular as $\tilde{x} \to -\infty$ for $\nu > 0$ and gives the growing solution. We therefore set $D_2 = 0$.

The remaining constant is fixed by the normalization condition $\phi(1) = 1$. This gives

$$\phi(\tilde{x}) = e^{-(1-\tilde{x})/\sqrt{\tilde{T}_R}} \frac{M\left(\nu, 2\nu+1, -\tilde{\ell}, \mathrm{Pe}, e^{-(1-\tilde{x})/\tilde{\ell}}\right)}{M\left(\nu, 2\nu+1, -\tilde{\ell}, \mathrm{Pe}\right)}. \tag{S171}$$

In particular, evaluating this expression for an initial position at $\tilde{x} = 0$ gives

$$\phi(0) = e^{-1/\sqrt{\tilde{T}_R}} \frac{M\left(\nu, 2\nu+1, -\tilde{\ell}, \mathrm{Pe}, e^{-1/\tilde{\ell}}\right)}{M\left(\nu, 2\nu+1, -\tilde{\ell}, \mathrm{Pe}\right)}. \tag{S172}$$

The dimensionless mean first-passage time from the reset position is therefore finally,

$$\tilde{T}(0) = \tilde{T}_R \left(\frac{1}{\phi(0)} - 1\right). \tag{S173}$$

This is eqn. S149.

Numerical analyses in the main text in the (Pe, ℓ) plane are found as follows. To appreciate the behavior of this larger physical problem, we evaluate the solution of eqn. S150 numerically at $\tilde{x} = 0$ via $\tilde{\phi}(0)$ and then compute the mean first-passage time using eqn. S149.

bioRxiv preprint doi: https://doi.org/10.64898/2026.09.16.750450; this version posted September 17, 2026. The copyright holder for this preprint (which was not certified by peer review) is the author/funder, who has granted bioRxiv a license to display the preprint in perpetuity. It is made available under a CC-BY 4.0 International license.

3. Asymptotic checks for the exponential cue solution

a. Large Pe for fixed $\tilde{\ell}$ and $\tilde{T}_R$

We now record a few useful asymptotic limits of the exact solution. We first look at the large Peclet limit. We first consider the limit of Pe going to infinity at fixed $\tilde{\ell}$ and fixed $\tilde{T}_R$. Using the large negative argument asymptotic behavior of Kummer's function[1],

$$M(a,b,-y) \sim \frac{\Gamma(b)}{\Gamma(b-a)} y^{-a}, \qquad y \to +\infty, \tag{S174}$$

Using this asymptotic form, we find

$$\phi(0) \to 1, \qquad \mathrm{Pe} \to \infty. \tag{S175}$$

Since the dimensionless mean first-passage time is

$$\tilde{T}(0) = \tilde{T}_R \left(\frac{1}{\phi(0)} - 1 \right), \tag{S176}$$

this immediately implies

$$\tilde{T}(0) \to 0, \qquad \mathrm{Pe} \to \infty. \tag{S177}$$

As the Péclet number becomes large, the drift toward the target dominates diffusion and resetting. In the limit $\mathrm{Pe} \to \infty$, the walker is transported to the target infinitely rapidly, so the mean first-passage time vanishes. To understand better how the mean first passage time converges to 0 in the limit of large Pe number, we keep the first order correction (given by Mathematica),

$$\phi(0) = 1 - \frac{\tilde{\ell}\left(e^{1/\tilde{\ell}} - 1\right)}{\tilde{T}_R \mathrm{Pe}} + O\left(\mathrm{Pe}^{-2}\right). \tag{S178}$$

Therefore

$$\tilde{T}(0) = \frac{\tilde{\ell}\left(e^{1/\tilde{\ell}} - 1\right)}{\mathrm{Pe}} + O\left(\mathrm{Pe}^{-2}\right). \tag{S179}$$

This leading term is exactly the deterministic drift time from $\tilde{x} = 0$ to $\tilde{x} = 1$ in the velocity field

$$\tilde{v}(\tilde{x}) = \mathrm{Pe}\, e^{-(1-\tilde{x})/\tilde{\ell}}. \tag{S180}$$

Indeed,

$$\tilde{t}_{\mathrm{drift}} = \int_0^1 \frac{d\tilde{x}}{\mathrm{Pe}\, e^{-(1-\tilde{x})/\tilde{\ell}}} = \frac{1}{\mathrm{Pe}} \int_0^1 e^{(1-\tilde{x})/\tilde{\ell}} d\tilde{x} = \frac{\tilde{\ell}\left(e^{1/\tilde{\ell}} - 1\right)}{\mathrm{Pe}}. \tag{S181}$$

Thus, in the strong-cue limit, the mean first-passage time approaches the drift-limited travel time. Figure S23 shows that the numerical solution converges to this asymptotic prediction at large Pe.

bioRxiv preprint doi: https://doi.org/10.64898/2026.09.16.750450; this version posted September 17, 2026. The copyright holder for this preprint (which was not certified by peer review) is the author/funder, who has granted bioRxiv a license to display the preprint in perpetuity. It is made available under a CC-BY 4.0 International license.

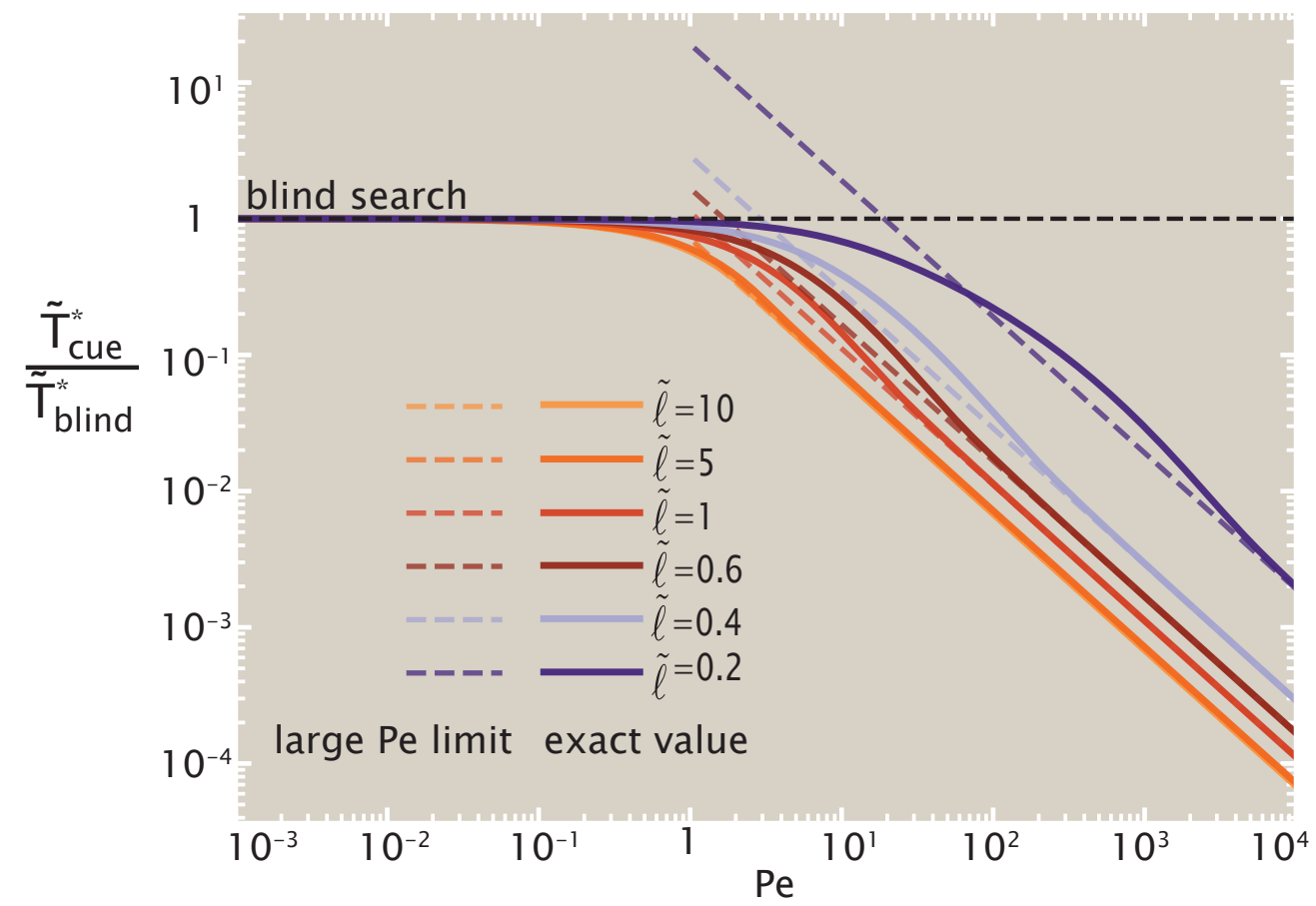

FIG. S23: Large-Pe behavior of the optimized search time. Solid curves show the numerical result for different cue lengths $\tilde{\ell}$, while dashed curves show the large-Pe drift-time asymptote. The numerical solution approaches the predicted asymptotic behavior as Pe increases.

The deterministic drift time also suggests a natural dimensionless quantity for characterizing when the cue substantially accelerates the search. We compare it to the optimal blind-search time and define

$$\Theta_{\text{drift}} = \frac{\tilde{t}_{\text{drift}}}{\tilde{T}^*_{\text{blind}}} = \frac{\tilde{\ell}\left(e^{1/\tilde{\ell}} - 1\right)}{\text{Pe}\,\tilde{T}^*_{\text{blind}}}. \tag{S182}$$

When $\Theta_{\text{drift}} \gg 1$, following the cue deterministically would be slower than the characteristic blind-search time, whereas when $\Theta_{\text{drift}} \ll 1$, the cue provides a much faster route to the target.

Figure S24 replots the optimized search-time ratio (previously shown in the (ℓ, Pe) plane in Fig. 22 of the main text in the $(\Theta_{\text{drift}}, \tilde{\ell})$ plane instead. The nearly vertical contours show that much of the dependence of the optimized search time on Pe and $\tilde{\ell}$ is captured by the single combination Θ_{drift}.

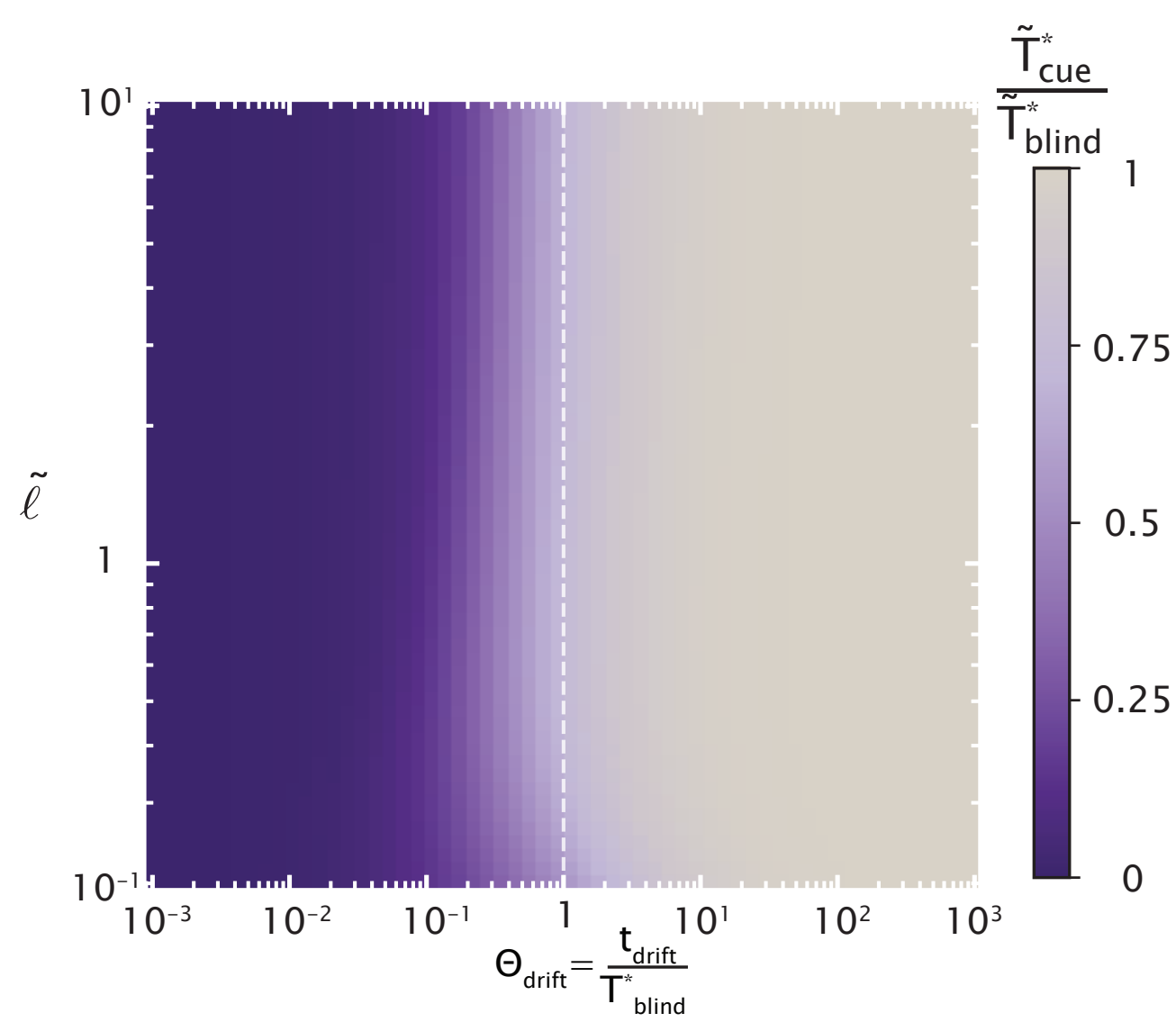

FIG. S24: Optimized search time expressed in terms of the normalized deterministic drift time $\Theta_{\text{drift}} = \tilde{t}_{\text{drift}}/\tilde{T}^*_{\text{blind}}$. The color indicates the optimized search time in the presence of the cue, normalized by the optimal blind-search time, $\tilde{T}^*_{\text{cue}}/\tilde{T}^*_{\text{blind}}$.

bioRxiv preprint doi: https://doi.org/10.64898/2026.09.16.750450; this version posted September 17, 2026. The copyright holder for this preprint (which was not certified by peer review) is the author/funder, who has granted bioRxiv a license to display the preprint in perpetuity. It is made available under a CC-BY 4.0 International license.

b. Large $\tilde{T}_R$ for fixed $\tilde{\ell}$ and Pe

We next consider the limit of large reset time, $\tilde{T}_R \to \infty$, corresponding to the no-reset regime. For a purely diffusive walker in one dimension, removing resetting leads to an infinite mean first-passage time. Here, however, the walker experiences a drift toward the target. We therefore ask whether the exponentially localized cue is sufficient to produce a finite mean first-passage time at fixed, finite Pe and $\tilde{\ell}$.

To study this limit, we introduce the small parameter

$$\varepsilon = \frac{1}{\sqrt{\tilde{T}_R}}, \tag{S183}$$

so that $\varepsilon \to 0$ as $\tilde{T}_R \to \infty$. The equation for the auxiliary function ϕ can then be written exactly as

$$\phi''(\tilde{x}) + \mathrm{Pe}\, e^{-(1-\tilde{x})/\tilde{\ell}} \phi'(\tilde{x}) - \varepsilon^2 \phi(\tilde{x}) = 0. \tag{S184}$$

The large-$\tilde{T}_R$ limit is slightly subtle. Although ε appears explicitly in eqn. S184 only through ε^2, the far-field boundary condition contains a contribution already at order ε. Indeed, as $\tilde{x} \to -\infty$, the cue vanishes and eqn. S184 reduces to

$$\phi'' - \varepsilon^2 \phi = 0. \tag{S185}$$

Requiring the solution to remain bounded as $\tilde{x} \to -\infty$ gives

$$\phi(\tilde{x}) \propto e^{\varepsilon \tilde{x}}, \tag{S186}$$

and therefore

$$\frac{\phi'(\tilde{x})}{\phi(\tilde{x})} \longrightarrow \varepsilon, \qquad \tilde{x} \to -\infty. \tag{S187}$$

At leading order, $\varepsilon = 0$, and eqn. S184 reduces to

$$\phi_0''(\tilde{x}) + \mathrm{Pe}\, e^{-(1-\tilde{x})/\tilde{\ell}} \phi_0'(\tilde{x}) = 0. \tag{S188}$$

At the same order, the far-field condition $\phi'/\phi \to \varepsilon$ becomes

$$\phi_0'(-\infty) = 0. \tag{S189}$$

Defining $q_0(\tilde{x}) = \phi_0'(\tilde{x})$, we obtain

$$q_0' + \mathrm{Pe}\, e^{-(1-\tilde{x})/\tilde{\ell}} q_0 = 0. \tag{S190}$$

Since $q_0(-\infty) = 0$, the solution is $q_0(\tilde{x}) = 0$ everywhere. Therefore ϕ_0 is constant. Finally, the normalization $\phi_0(1) = 1$ fixes this constant, giving

$$\phi_0(\tilde{x}) = 1. \tag{S191}$$

To determine the order of the first correction, we expand

$$\phi(\tilde{x}) = \phi_0(\tilde{x}) + \varepsilon \psi(\tilde{x}) + O(\varepsilon^2). \tag{S192}$$

At leading order, $\phi_0(\tilde{x}) = 1$. We then match this expansion to the far-field condition eqn. S187 . Substituting the expansion gives

$$\frac{\phi'}{\phi} = \varepsilon \psi'(\tilde{x}) + O(\varepsilon^2), \tag{S193}$$

and therefore matching at order ε requires

$$\psi'(-\infty) = 1. \tag{S194}$$

Thus the coefficient of the order-ε correction is nonzero, and the expansion must contain a term linear in ε:

$$\phi(\tilde{x}) = 1 + \varepsilon \psi(\tilde{x}) + O(\varepsilon^2). \tag{S195}$$

bioRxiv preprint doi: https://doi.org/10.64898/2026.09.16.750450; this version posted September 17, 2026. The copyright holder for this preprint (which was not certified by peer review) is the author/funder, who has granted bioRxiv a license to display the preprint in perpetuity. It is made available under a CC-BY 4.0 International license.

Substituting this expression into eqn. S184, we have

$$\varepsilon\psi''(\tilde{x}) + \mathrm{Pe}\, e^{-(1-\tilde{x})/\tilde{\ell}}\varepsilon\psi'(\tilde{x}) - \varepsilon^2\left[1+\varepsilon\psi(\tilde{x})\right] + O(\varepsilon^2) = 0. \tag{S196}$$

Dividing by ε and keeping only the leading terms as $\varepsilon \to 0$, we obtain

$$\psi''(\tilde{x}) + \mathrm{Pe}\, e^{-(1-\tilde{x})/\tilde{\ell}}\psi'(\tilde{x}) = 0. \tag{S197}$$

Introducing

$$q(\tilde{x}) = \psi'(\tilde{x}), \tag{S198}$$

we obtain the first-order equation

$$q'(\tilde{x}) + \mathrm{Pe}\, e^{-(1-\tilde{x})/\tilde{\ell}} q(\tilde{x}) = 0. \tag{S199}$$

Integrating gives

$$q(\tilde{x}) = C\exp\left[-\tilde{\ell}\,\mathrm{Pe}\, e^{-(1-\tilde{x})/\tilde{\ell}}\right]. \tag{S200}$$

Since $q(-\infty) = 1$, the integration constant is $C = 1$, and hence

$$\psi'(\tilde{x}) = \exp\left[-\tilde{\ell}\,\mathrm{Pe}\, e^{-(1-\tilde{x})/\tilde{\ell}}\right]. \tag{S201}$$

Using $\psi(1) = 0$, we then find

$$\psi(0) = -\int_0^1 \exp\left[-\tilde{\ell}\,\mathrm{Pe}\, e^{-(1-\tilde{x})/\tilde{\ell}}\right] d\tilde{x}. \tag{S202}$$

It is therefore convenient to define

$$A(\mathrm{Pe},\tilde{\ell}) = \int_0^1 \exp\left[-\tilde{\ell}\,\mathrm{Pe}\, e^{-(1-\tilde{x})/\tilde{\ell}}\right] d\tilde{x}, \tag{S203}$$

so that

$$\phi(0) = 1 - \frac{A(\mathrm{Pe},\tilde{\ell})}{\sqrt{\tilde{T}_R}} + O\left(\tilde{T}_R^{-1}\right). \tag{S204}$$

Using

$$\tilde{T}(0) = \tilde{T}_R\left(\frac{1}{\phi(0)} - 1\right), \tag{S205}$$

we obtain

$$\frac{1}{\phi(0)} - 1 = \frac{A(\mathrm{Pe},\tilde{\ell})}{\sqrt{\tilde{T}_R}} + O\left(\tilde{T}_R^{-1}\right), \tag{S206}$$

and therefore

$$\tilde{T}(0) \sim A(\mathrm{Pe},\tilde{\ell})\sqrt{\tilde{T}_R} \qquad \tilde{T}_R \to \infty. \tag{S207}$$

Thus, at any fixed finite Pe and $\tilde{\ell}$, the exponentially localized cue does not regularize the no-reset problem: the mean first-passage time diverges as $\sqrt{\tilde{T}_R}$ as the reset time becomes large. The coefficient $A(\mathrm{Pe},\tilde{\ell})$ is positive for $\tilde{\ell} > 0$ and $\mathrm{Pe} > 0$. Therefore,

$$\tilde{T}(0) \to \infty \qquad \text{as} \qquad \tilde{T}_R \to \infty. \tag{S208}$$

Thus, for finite Pe and finite $\tilde{\ell}$, taking the no-reset limit makes the mean first-passage time diverge. This confirms that the optimum reset time remains finite at finite cue strength.

bioRxiv preprint doi: https://doi.org/10.64898/2026.09.16.750450; this version posted September 17, 2026. The copyright holder for this preprint (which was not certified by peer review) is the author/funder, who has granted bioRxiv a license to display the preprint in perpetuity. It is made available under a CC-BY 4.0 International license.

We now consider the joint limit of large Péclet number and large nondimensional reset time. The motivation for this limit is that, at high Péclet number, the mean first-passage time approaches the deterministic drift-dominated travel time. In the formal limit $\mathrm{Pe} \to \infty$, this behavior holds for any reset time, since the drift is strong enough to bring the searcher to the target almost immediately. For large but finite Pe, however, we expect the relevant reset time to be large but finite. Indeed, resets must be sufficiently rare to allow the drift to carry the searcher to the target, but the reset time cannot be taken to infinity at fixed Pe, since we have shown that the mean first-passage time diverges in the no-reset limit. This motivates studying the combined asymptotic regime in which both Pe and $\tilde{T}_R$ are large. Introducing the cue-scale Péclet number

$$\mathrm{Pe}_{\mathrm{cue},0} = \tilde{\ell}\,\mathrm{Pe}\, e^{-1/\tilde{\ell}}, \tag{S209}$$

the prefactor can be written as

$$A(\mathrm{Pe},\tilde{\ell}) = \int_0^1 \exp\left[-\mathrm{Pe}_{\mathrm{cue},0} e^{\tilde{x}/\tilde{\ell}}\right] d\tilde{x}. \tag{S210}$$

For $\mathrm{Pe}_{\mathrm{cue},0} \gg 1$, this integral is dominated by $\tilde{x} \simeq 0$. Expanding

$$e^{\tilde{x}/\tilde{\ell}} \simeq 1 + \frac{\tilde{x}}{\tilde{\ell}}, \tag{S211}$$

we obtain

$$A(\mathrm{Pe},\tilde{\ell}) \sim e^{-\mathrm{Pe}_{\mathrm{cue},0}} \int_0^\infty \exp\left[-\frac{\mathrm{Pe}_{\mathrm{cue},0}}{\tilde{\ell}}\tilde{x}\right] d\tilde{x} \tag{S212}$$

$$= \frac{\tilde{\ell}}{\mathrm{Pe}_{\mathrm{cue},0}} e^{-\mathrm{Pe}_{\mathrm{cue},0}}. \tag{S213}$$

Equivalently,

$$A(\mathrm{Pe},\tilde{\ell}) \sim \frac{e^{1/\tilde{\ell}}}{\mathrm{Pe}} \exp\left[-\tilde{\ell}\mathrm{Pe} e^{-1/\tilde{\ell}}\right]. \tag{S214}$$

Therefore, in the large-$\tilde{T}_R$ and large-Pe regime,

$$\tilde{T}(0) \sim \frac{e^{1/\tilde{\ell}}}{\mathrm{Pe}} \exp\left[-\tilde{\ell}\mathrm{Pe}\, e^{-1/\tilde{\ell}}\right] \sqrt{\tilde{T}_R}. \tag{S215}$$

This expression shows that, although $\tilde{T}(0)$ still diverges as $\tilde{T}_R \to \infty$ at any fixed Pe, the prefactor of this divergence becomes exponentially small when the cue is strong at the reset position.

Finally, we get an estimate for the optimal reset time in the strong-cue regime. A simple estimate for the reset time at which the large-$\tilde{T}_R$ asymptotic behavior reaches this drift-limited scale is obtained by matching

$$\frac{e^{1/\tilde{\ell}}}{\mathrm{Pe}} \exp\left[-\tilde{\ell}\mathrm{Pe}\, e^{-1/\tilde{\ell}}\right] \sqrt{\tilde{T}_R} \sim \tilde{t}_{\mathrm{drift}} = \frac{\tilde{\ell}\left(e^{1/\tilde{\ell}} - 1\right)}{\mathrm{Pe}}. \tag{S216}$$

After canceling the common factor $1/\mathrm{Pe}$, this gives

$$\sqrt{\tilde{T}_R} \sim \tilde{\ell}\left(1 - e^{-1/\tilde{\ell}}\right) \exp\left[\tilde{\ell}\mathrm{Pe}\, e^{-1/\tilde{\ell}}\right]. \tag{S217}$$

Thus the corresponding reset time is

$$\tilde{T}_R \sim \tilde{\ell}^2 \left(1 - e^{-1/\tilde{\ell}}\right)^2 \exp\left[2\tilde{\ell}\mathrm{Pe}\, e^{-1/\tilde{\ell}}\right]. \tag{S218}$$

This scale grows exponentially with Pe at fixed $\tilde{\ell}$. Hence, in the strong-cue regime, the optimal reset time is pushed to very large values. Physically, this corresponds to the fact that the search is already close to drift-limited: resetting becomes less useful because it interrupts an almost deterministic motion toward the target. Thus this estimate gives the large-Pe scaling of the optimal reset time, rather than an exact finite-Pe formula.

bioRxiv preprint doi: https://doi.org/10.64898/2026.09.16.750450; this version posted September 17, 2026. The copyright holder for this preprint (which was not certified by peer review) is the author/funder, who has granted bioRxiv a license to display the preprint in perpetuity. It is made available under a CC-BY 4.0 International license.

4. Existence of a minimum for the optimal reset time

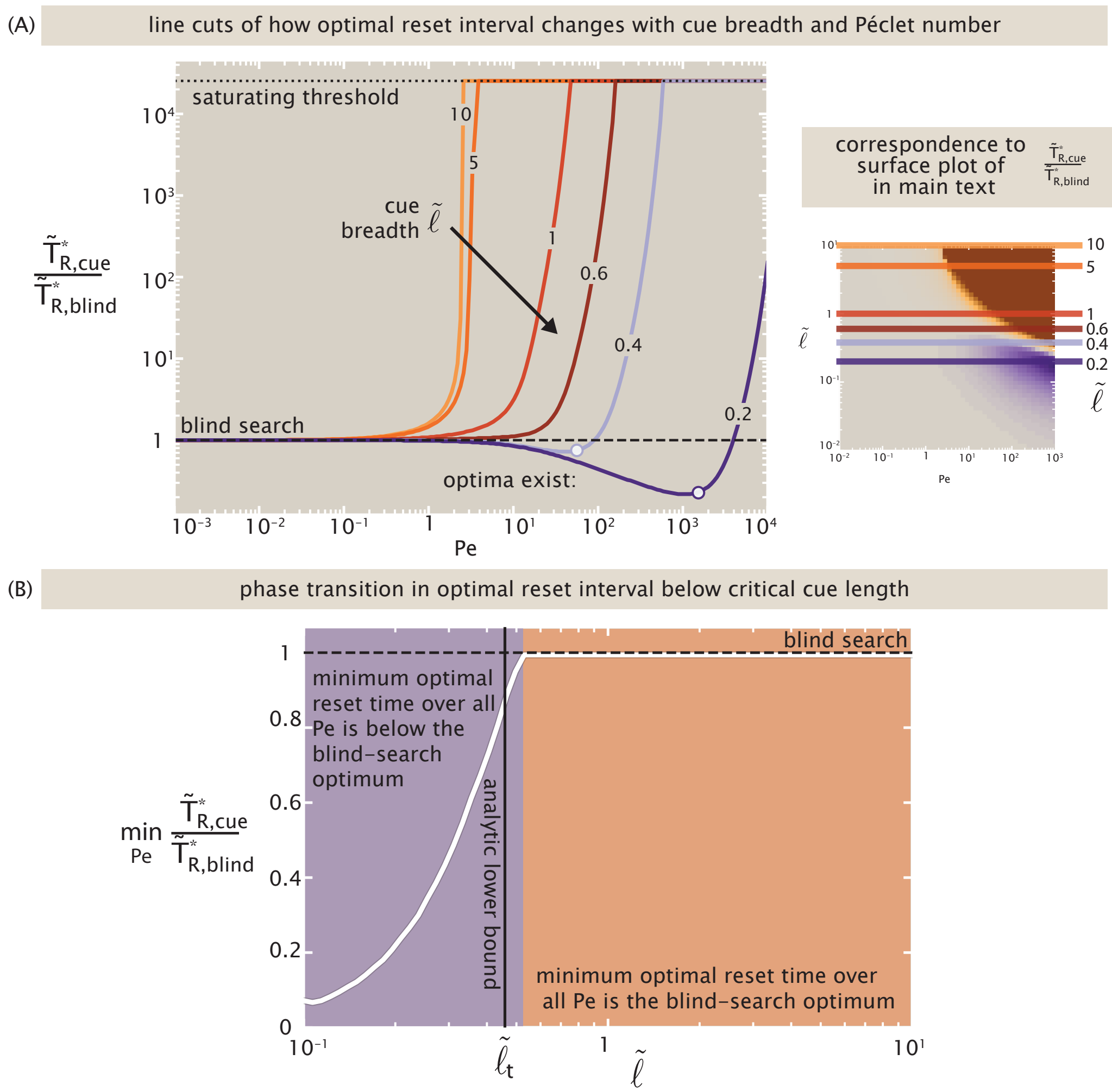

FIG. S25: Localized cues can favor more frequent resetting. (A) Optimal reset time relative to the blind-search optimum as a function of Pe for several cue lengths $\tilde{\ell}$. Values above 10^4 are saturated for visualization, reflecting the divergence of the optimal reset time in the strong-cue regime. (The heatmap of Fig. 22(A) of the main text is repeated at right as a guide to which slices through the landscape the plot at left shows.) (B) Minimum optimal reset-time ratio over cue strength as a function of $\tilde{\ell}$. The vertical line marks the analytic lower bound derived from the weak-cue expansion.

Figure 22(A) of the main text, plotting the optimal reset interval $\tilde{T}^*_{R,\text{cue}}$ (relative to the blind search value), revealed the onset of a surprising regime (visible as a purple island in the heatmap). Here, a moderately localized cue paired with a high-drift was shown to recommend resetting at a *smaller* rate than in blind search. Thus, while broad and strong cues suppress resetting, a localized cue can instead favor more frequent resetting. Here we discuss the onset of this regime in more quantitative detail.

Figure S25(A) inspects line cuts through the heatmap's surface at various values of the cue breadth ℓ. Specifically, note that curves shown with $\ell \lesssim 0.4$ present minima in $\tilde{T}^*_{R,\text{cue}}/\tilde{T}^*_{R,\text{blind}}$ less than one, and are colored in purple. To characterize when this behavior occurs, for each cue length we consider

$$\min_{\text{Pe}} \frac{\tilde{T}^*_{R,\text{cue}}}{\tilde{T}^*_{R,\text{blind}}}, \tag{S219}$$

as shown in Fig. S25(B). Note the explicit onset of a discrete transition in optimal reset policy. A value below one means that there exists some cue strength for which the optimal resetting is more frequent than in blind search. Numerically, this occurs only for sufficiently localized cues.

We can obtain an analytic lower bound on this threshold by examining the response of the optimal reset time to an infinitesi-

bioRxiv preprint doi: https://doi.org/10.64898/2026.09.16.750450; this version posted September 17, 2026. The copyright holder for this preprint (which was not certified by peer review) is the author/funder, who has granted bioRxiv a license to display the preprint in perpetuity. It is made available under a CC-BY 4.0 International license.

mally weak cue. It is convenient to use the cue-scale Péclet number

$$\mathrm{Pe}_{\mathrm{cue},0} = \tilde{\ell}\,\mathrm{Pe}\, e^{-1/\tilde{\ell}}. \tag{S220}$$

At fixed $\tilde{\ell}$, the weak-cue limit corresponds to $\mathrm{Pe}_{\mathrm{cue},0} \to 0$.

We write

$$\alpha = \frac{1}{\sqrt{\tilde{T}_R}}, \qquad \nu = \alpha\tilde{\ell}, \tag{S221}$$

so that $\tilde{T}_R = 1/\alpha^2$. The mean first-passage time is

$$\tilde{T}(0;\alpha) = \frac{1}{\alpha^2}\left(\frac{1}{\phi(0)} - 1\right). \tag{S222}$$

We recall the hypergeometric solution,

$$\phi(0) = \frac{\mathrm{Pe}_{\mathrm{cue},0}^{\nu} M(\nu, 2\nu+1, -\mathrm{Pe}_{\mathrm{cue},0})}{\left(\mathrm{Pe}_{\mathrm{cue},0} e^{1/\tilde{\ell}}\right)^{\nu} M\left(\nu, 2\nu+1, -\mathrm{Pe}_{\mathrm{cue},0} e^{1/\tilde{\ell}}\right)}. \tag{S223}$$

For $\mathrm{Pe}_{\mathrm{cue},0} \ll 1$, we use

$$M(a,b,-z) = 1 - \frac{a}{b} z + O(z^2). \tag{S224}$$

The prefactor in $\phi(0)$ simplifies as

$$\left(\frac{\mathrm{Pe}_{\mathrm{cue},0}}{\mathrm{Pe}_{\mathrm{cue},0} e^{1/\tilde{\ell}}}\right)^{\nu} = e^{-\nu/\tilde{\ell}} = e^{-\alpha}. \tag{S225}$$

Therefore,

$$\phi(0) = e^{-\alpha} \frac{1 - \frac{\nu}{2\nu+1}\mathrm{Pe}_{\mathrm{cue},0} + O(\mathrm{Pe}_{\mathrm{cue},0}^2)}{1 - \frac{\nu}{2\nu+1} e^{1/\tilde{\ell}}\mathrm{Pe}_{\mathrm{cue},0} + O(\mathrm{Pe}_{\mathrm{cue},0}^2)}. \tag{S226}$$

Using

$$\frac{1-az}{1-bz} = 1 + (b-a)z + O(z^2), \tag{S227}$$

we obtain

$$\phi(0) = e^{-\alpha}\left[1 + \frac{\nu}{2\nu+1}\left(e^{1/\tilde{\ell}} - 1\right)\mathrm{Pe}_{\mathrm{cue},0} + O(\mathrm{Pe}_{\mathrm{cue},0}^2)\right]. \tag{S228}$$

It is useful to define

$$B_{\tilde{\ell}}(\alpha) = \frac{\alpha\left(e^{1/\tilde{\ell}} - 1\right)}{2\alpha + 1/\tilde{\ell}}. \tag{S229}$$

Indeed, since $\nu = \alpha\tilde{\ell}$, the previous expression can be written as

$$\phi(0) = e^{-\alpha}\left[1 + \mathrm{Pe}_{\mathrm{cue},0} B_{\tilde{\ell}}(\alpha) + O(\mathrm{Pe}_{\mathrm{cue},0}^2)\right]. \tag{S230}$$

Therefore,

$$\frac{1}{\phi(0)} = e^{\alpha}\left[1 - \mathrm{Pe}_{\mathrm{cue},0} B_{\tilde{\ell}}(\alpha) + O(\mathrm{Pe}_{\mathrm{cue},0}^2)\right], \tag{S231}$$

bioRxiv preprint doi: https://doi.org/10.64898/2026.09.16.750450; this version posted September 17, 2026. The copyright holder for this preprint (which was not certified by peer review) is the author/funder, who has granted bioRxiv a license to display the preprint in perpetuity. It is made available under a CC-BY 4.0 International license.

and the mean first-passage time becomes

$$\tilde{T}(0;\alpha) = \frac{e^{\alpha}-1}{\alpha^2} - \mathrm{Pe}_{\mathrm{cue},0}\frac{e^{\alpha}B_{\tilde{\ell}}(\alpha)}{\alpha^2} + O(\mathrm{Pe}_{\mathrm{cue},0}^2). \tag{S232}$$

The blind-search result corresponds to $\mathrm{Pe}_{\mathrm{cue},0} = 0$:

$$\tilde{T}_{\mathrm{blind}}(\alpha) = \frac{e^{\alpha}-1}{\alpha^2}. \tag{S233}$$

The blind-search optimum is obtained by minimizing $\tilde{T}_{\mathrm{blind}}(\alpha)$ with respect to α. We denote the minimizing value by α_0, defined by

$$\left.\frac{d\tilde{T}_{\mathrm{blind}}}{d\alpha}\right|_{\alpha=\alpha_0} = 0. \tag{S234}$$

This gives

$$e^{\alpha_0}(\alpha_0-2)+2=0, \tag{S235}$$

with

$$\alpha_0 \simeq 1.5936, \qquad \tilde{T}^*_{R,\mathrm{blind}} = \frac{1}{\alpha_0^2} \simeq 0.394. \tag{S236}$$

We now ask how the optimal reset rate changes when $\mathrm{Pe}_{\mathrm{cue},0}$ is increased slightly away from zero. We write

$$\alpha^*(\mathrm{Pe}_{\mathrm{cue},0}) = \alpha_0 + \mathrm{Pe}_{\mathrm{cue},0}\alpha_1 + O(\mathrm{Pe}_{\mathrm{cue},0}^2). \tag{S237}$$

Using the expansion in eqn. S232, the sign of the first correction α_1 is controlled by

$$\mathrm{sign}(\alpha_1) = \mathrm{sign}\left[1 - \frac{1}{\alpha_0} - \frac{2}{2\alpha_0 + 1/\tilde{\ell}}\right]. \tag{S238}$$

Since

$$\tilde{T}^*_R = \frac{1}{(\alpha^*)^2}, \tag{S239}$$

the initial change in $\tilde{T}^*_R$ has the opposite sign to the initial change in α^*.

The sign change occurs when the bracket in eqn. S238 vanishes. This gives

$$\tilde{\ell}_{\mathrm{lb}} = \frac{\alpha_0 - 1}{2\alpha_0(2-\alpha_0)} \simeq 0.46. \tag{S240}$$

Thus, to leading order at small $\mathrm{Pe}_{\mathrm{cue},0}$,

$$\tilde{\ell} > \tilde{\ell}_{\mathrm{lb}} \implies \tilde{T}^*_R \text{ initially increases with } \mathrm{Pe}_{\mathrm{cue},0}, \tag{S241}$$

whereas

$$\tilde{\ell} < \tilde{\ell}_{\mathrm{lb}} \implies \tilde{T}^*_R \text{ initially decreases with } \mathrm{Pe}_{\mathrm{cue},0}. \tag{S242}$$

This explains the different behavior of the line cuts in Fig. S25(A). For broader cues, $\tilde{\ell} > \tilde{\ell}_{\mathrm{lb}}$, increasing the cue strength initially favors less frequent resetting. For more localized cues, $\tilde{\ell} < \tilde{\ell}_{\mathrm{lb}}$, increasing the cue strength initially favors more frequent resetting, producing a dip in $\tilde{T}^*_R$.

The value $\tilde{\ell}_{\mathrm{lb}} \simeq 0.46$ therefore provides an analytic lower bound on the threshold cue length shown in Fig. S25(B). Near the actual threshold, the optimal reset time can first increase before dipping below the blind-search value at finite cue strength, so the weak-cue calculation does not determine the exact threshold.

bioRxiv preprint doi: https://doi.org/10.64898/2026.09.16.750450; this version posted September 17, 2026. The copyright holder for this preprint (which was not certified by peer review) is the author/funder, who has granted bioRxiv a license to display the preprint in perpetuity. It is made available under a CC-BY 4.0 International license.

I: The Near-Target Limit for a Single Checkpoint

The § IV of the main text ("Random Walk with Resets to Checkpoints") reported that the speedup in the time to reach a target with checkpoints is diluted from an exponential benefit to a linear benefit in x_T/λ_D, where x_T is the target location and λ_D is the typical diffusive lengthscale of the walk. Here we justify this claim.

For $x_T \ll \lambda_D$, we expand eqn. (126) to leading order in $\Lambda = x_T/\lambda_D$,

$$\frac{\langle T_{\text{one checkpoint}}\rangle}{\langle T_{\text{no checkpoint}}\rangle} = \frac{2\left(e^{\Lambda/2}-1\right)}{e^{\Lambda}-1}, \tag{S243}$$

using

$$e^{\Lambda} = 1+\Lambda+\frac{\Lambda^2}{2}+O(\Lambda^3), \qquad e^{\Lambda/2} = 1+\frac{\Lambda}{2}+\frac{\Lambda^2}{8}+O(\Lambda^3). \tag{S244}$$

It gives

$$\frac{\langle T_{\text{one checkpoint}}\rangle}{\langle T_{\text{no checkpoint}}\rangle} = \frac{1+\Lambda/4+O(\Lambda^2)}{1+\Lambda/2+O(\Lambda^2)}. \tag{S245}$$

Expanding the denominator to first order,

$$\frac{\langle T_{\text{one checkpoint}}\rangle}{\langle T_{\text{no checkpoint}}\rangle} = \left(1+\frac{\Lambda}{4}\right)\left(1-\frac{\Lambda}{2}\right)+O(\Lambda^2) = 1-\frac{\Lambda}{4}+O(\Lambda^2). \tag{S246}$$

Therefore,

$$\frac{\langle T_{\text{one checkpoint}}\rangle}{\langle T_{\text{no checkpoint}}\rangle} = 1-\frac{x_T}{4\lambda_D}+O\left(\frac{x_T^2}{\lambda_D^2}\right), \qquad x_T \ll \lambda_D. \tag{S247}$$

Thus, in the short-target-distance regime, the gain from introducing a checkpoint is linear in x_T/λ_D rather than exponential.

J: Parallel search with multiple independent searchers

1. Mean earliest-arrival time from the collective survival probability

Here we explain the observation in the main text, § V 1, that the integral of the survival probability $S(t)$ yields the mean first passage time $\langle T\rangle$, including for multiple independent searchers.

For N independent searchers, the probability that none of the searchers has reached the target by time t is

$$\mathbb{P}(\tau_{\min} > t) = S(t)^N, \tag{S248}$$

where $S(t)$ is the survival probability of a single searcher and

$$\tau_{\min} = \min(\tau_1,\ldots,\tau_N), \tag{S249}$$

and τ_i is the first passage time of the ith searcher. The corresponding probability density for the earliest arrival is

$$f_{\min}(t) = -\frac{d}{dt}\mathbb{P}(\tau_{\min} > t) = -\frac{d}{dt}S(t)^N. \tag{S250}$$

The mean earliest-arrival time is then

$$T_{\min} \equiv \langle \tau_{\min}\rangle = \int_0^\infty t\, f_{\min}(t)\, dt. \tag{S251}$$

Substituting the expression for $f_{\min}(t)$ gives

$$T_{\min} = -\int_0^\infty t\, \frac{d}{dt}S(t)^N\, dt. \tag{S252}$$

bioRxiv preprint doi: https://doi.org/10.64898/2026.09.16.750450; this version posted September 17, 2026. The copyright holder for this preprint (which was not certified by peer review) is the author/funder, who has granted bioRxiv a license to display the preprint in perpetuity. It is made available under a CC-BY 4.0 International license.

Integrating by parts,

$$T_{\min} = -\left[tS(t)^N\right]_0^\infty + \int_0^\infty S(t)^N \, dt. \tag{S253}$$

The boundary term vanishes at $t = 0$ because of the prefactor t, and at $t \to \infty$ because the probability of surviving without reaching the absorbing target tends to zero. We obtain

$$T_{\min} = \int_0^\infty S(t)^N \, dt. \tag{S254}$$

The mean earliest-arrival time for N independent searchers is consequently determined entirely by the single-searcher survival probability $S(t)$.

2. Derivation of the backward equation for the survival probability

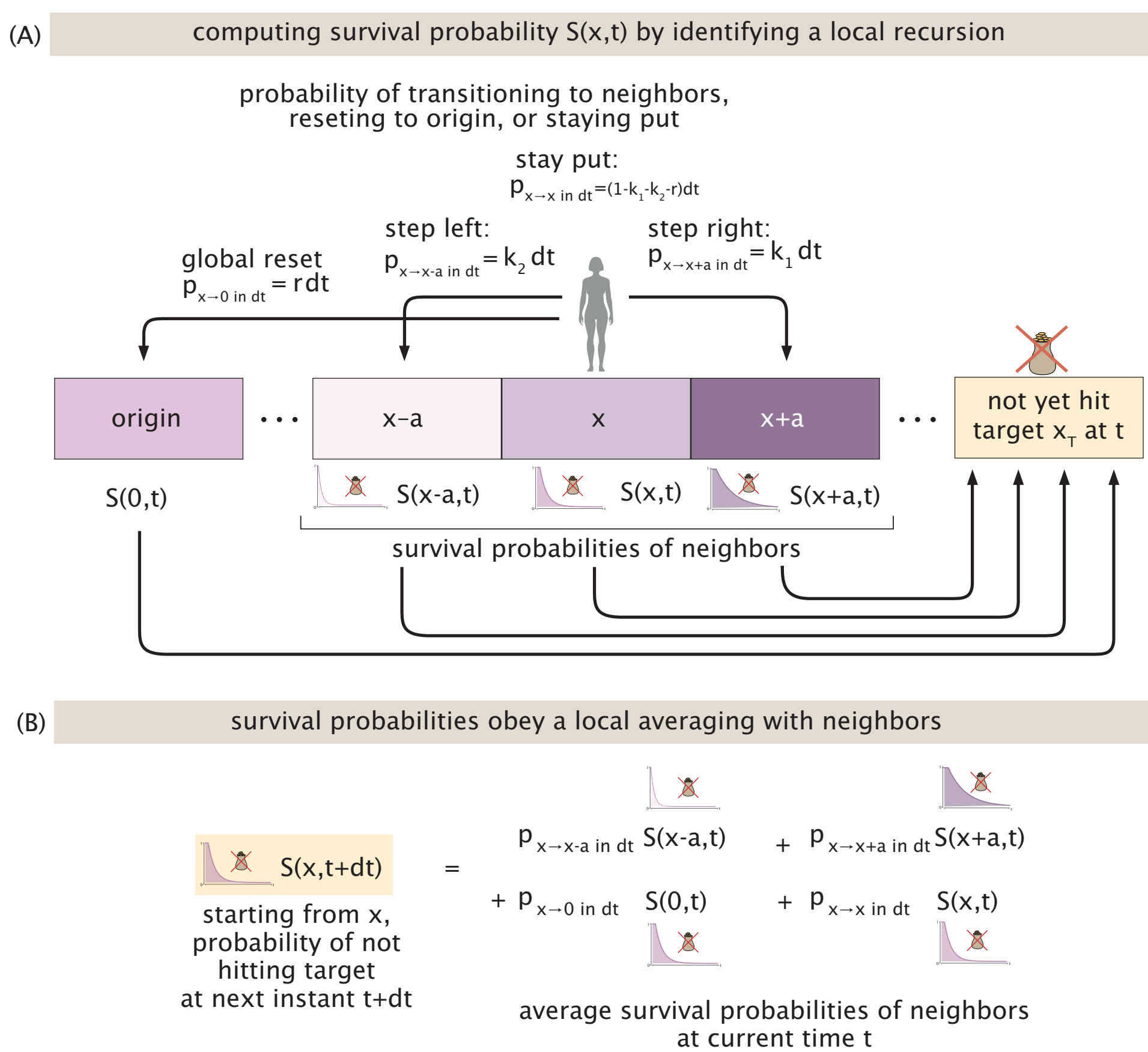

FIG. S26: Origins of the local averaging property of a survival probability $S(x,t)$ that a seeker has not hit a target at time t. (A) The updated survival probability $S(x,t+dt)$ follows from available jumps in the time interval dt from neighboring positions (weighted by the probabilities of such jumps), with their own survival probabilities at the previous time t. (Each site's purple shading reflects its local survival probability, stressing a coherence with those of neighboring sites.) (B) This averaging conditioned on possible jumps yields a self-consistent expression for the survival probability.

In §V of the main text, we study statistical properties of arrivals by multiple searchers. A key tool along the way is the survival probability $S(x,t)$ of a search not yet having found the target by time t after having started at position x. Here we derive eqn. 145 for this position-dependent survival probability $S(x,t)$ introduced in the main text, § V 1. We use the same backward-equation approach employed previously for the mean first-passage time, now applying the local averaging argument to survival probabilities rather than times. We consider a continuous-time random walk on a lattice with spacing a. The walker steps to the right with rate $k + \Delta k$, to the left with rate $k - \Delta k$, and resets to the origin with rate r.

bioRxiv preprint doi: https://doi.org/10.64898/2026.09.16.750450; this version posted September 17, 2026. The copyright holder for this preprint (which was not certified by peer review) is the author/funder, who has granted bioRxiv a license to display the preprint in perpetuity. It is made available under a CC-BY 4.0 International license.

The probability $S(x,t+dt)$ of surviving for a total duration $t+dt$ is obtained by averaging over the possible events during the first interval dt. Because the process is Markovian, after this first event the probability of surviving for the remaining time t depends only on the new position. Therefore,

$$\begin{aligned} S(x,t+dt) = (k+\Delta k)dt\,S(x+a,t) + (k-\Delta k)dt\,S(x-a,t) \\ + r dt\,S(0,t) + [1-(2k+r)dt]\,S(x,t). \end{aligned} \tag{S255}$$

Subtracting $S(x,t)$, dividing by dt, and taking the limit $dt \to 0$ gives

$$\begin{aligned} \frac{\partial S(x,t)}{\partial t} = (k+\Delta k)\,[S(x+a,t) - S(x,t)] \\ + (k-\Delta k)\,[S(x-a,t) - S(x,t)] - rS(x,t) + rS(0,t). \end{aligned} \tag{S256}$$

Expanding $S(x \pm a,t)$ to second order in a gives

$$\frac{\partial S(x,t)}{\partial t} = 2a\Delta k\,\frac{\partial S(x,t)}{\partial x} + a^2 k\,\frac{\partial^2 S(x,t)}{\partial x^2} - rS(x,t) + rS(0,t). \tag{S257}$$

Using

$$v = 2a\Delta k, \qquad D = a^2 k, \tag{S258}$$

we recover

$$\frac{\partial S(x,t)}{\partial t} = v\,\frac{\partial S(x,t)}{\partial x} + D\,\frac{\partial^2 S(x,t)}{\partial x^2} - rS(x,t) + rS(0,t), \tag{S259}$$

which is eqn. 145 of the main text.

3. Characteristic earliest-arrival time and relation to the mean

In § V 1 of the main text, we discussed for instance the heuristic that the required number of searchers N for earliest arrivals satisfies $NF(t) = O(1)$, where $F(t)$ is the probability that a single searcher has arrived by time t. Here we derive this statement, its consequences, and some related results.

To extract the large-N behavior of the mean earliest-arrival time, it is useful to introduce an auxiliary characteristic timescale. We define the cumulative first-passage probability of a single searcher as

$$F(t) = 1 - S(t) = \mathbb{P}(\tau_i \le t). \tag{S260}$$

For N independent searchers, the collective survival probability is

$$\mathbb{P}(\tau_{\min} > t) = [1 - F(t)]^N. \tag{S261}$$

We define a characteristic first-arrival time $t_N(q)$ by fixing the collective survival probability to some value $0 < q < 1$,

$$\mathbb{P}(\tau_{\min} > t_N) = [1 - F(t_N)]^N = q. \tag{S262}$$

Solving for the corresponding single-searcher arrival probability gives

$$F(t_N) = 1 - q^{1/N}. \tag{S263}$$

For large N,

$$q^{1/N} \approx \exp\left(\frac{\ln q}{N}\right) \approx 1 + \frac{\ln q}{N} + O\left(\frac{1}{N^2}\right), \tag{S264}$$

and therefore

$$NF(t_N) = -\ln q + O\left(\frac{1}{N}\right). \tag{S265}$$

bioRxiv preprint doi: https://doi.org/10.64898/2026.09.16.750450; this version posted September 17, 2026. The copyright holder for this preprint (which was not certified by peer review) is the author/funder, who has granted bioRxiv a license to display the preprint in perpetuity. It is made available under a CC-BY 4.0 International license.

Hence any fixed choice of $0 < q < 1$,

$$NF(t_N) = O(1), \qquad F(t_N) = O\left(\frac{1}{N}\right). \tag{S266}$$

This result can also be obtained directly from the collective survival probability. For $F(t) \ll 1$,

$$\mathbb{P}(\tau_{\min} > t) = \exp\left[N \ln(1 - F(t))\right] \tag{S267}$$

$$= \exp\left[-NF(t) - \frac{N}{2}F(t)^2 + O\left(NF(t)^3\right)\right]. \tag{S268}$$

On the earliest-arrival timescale, $F(t) = O(1/N)$, so all terms beyond $NF(t)$ vanish as $N \to \infty$. Therefore,

$$\mathbb{P}(\tau_{\min} > t) \sim e^{-NF(t)}. \tag{S269}$$

For convenience, we choose

$$q = e^{-1}, \tag{S270}$$

for which

$$NF(t_N) \sim 1. \tag{S271}$$

With this convention,

$$\mathbb{P}(\tau_{\min} \leq t_N) \sim 1 - e^{-1} \simeq 0.63. \tag{S272}$$

The particular value of q is not important for the leading asymptotic behavior; this choice simply provides a convenient reference timescale.

Although t_N is a characteristic quantile rather than the mean earliest-arrival time, the two become asymptotically equivalent as $N \to \infty$. To show this, we use the short-time first-passage probability derived in Appendix §J 4,

$$F(t) \sim \frac{2\sqrt{Dt}}{x_T\sqrt{\pi}} \exp\left[-\frac{x_T^2}{4Dt} + \frac{vx_T}{2D}\right]. \tag{S273}$$

It is convenient to introduce

$$y = \frac{x_T^2}{4Dt}, \qquad y_N = \frac{x_T^2}{4Dt_N}. \tag{S274}$$

Since $NF(t_N) \sim 1$, times close to t_N may be written as

$$y = y_N + z. \tag{S275}$$

Using the short-time form above then gives, to leading order,

$$NF(t) \sim e^{-z}. \tag{S276}$$

Together with eqn. S269, this gives

$$\mathbb{P}(\tau_{\min} > t) \sim \exp\left[-e^{-z}\right]. \tag{S277}$$

Equivalently, the rescaled variable z follows the standard Gumbel distribution in the large-N limit, whose mean is the Euler–Mascheroni constant γ_E.

Since

$$t = \frac{x_T^2}{4D(y_N + z)} = t_N\left[1 - \frac{z}{y_N} + O\left(\frac{1}{y_N^2}\right)\right], \tag{S278}$$

averaging over the Gumbel fluctuations gives

$$T_{\min} = \langle \tau_{\min} \rangle = t_N\left[1 - \frac{\gamma_E}{y_N} + O\left(\frac{1}{y_N^2}\right)\right]. \tag{S279}$$

Because $y_N \sim \ln N$,

$$\frac{T_{\min}}{t_N} \longrightarrow 1, \qquad N \to \infty. \tag{S280}$$

Thus the auxiliary characteristic time t_N and the mean earliest-arrival time $T_{\min}$ have the same leading large-N behavior. The characteristic time is used only as an intermediate tool for extracting the extreme-value asymptotics; the physical quantity considered in the main text remains $T_{\min}$.

bioRxiv preprint doi: https://doi.org/10.64898/2026.09.16.750450; this version posted September 17, 2026. The copyright holder for this preprint (which was not certified by peer review) is the author/funder, who has granted bioRxiv a license to display the preprint in perpetuity. It is made available under a CC-BY 4.0 International license.

4. Short-time and large-N asymptotics of the earliest-arrival time

Here we derive the large-N behavior of the characteristic earliest-arrival time introduced in the main text. In particular, we determine the short-time behavior of the single-searcher cumulative first-passage probability $F(t)$ and use it to obtain both the leading large-N scaling of t_N and the first drift-dependent correction.

For large N, the earliest successful trajectory occurs at progressively shorter times. We therefore begin by determining the short-time behavior of the single-searcher arrival probability. In the absence of resetting, the first-passage density for a searcher starting at the origin, undergoing diffusion with coefficient D and constant drift v toward a target at $x_T > 0$, is

$$f_0(t) = \frac{x_T}{\sqrt{4\pi D t^3}} \exp\left[-\frac{(x_T - vt)^2}{4Dt}\right]. \tag{S281}$$

For this arrival to occur before the first reset, the searcher must also experience no reset during the interval $[0,t]$. Since resets occur as a Poisson process with rate r, the cumulative probability of reaching the target by time t without having reset is

$$F_{\mathrm{nr}}(t) = \int_0^t e^{-rs} f_0(s)\, ds. \tag{S282}$$

Expanding the exponent in the integrand gives

$$-\frac{(x_T - vs)^2}{4Ds} - rs = -\frac{x_T^2}{4Ds} + \frac{v x_T}{2D} - \left(\frac{v^2}{4D} + r\right) s. \tag{S283}$$

We define

$$A = \frac{x_T^2}{4D}, \qquad B = \frac{v^2}{4D} + r, \qquad c = \frac{v x_T}{2D}. \tag{S284}$$

Equation S282 can then be written as

$$F_{\mathrm{nr}}(t) = \frac{x_T}{\sqrt{4\pi D}} e^c \int_0^t s^{-3/2} e^{-A/s - Bs}\, ds. \tag{S285}$$

To determine the small-t equivalent of the remaining integral, define

$$W(t) = \int_0^t s^{-3/2} e^{-A/s - Bs}\, ds, \tag{S286}$$

and consider

$$g(t) = \frac{\sqrt{t}}{A} e^{-A/t}. \tag{S287}$$

Both $W(t)$ and $g(t)$ vanish as $t \to 0^+$. We may therefore use L'Hôpital's rule: when both the numerator and denominator of a ratio vanish in the limit, the limit of their ratio can be obtained from the ratio of their derivatives, provided the latter limit exists. Here,

$$W'(t) = t^{-3/2} e^{-A/t - Bt}, \tag{S288}$$

while

$$g'(t) = t^{-3/2} e^{-A/t} \left(1 + \frac{t}{2A}\right). \tag{S289}$$

It follows that

$$\lim_{t\to 0^+} \frac{W(t)}{g(t)} = \lim_{t\to 0^+} \frac{W'(t)}{g'(t)} \tag{S290}$$

$$= \lim_{t\to 0^+} \frac{e^{-Bt}}{1 + t/(2A)} \tag{S291}$$

$$= 1. \tag{S292}$$

bioRxiv preprint doi: https://doi.org/10.64898/2026.09.16.750450; this version posted September 17, 2026. The copyright holder for this preprint (which was not certified by peer review) is the author/funder, who has granted bioRxiv a license to display the preprint in perpetuity. It is made available under a CC-BY 4.0 International license.

Hence

$$W(t) \sim \frac{\sqrt{t}}{A} e^{-A/t}, \qquad t \to 0^+. \tag{S293}$$

Substituting this result into eqn. S285 gives

$$F_{\mathrm{nr}}(t) \sim \frac{2\sqrt{Dt}}{x_T\sqrt{\pi}} \exp\left[-\frac{x_T^2}{4Dt} + \frac{vx_T}{2D}\right], \qquad t \to 0^+. \tag{S294}$$

We next verify that the full first-passage probability $F(t)$, including trajectories that may reset, has the same leading short-time behavior. Conditioning on the time τ of the first reset gives

$$F(t) = F_{\mathrm{nr}}(t) + \int_0^t re^{-r\tau} S_0(\tau) F(t-\tau)\, d\tau, \tag{S295}$$

where $S_0(\tau)$ is the survival probability in the absence of resetting. Since $S_0(\tau) \leq 1$ and $F(t-\tau) \leq F(t)$,

$$0 \leq F(t) - F_{\mathrm{nr}}(t) \leq \left(1 - e^{-rt}\right) F(t). \tag{S296}$$

This implies

$$F_{\mathrm{nr}}(t) \leq F(t) \leq e^{rt} F_{\mathrm{nr}}(t). \tag{S297}$$

Since $e^{rt} \to 1$ as $t \to 0^+$,

$$F(t) \sim F_{\mathrm{nr}}(t). \tag{S298}$$

The full single-searcher arrival probability therefore satisfies

$$F(t) \sim \frac{2\sqrt{Dt}}{x_T\sqrt{\pi}} \exp\left[-\frac{x_T^2}{4Dt} + \frac{vx_T}{2D}\right], \qquad t \to 0^+. \tag{S299}$$

We now use this short-time form to determine the large-N behavior of the characteristic earliest-arrival time. For the convenient choice $q = e^{-1}$ introduced in the main text,

$$NF(t_N) \sim 1. \tag{S300}$$

Substituting eqn. S299 gives

$$N\frac{2\sqrt{Dt_N}}{x_T\sqrt{\pi}} \exp\left[-\frac{x_T^2}{4Dt_N} + \frac{vx_T}{2D}\right] \sim 1. \tag{S301}$$

Using A and c defined in eqn. S284, we introduce

$$y_N = \frac{A}{t_N}. \tag{S302}$$

Since $t_N = A/y_N$ and

$$\frac{2\sqrt{DA}}{x_T} = 1, \tag{S303}$$

eqn. S301 becomes

$$\frac{N}{\sqrt{\pi y_N}} e^{-y_N + c} \sim 1. \tag{S304}$$

Taking the logarithm gives

$$y_N + \frac{1}{2}\ln y_N = \ln N + c - \frac{1}{2}\ln \pi + o(1). \tag{S305}$$

bioRxiv preprint doi: https://doi.org/10.64898/2026.09.16.750450; this version posted September 17, 2026. The copyright holder for this preprint (which was not certified by peer review) is the author/funder, who has granted bioRxiv a license to display the preprint in perpetuity. It is made available under a CC-BY 4.0 International license.

At leading order,

$$y_N \sim \ln N, \tag{S306}$$

and hence

$$t_N \sim \frac{x_T^2}{4D\ln N}, \qquad N \to \infty. \tag{S307}$$

To obtain the first correction, we write

$$y_N = \ln N + \delta_N, \tag{S308}$$

where $\delta_N / \ln N \to 0$. Substituting this form into eqn. S305 gives

$$\ln N + \delta_N + \frac{1}{2}\ln(\ln N + \delta_N) = \ln N + c - \frac{1}{2}\ln\pi + o(1). \tag{S309}$$

Since

$$\ln(\ln N + \delta_N) = \ln\ln N + o(1), \tag{S310}$$

we find

$$\delta_N = -\frac{1}{2}\ln\ln N + c - \frac{1}{2}\ln\pi + o(1). \tag{S311}$$

Consequently,

$$y_N = \ln N - \frac{1}{2}\ln\ln N + \frac{vx_T}{2D} - \frac{1}{2}\ln\pi + o(1), \tag{S312}$$

and

$$t_N = \frac{x_T^2}{4D}\left[\ln N - \frac{1}{2}\ln\ln N + \frac{vx_T}{2D} - \frac{1}{2}\ln\pi + o(1)\right]^{-1}. \tag{S313}$$

Expanding around the leading large-N result gives the equivalent form

$$t_N = \frac{x_T^2}{4D\ln N}\left[1 + \frac{\frac{1}{2}\ln\ln N + \frac{1}{2}\ln\pi - \frac{vx_T}{2D}}{\ln N} + o\left(\frac{1}{\ln N}\right)\right]. \tag{S314}$$

We finally convert this characteristic timescale into the mean earliest-arrival time used in the main text. From Appendix §J 3,

$$T_{\min} = t_N\left[1 - \frac{\gamma_E}{y_N} + O\left(\frac{1}{y_N^2}\right)\right], \tag{S315}$$

where

$$y_N = \frac{x_T^2}{4Dt_N} \sim \ln N. \tag{S316}$$

Using eqn. S314 therefore gives

$$T_{\min} = \frac{x_T^2}{4D\ln N} + \frac{x_T^2}{8D}\frac{\ln\ln N}{(\ln N)^2} + \frac{x_T^2}{4D(\ln N)^2}\left[\frac{1}{2}\ln\pi - \gamma_E - \frac{vx_T}{2D}\right] + o\left(\frac{1}{(\ln N)^2}\right). \tag{S317}$$

This gives the decomposition used in the main text,

$$T_{\min} = T_{\min}^{(0)} + T_{\min}^{(1)} + T_{\min}^{(2)} + \cdots, \tag{S318}$$

with

$$T_{\min}^{(0)} = \frac{x_T^2}{4D\ln N}, \qquad T_{\min}^{(1)} = \frac{x_T^2}{8D}\frac{\ln\ln N}{(\ln N)^2}, \tag{S319}$$

bioRxiv preprint doi: https://doi.org/10.64898/2026.09.16.750450; this version posted September 17, 2026. The copyright holder for this preprint (which was not certified by peer review) is the author/funder, who has granted bioRxiv a license to display the preprint in perpetuity. It is made available under a CC-BY 4.0 International license.

and

$$T_{\min}^{(2)} = \frac{x_T^2}{4D(\ln N)^2}\left[\frac{1}{2}\ln\pi - \gamma_E - \frac{vx_T}{2D}\right]. \tag{S320}$$

The leading behavior is therefore

$$T_{\min} \sim \frac{x_T^2}{4D\ln N}, \qquad N \to \infty. \tag{S321}$$

The first correction is independent of drift, while drift first appears in $T_{\min}^{(2)}$. Resetting does not appear at this order because the winning trajectory occurs on a timescale much shorter than the mean reset time.

5. Approach to the reset-independent large-N regime

We now derive the characteristic population size N_c introduced in the main text. For a large population of independent searchers, the first arrival occurs when

$$NF(t) = O(1). \tag{S322}$$

We define N_c by evaluating this condition at a time $t = \varepsilon/r$, where $0 < \varepsilon \ll 1$. At this time, the first arrival occurs on a timescale much shorter than the mean reset time $1/r$, so that resetting has little opportunity to influence the winning trajectory.

Using the short-time result

$$F(t) \sim \frac{2\sqrt{Dt}}{x_T\sqrt{\pi}}\exp\left[-\frac{x_T^2}{4Dt} + \frac{vx_T}{2D}\right], \tag{S323}$$

we obtain

$$F\left(\frac{\varepsilon}{r}\right) \sim \sqrt{\frac{\varepsilon}{\pi\rho}}\exp\left[-\frac{\rho}{\varepsilon} + \frac{vx_T}{2D}\right], \tag{S324}$$

where

$$\rho = \frac{rx_T^2}{4D}. \tag{S325}$$

The large-N condition

$$N_c F\left(\frac{\varepsilon}{r}\right) \sim 1 \tag{S326}$$

therefore gives

$$N_c(\varepsilon) \sim \sqrt{\frac{\pi\rho}{\varepsilon}}\exp\left[\frac{\rho}{\varepsilon} - \frac{vx_T}{2D}\right], \qquad \varepsilon \to 0. \tag{S327}$$

The dominant dependence is exponential in ρ/ε. Increasing the reset rate therefore shifts the onset of the reset-independent regime to larger populations, whereas positive drift toward the target lowers N_c.

For the numerical comparison in Fig. 30, we determine N_c numerically from

$$T_{\min}(N_c) = \frac{\varepsilon}{r}. \tag{S328}$$

Since the mean earliest-arrival time and the characteristic large-N first-arrival timescale are asymptotically equivalent, this numerical definition approaches the asymptotic prediction above when N_c is sufficiently large.

bioRxiv preprint doi: https://doi.org/10.64898/2026.09.16.750450; this version posted September 17, 2026. The copyright holder for this preprint (which was not certified by peer review) is the author/funder, who has granted bioRxiv a license to display the preprint in perpetuity. It is made available under a CC-BY 4.0 International license.

6. Optimal number of searchers in the presence of a resource cost

Parallel search reduces the mean time to first success, but deploying additional searchers may itself carry a cost, as discussed in the main text, §V 1. To illustrate this tradeoff, we assign an effective cost γ to each searcher, expressed in units of time, and consider

$$\mathscr{C}(N) = T_{\min}(N) + \gamma N. \tag{S329}$$

Using the leading large-N behavior

$$T_{\min}(N) \sim \frac{x_T^2}{4D\ln N}, \tag{S330}$$

we obtain

$$\mathscr{C}(N) \simeq \frac{x_T^2}{4D\ln N} + \gamma N. \tag{S331}$$

For the purpose of optimization, we treat N as a continuous variable. The derivative of eqn. S331 is

$$\frac{d\mathscr{C}}{dN} = -\frac{x_T^2}{4D}\frac{1}{N(\ln N)^2} + \gamma. \tag{S332}$$

The optimal population size N^* therefore satisfies

$$\gamma = \frac{x_T^2}{4D}\frac{1}{N^*(\ln N^*)^2}. \tag{S333}$$

The left-hand side is the fixed marginal cost of adding one searcher, while the right-hand side is the magnitude of the marginal reduction in the earliest-arrival time. The optimum is reached when these two contributions balance.

It is also possible to write the optimal number of searchers N^* explicitly. Defining

$$A = \frac{x_T^2}{4D}, \tag{S334}$$

eqn. S333 becomes

$$N^*(\ln N^*)^2 = \frac{A}{\gamma}. \tag{S335}$$

Writing

$$x = \ln N^*, \qquad N^* = e^x, \tag{S336}$$

gives

$$x^2 e^x = \frac{A}{\gamma}. \tag{S337}$$

Taking the positive square root,

$$x e^{x/2} = \sqrt{\frac{A}{\gamma}}. \tag{S338}$$

Introducing $u = x/2$ gives

$$u e^u = \frac{1}{2}\sqrt{\frac{A}{\gamma}}. \tag{S339}$$

The solution can therefore be expressed in terms of the Lambert W function, defined by $W(z)e^{W(z)} = z$,

$$x = 2W\left(\frac{1}{2}\sqrt{\frac{A}{\gamma}}\right). \tag{S340}$$

bioRxiv preprint doi: https://doi.org/10.64898/2026.09.16.750450; this version posted September 17, 2026. The copyright holder for this preprint (which was not certified by peer review) is the author/funder, who has granted bioRxiv a license to display the preprint in perpetuity. It is made available under a CC-BY 4.0 International license.

Hence

$$N^* = \exp\left[2W\left(\frac{1}{2}\sqrt{\frac{A}{\gamma}}\right)\right], \qquad A = \frac{x_T^2}{4D}. \tag{S341}$$

The second derivative,

$$\frac{d^2\mathscr{C}}{dN^2} = \frac{A}{N^2}\left[\frac{1}{(\ln N)^2} + \frac{2}{(\ln N)^3}\right], \tag{S342}$$

is positive for $N > 1$, confirming that this stationary point is a minimum within the continuous approximation. Since the actual population size is discrete, the physical optimum is obtained by comparing the neighboring integers around the value predicted by eqn. S341.

The origin of the finite optimum is the diminishing return of parallelization: the characteristic earliest-arrival time decreases only as $1/\ln N$, so the marginal improvement from each additional searcher scales as $1/[N(\ln N)^2]$, while the marginal cost remains equal to γ.

K: Additional results for parallel search

1. Large-N behavior of the Mth arrival

As discussed in the main text, §V 2, the Mth arrival occurs when the number of successful searchers becomes of order M. Since a single searcher has reached the target by time t with probability

$$F(t) = 1 - S(t), \tag{S343}$$

the expected number of successful searchers is $NF(t)$. For $N \gg M$ with M fixed, the characteristic time of the Mth arrival therefore satisfies

$$NF(t) = O(M), \tag{S344}$$

or equivalently

$$F(t) = O\left(\frac{M}{N}\right). \tag{S345}$$

For large N, this corresponds to the short-time tail of the single-searcher first-passage distribution. As derived above,

$$F(t) \sim \frac{2\sqrt{Dt}}{x_T\sqrt{\pi}}\exp\left[-\frac{x_T^2}{4Dt} + \frac{vx_T}{2D}\right]. \tag{S346}$$

Resetting does not appear at this order because these very early arrivals typically reach the target before a reset occurs.

To determine the leading dependence on N, we define

$$z = \frac{x_T^2}{4Dt}. \tag{S347}$$

The short-time first-passage probability can then be written as

$$F(t) \sim \frac{1}{\sqrt{\pi z}}\exp\left[-z + \frac{vx_T}{2D}\right]. \tag{S348}$$

Using $F(t) = O(M/N)$ and taking the logarithm gives

$$z + \frac{1}{2}\ln z = \ln\left(\frac{N}{M}\right) + O(1). \tag{S349}$$

For $N \gg M$, the logarithm on the right-hand side is large, while $\ln z$ and the remaining terms contribute only subleading corrections. Therefore,

$$z \sim \ln\left(\frac{N}{M}\right). \tag{S350}$$

bioRxiv preprint doi: https://doi.org/10.64898/2026.09.16.750450; this version posted September 17, 2026. The copyright holder for this preprint (which was not certified by peer review) is the author/funder, who has granted bioRxiv a license to display the preprint in perpetuity. It is made available under a CC-BY 4.0 International license.

Using $z = x_T^2/(4Dt)$ gives the leading large-N behavior

$$T_{M:N} \sim \frac{x_T^2}{4D\ln(N/M)}, \qquad N \to \infty \quad \text{with fixed } M. \tag{S351}$$

For $M = 1$, this reduces to the earliest-arrival result $T_{1:N} \sim x_T^2/(4D\ln N)$. More generally, the appearance of N/M has a simple interpretation: waiting for M successes among N searchers has, at leading order, the same short-time scaling as waiting for one success among roughly N/M searchers.

2. Approach to the reset-independent regime for the Mth arrival

We next generalize this criterion for the onset of the reset-independent regime to the Mth arrival. The basic idea is that resetting becomes irrelevant to the successful trajectory once the required number of arrivals typically occurs on a timescale much shorter than the reset time $1/r$.

As in the main text, we introduce a small dimensionless number ε and consider the time

$$t_\varepsilon = \frac{\varepsilon}{r}. \tag{S352}$$

For M successes to have occurred by this time, the expected number of successful searchers must be of order M,

$$N_c F(t_\varepsilon) = O(M). \tag{S353}$$

We define the characteristic population size N_c by the condition

$$N_c F(t_\varepsilon) \simeq M. \tag{S354}$$

Using the short-time form in eqn. S346, we have

$$F\left(\frac{\varepsilon}{r}\right) \sim \frac{2}{x_T\sqrt{\pi}}\sqrt{\frac{D\varepsilon}{r}}\exp\left[-\frac{r x_T^2}{4D\varepsilon} + \frac{v x_T}{2D}\right]. \tag{S355}$$

Introducing

$$\rho = \frac{r x_T^2}{4D}, \tag{S356}$$

this becomes

$$F\left(\frac{\varepsilon}{r}\right) \sim \sqrt{\frac{\varepsilon}{\pi\rho}}\exp\left[-\frac{\rho}{\varepsilon} + \frac{v x_T}{2D}\right]. \tag{S357}$$

Substituting eqn. S357 into eqn. S354 gives

$$N_c(M,\varepsilon) \sim M\sqrt{\frac{\pi\rho}{\varepsilon}}\exp\left[\frac{\rho}{\varepsilon} - \frac{v x_T}{2D}\right], \tag{S358}$$

which is eqn. 163 of the main text.

In particular,

$$N_c(M,\varepsilon) \sim M\, N_c(1,\varepsilon), \tag{S359}$$

so requiring M successful arrivals increases the characteristic number of searchers needed to outrun resetting approximately linearly with M. The precise numerical value of N_c depends on the operational criterion used to define when resetting has become negligible, but the linear dependence on M and the exponential dependence on ρ/ε follow directly from the short-time first-passage probability.

bioRxiv preprint doi: https://doi.org/10.64898/2026.09.16.750450; this version posted September 17, 2026. The copyright holder for this preprint (which was not certified by peer review) is the author/funder, who has granted bioRxiv a license to display the preprint in perpetuity. It is made available under a CC-BY 4.0 International license.

3. Long-time survival and the last arrival

To determine the behavior of the last arrival, we first need the long-time survival probability of a single searcher. We follow the approach of Pal *et al.*[20], who considered a drift-diffusion process with Poisson resetting in the presence of an absorbing boundary. Their absorbing boundary is placed at the origin; here, instead, the searcher resets to $x = 0$ and the absorbing target is located at $x = x_T$.

Let $S(x,t)$ denote the probability that a searcher starting at position x has not yet reached the target by time t. As derived above, it obeys the backward equation

$$\frac{\partial S}{\partial t} = D\frac{\partial^2 S}{\partial x^2} + v\frac{\partial S}{\partial x} - rS(x,t) + rS(0,t), \tag{S360}$$

with

$$S(x_T,t) = 0, \qquad S(x,0) = 1. \tag{S361}$$

Following Ref.[20], we take the Laplace transform in time,

$$\widetilde{S}(x,s) = \int_0^\infty e^{-st} S(x,t)\, dt. \tag{S362}$$

Equation S360 then becomes

$$D\frac{d^2\widetilde{S}}{dx^2} + v\frac{d\widetilde{S}}{dx} - (r+s)\widetilde{S}(x,s) = -1 - r\widetilde{S}(0,s). \tag{S363}$$

Solving this equation while requiring the solution to remain bounded away from the target and imposing $\widetilde{S}(x_T,s) = 0$ gives, for a searcher starting from the reset position,

$$\widetilde{S}(0,s) = \frac{1 - e^{-\kappa(s)x_T}}{s + re^{-\kappa(s)x_T}}, \tag{S364}$$

where

$$\kappa(s) = \frac{\sqrt{v^2 + 4D(r+s)} - v}{2D}. \tag{S365}$$

We now use this result to determine the long-time behavior. Recall that an exponential decay $e^{-\alpha t}$ has Laplace transform $1/(s+\alpha)$. We therefore look for the slowest exponential contribution to eqn. S364, which is set by a zero of its denominator. If we denote this value by s_*, it satisfies

$$s_* + r\exp\left[-\frac{x_T}{2D}\left(\sqrt{v^2 + 4D(r+s_*)} - v\right)\right] = 0. \tag{S366}$$

Since the second term is positive, any such solution necessarily has $s_* < 0$. We therefore write $s_* = -\alpha$, with $\alpha > 0$, giving

$$\alpha = r\exp\left[-\frac{x_T}{2D}\left(\sqrt{v^2 + 4D(r-\alpha)} - v\right)\right]. \tag{S367}$$

This is a transcendental equation for α, and in general does not admit a closed-form solution; instead, it implicitly determines the long-time decay rate from D, v, r, and x_T. The single-searcher survival probability therefore behaves at long times as

$$S(t) \sim Ce^{-\alpha t}, \qquad t \to \infty, \tag{S368}$$

where C is a constant. We can now return to the last of N independent searchers. The probability that all N searchers have arrived by time t is

$$\mathbb{P}(\tau_{N:N} \le t) = [1 - S(t)]^N. \tag{S369}$$

At the late times relevant for the final arrival, $S(t) \ll 1$, and therefore

$$[1 - S(t)]^N \simeq e^{-NS(t)}. \tag{S370}$$

bioRxiv preprint doi: https://doi.org/10.64898/2026.09.16.750450; this version posted September 17, 2026. The copyright holder for this preprint (which was not certified by peer review) is the author/funder, who has granted bioRxiv a license to display the preprint in perpetuity. It is made available under a CC-BY 4.0 International license.

The last arrival occurs when the expected number of searchers that remain unsuccessful becomes of order one,

$$NS(t) = O(1). \tag{S371}$$

Using the long-time form $S(t) \sim Ce^{-\alpha t}$ gives

$$NCe^{-\alpha t} = O(1), \tag{S372}$$

so that, to leading order for $N \gg 1$,

$$T_{N:N} \sim \frac{\ln N}{\alpha}. \tag{S373}$$

L: Derivation of the first-passage Brownian bridge statistics

In this appendix, we derive the conditional probability density, mean trajectory, and variance of the first-passage Brownian bridge, and analyze the monotonicity and maximal excursion of its mean trajectory.

1. Conditional probability density

We set $t_i = 0$ and $x_i = 0$, and introduce the variable

$$z = x_T - x, \tag{S374}$$

which represents the remaining distance to the target. In terms of z, the absorbing-boundary propagator becomes

$$p_{\text{abs}}(x_T - z, t \mid 0, 0; x_T) = \frac{1}{\sqrt{4\pi Dt}} \left[e^{-(z-x_T)^2/(4Dt)} - e^{-(z+x_T)^2/(4Dt)} \right]. \tag{S375}$$

The first-passage density in the denominator is

$$f_{\text{FP}}(t_f \mid 0, 0; x_T) = \frac{x_T}{\sqrt{4\pi D t_f^3}} e^{-x_T^2/(4Dt_f)}, \tag{S376}$$

while the numerator contains

$$f_{\text{FP}}(t_f - t \mid x_T - z, t; x_T) = \frac{z}{\sqrt{4\pi D(t_f - t)^3}} e^{-z^2/[4D(t_f - t)]}. \tag{S377}$$

Substituting these expressions into the conditional probability gives exponentials of the form

$$-\frac{(z \pm x_T)^2}{4Dt} - \frac{z^2}{4D(t_f - t)} + \frac{x_T^2}{4Dt_f}. \tag{S378}$$

Collecting powers of z and completing the square yields

$$-\frac{t_f}{4Dt(t_f - t)} \left(z \pm \frac{x_T(t_f - t)}{t_f} \right)^2. \tag{S379}$$

We therefore identify

$$\mu(t) = \frac{x_T(t_f - t)}{t_f}, \qquad \sigma(t)^2 = \frac{2Dt(t_f - t)}{t_f}. \tag{S380}$$

The probability density becomes

$$p_{\text{FPbb}}(x_T - z, t \mid 0, 0; x_T, t_f) = \frac{z}{\mu(t)\sqrt{2\pi\sigma(t)^2}} \left[e^{-\frac{(z-\mu(t))^2}{2\sigma(t)^2}} - e^{-\frac{(z+\mu(t))^2}{2\sigma(t)^2}} \right]. \tag{S381}$$

bioRxiv preprint doi: https://doi.org/10.64898/2026.09.16.750450; this version posted September 17, 2026. The copyright holder for this preprint (which was not certified by peer review) is the author/funder, who has granted bioRxiv a license to display the preprint in perpetuity. It is made available under a CC-BY 4.0 International license.

2. Mean and variance

The mean remaining distance to the target is

$$\langle z(t)\rangle = \int_0^\infty z\, p_{\mathrm{FPbb}}(x_T - z, t \mid 0,0; x_T, t_f)\, dz. \tag{S382}$$

Substituting the probability density gives

$$\langle z(t)\rangle = \frac{1}{\mu(t)} \int_0^\infty z^2 \left[\phi(z;\mu(t),\sigma(t)^2) - \phi(z;-\mu(t),\sigma(t)^2)\right] dz, \tag{S383}$$

where $\phi(z;\mu,\sigma^2)$ is the Gaussian density with mean μ and variance σ^2. Using the change of variables

$$u = \frac{z \mp \mu}{\sqrt{2}\sigma}, \tag{S384}$$

together with the definition of the error function,

$$\mathrm{erf}(x) = \frac{2}{\sqrt{\pi}} \int_0^x e^{-u^2}\, du, \tag{S385}$$

the Gaussian integrals can be evaluated explicitly. After expanding the square and integrating term by term, we obtain

$$\langle z(t)\rangle = \left(\mu(t) + \frac{\sigma(t)^2}{\mu(t)}\right) \mathrm{erf}\left(\frac{\mu(t)}{\sqrt{2}\sigma(t)}\right) + \sqrt{\frac{2}{\pi}}\sigma(t) e^{-\frac{\mu(t)^2}{2\sigma(t)^2}}. \tag{S386}$$

Since $z = x_T - x$, the average trajectory is

$$\langle x(t)\rangle = x_T - \langle z(t)\rangle. \tag{S387}$$

To compute the variance, we evaluate the second moment

$$\langle z^2(t)\rangle = \frac{1}{\mu(t)} \int_0^\infty z^3 \left[\phi(z;\mu(t),\sigma(t)^2) - \phi(z;-\mu(t),\sigma(t)^2)\right] dz. \tag{S388}$$

Using the same Gaussian integration procedure gives

$$\langle z^2(t)\rangle = \mu(t)^2 + 3\sigma(t)^2. \tag{S389}$$

The variance is therefore

$$\mathrm{Var}[z(t)] = \langle z^2(t)\rangle - \langle z(t)\rangle^2. \tag{S390}$$

Since $z = x_T - x$, we have $\mathrm{Var}[x(t)] = \mathrm{Var}[z(t)]$.

3. Monotonicity and maximal excursion

To understand the shape of the average trajectory, it is useful to write

$$\langle x(t)\rangle = x_T - \mu(t) h(y(t)), \tag{S391}$$

where

$$h(y) = (1+y^2)\,\mathrm{erf}\left(\frac{1}{\sqrt{2}y}\right) + \sqrt{\frac{2}{\pi}} y e^{-\frac{1}{2y^2}}, \tag{S392}$$

and

$$y(t) = \frac{\sigma(t)}{\mu(t)} = \alpha\sqrt{\frac{t}{t_f - t}}, \tag{S393}$$

bioRxiv preprint doi: https://doi.org/10.64898/2026.09.16.750450; this version posted September 17, 2026. The copyright holder for this preprint (which was not certified by peer review) is the author/funder, who has granted bioRxiv a license to display the preprint in perpetuity. It is made available under a CC-BY 4.0 International license.

with

$$\alpha = \frac{\sqrt{2Dt_f}}{x_T}. \tag{S394}$$

Differentiating the average trajectory gives

$$\frac{d}{dt}\langle x(t)\rangle = \frac{x_T}{t_f}s(y), \tag{S395}$$

where

$$s(y) = (1-\alpha^2)\,\mathrm{erf}\left(\frac{1}{\sqrt{2}y}\right) + \sqrt{\frac{2}{\pi}}ye^{-\frac{1}{2y^2}}. \tag{S396}$$

If $\alpha \leq 1$, then

$$s(y) > 0 \tag{S397}$$

for all $y > 0$, so the average trajectory is monotonic. If $\alpha > 1$, the sign of $s(y)$ changes during the trajectory, implying non-monotonic behavior.

The turning point is obtained from

$$s(y_*) = 0. \tag{S398}$$

For large α, the solution satisfies

$$y_* = \alpha - \frac{1}{3\alpha} + O\left(\frac{1}{\alpha^3}\right), \tag{S399}$$

so that

$$t_* \simeq \frac{t_f}{2}. \tag{S400}$$

To obtain this asymptotic form, we start from the equation defining y_*,

$$(\alpha^2-1)\,\mathrm{erf}\left(\frac{1}{\sqrt{2}y_*}\right) = \sqrt{\frac{2}{\pi}}y_*e^{-\frac{1}{2y_*^2}}. \tag{S401}$$

For large α, the solution has large y_*. We therefore define

$$u = \frac{1}{\sqrt{2}y_*}, \tag{S402}$$

so that $u \ll 1$. The equation becomes

$$\alpha^2 - 1 = \frac{e^{-u^2}}{\sqrt{\pi}u\,\mathrm{erf}(u)}. \tag{S403}$$

Using the small-u expansions

$$\mathrm{erf}(u) = \frac{2}{\sqrt{\pi}}\left(u - \frac{u^3}{3} + O(u^5)\right), \tag{S404}$$

and

$$e^{-u^2} = 1 - u^2 + O(u^4), \tag{S405}$$

we obtain

$$\alpha^2 - 1 = \frac{1-u^2+O(u^4)}{2u^2\left(1-\frac{u^2}{3}+O(u^4)\right)} \tag{S406}$$

$$= \frac{1}{2u^2}\left[1 - \frac{2u^2}{3} + O(u^4)\right] \tag{S407}$$

$$= \frac{1}{2u^2} - \frac{1}{3} + O(u^2). \tag{S408}$$

bioRxiv preprint doi: https://doi.org/10.64898/2026.09.16.750450; this version posted September 17, 2026. The copyright holder for this preprint (which was not certified by peer review) is the author/funder, who has granted bioRxiv a license to display the preprint in perpetuity. It is made available under a CC-BY 4.0 International license.

Since $1/(2u^2) = y_*^2$, this gives

$$\alpha^2 - 1 = y_*^2 - \frac{1}{3} + O\left(\frac{1}{y_*^2}\right). \tag{S409}$$

Therefore,

$$y_*^2 = \alpha^2 - \frac{2}{3} + O\left(\frac{1}{\alpha^2}\right). \tag{S410}$$

Taking the square root gives

$$y_* = \alpha\left[1 - \frac{2}{3\alpha^2} + O\left(\frac{1}{\alpha^4}\right)\right]^{1/2}. \tag{S411}$$

Expanding the square root finally yields

$$y_* = \alpha - \frac{1}{3\alpha} + O\left(\frac{1}{\alpha^3}\right). \tag{S412}$$

At the turning point, the average remaining distance to the target is

$$\langle z(t_*)\rangle = \mu(t_*)h(y_*). \tag{S413}$$

Using $t_* \simeq t_f/2$, we obtain

$$\mu(t_*) \simeq \frac{x_T}{2}. \tag{S414}$$

In the large-α limit,

$$h(\alpha) \simeq 2\sqrt{\frac{2}{\pi}}\alpha, \tag{S415}$$

so that

$$\langle z(t_*)\rangle \simeq x_T\sqrt{\frac{2}{\pi}}\alpha. \tag{S416}$$

Using

$$\alpha = \frac{\sqrt{2Dt_f}}{x_T}, \tag{S417}$$

we finally obtain

$$\langle z(t_*)\rangle \simeq 2\sqrt{\frac{Dt_f}{\pi}}. \tag{S418}$$

Thus, in the regime $\sqrt{2Dt_f} \gg x_T$, the maximal excursion is controlled entirely by the diffusive length scale $\sqrt{Dt_f}$.

M: First-passage Brownian bridges with stochastic resetting

1. Conditional probability density and local transition kernel

We now generalize the framework developed above to diffusive processes with stochastic resetting. While Brownian bridges with resetting have previously been studied[7], to our knowledge the corresponding first-passage Brownian bridge with stochastic resetting has not previously been characterized. Here we derive its conditioned dynamics and examine the organization of successful trajectories into reset-terminated failed excursions followed by a final successful first-passage event. We consider a Brownian particle that diffuses with diffusion coefficient D and resets to the origin with Poissonian rate r, with the initial position

bioRxiv preprint doi: https://doi.org/10.64898/2026.09.16.750450; this version posted September 17, 2026. The copyright holder for this preprint (which was not certified by peer review) is the author/funder, who has granted bioRxiv a license to display the preprint in perpetuity. It is made available under a CC-BY 4.0 International license.

taken to be $x_i = 0$. During a small time interval Δt, the particle either diffuses normally with probability $1 - r\Delta t$, or resets to the origin with probability $r\Delta t$.

We first ask for the conditional probability density of finding the particle at position x at time t, given that the trajectory first reaches the target position x_T at the prescribed final time t_f. We write

$$p^{(r)}_{\text{FPbb}}(x,t \mid x_i,t_i;x_T,t_f). \tag{S419}$$

Using Bayes' theorem and the Markov property, we get

$$p^{(r)}_{\text{FPbb}}(x,t \mid x_i,t_i;x_T,t_f) = \frac{p^{(r)}_{\text{surv}}(x,t \mid x_i,t_i;x_T) f^{(r)}_{\text{FP}}(t_f - t \mid x;x_T)}{f^{(r)}_{\text{FP}}(t_f - t_i \mid x_i;x_T)}, \tag{S420}$$

for $x < x_T$, where $f^{(r)}_{\text{FP}}(T \mid x;x_T)$ denotes the first-passage-time density to x_T for a particle starting at x, with all resets returning the particle to the origin.

Because the trajectory is conditioned on first arrival at x_T at time t_f, it must not have reached x_T before the intermediate time t. Therefore, the appropriate probability density for the first part of the trajectory is the reset survival propagator,

$$p^{(r)}_{\text{surv}}(x,t \mid x_i,t_i;x_T), \tag{S421}$$

which gives the probability density that the particle is at position x at time t, having started from x_i at time t_i, while remaining below the target position x_T up to time t in the presence of resetting. This quantity satisfies the renewal equation

$$\begin{aligned} p^{(r)}_{\text{surv}}(x,t \mid x_i,t_i;x_T) &= e^{-r(t-t_i)} p_{\text{abs}}(x,t \mid x_i,t_i;x_T) \\ &\quad + r\int_{t_i}^{t} e^{-r(\tau-t_i)} S_0(\tau - t_i \mid x_i;x_T)\, p^{(r)}_{\text{surv}}(x,t \mid 0,\tau;x_T)\, d\tau, \end{aligned} \tag{S422}$$

where p_{abs} is the absorbing propagator without resetting and $S_0(\tau - t_i \mid x_i;x_T)$ is the corresponding no-reset survival probability.

This expression is the reset analog of the first-passage Brownian bridge density. The numerator counts all reset-diffusion microtrajectories that start at x_i at time t_i, survive below the target up to time t, pass through position x at time t, and then first reach x_T exactly at time t_f. The denominator counts all reset-diffusion microtrajectories that start at x_i at time t_i and first reach x_T at time t_f. The conditional probability is therefore the ratio between these two ensembles.

The reset-modified first-passage density $f^{(r)}_{\text{FP}}$ can be defined through a renewal equation. Let $S_r(T \mid x;x_T)$ denote the survival probability over a time T for a particle starting at x and resetting to the origin, and let $S_0(T \mid x;x_T)$ denote the corresponding survival probability without resetting. A trajectory that survives until time T either experiences no reset during the entire interval, or experiences a first reset at time τ and then starts the process again from the origin. This gives

$$S_r(T \mid x;x_T) = e^{-rT} S_0(T \mid x;x_T) + r\int_0^T e^{-r\tau} S_0(\tau \mid x;x_T) S_r(T-\tau \mid 0;x_T)\, d\tau. \tag{S423}$$

bioRxiv preprint doi: https://doi.org/10.64898/2026.09.16.750450; this version posted September 17, 2026. The copyright holder for this preprint (which was not certified by peer review) is the author/funder, who has granted bioRxiv a license to display the preprint in perpetuity. It is made available under a CC-BY 4.0 International license.

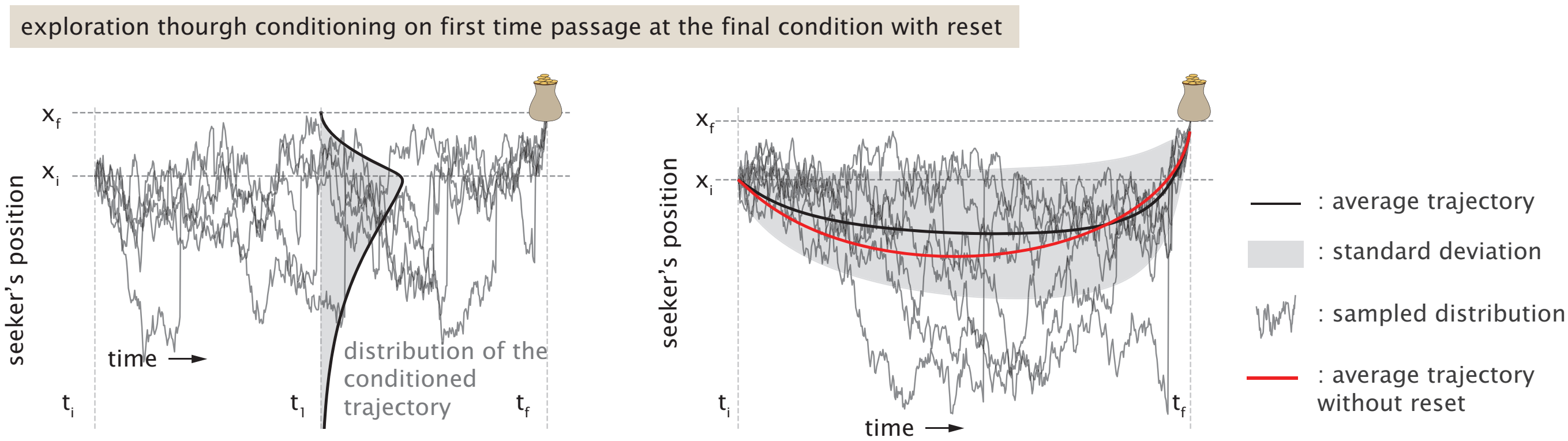

FIG. S27: Conditioned first-passage trajectories with stochastic resetting. Left: sample trajectories conditioned to first reach the target position x_T at the final time t_f. The shaded profile at the intermediate time t_1 represents the distribution of conditioned trajectories, obtained from the analytical first-passage bridge distribution with reset. Right: schematic illustration of the conditioned ensemble. The black curve denotes the mean conditioned trajectory with reset, while the shaded region indicates the corresponding standard deviation. The red curve shows the mean first-passage bridge trajectory in the absence of resetting.

The corresponding reset-modified first-passage-time density is then obtained from

$$f_{\mathrm{FP}}^{(r)}(T \mid x; x_T) = -\frac{\partial}{\partial T} S_r(T \mid x; x_T). \tag{S424}$$

This renewal equation therefore determines the first-passage statistics needed to construct the conditional density above. By analogy with the no-reset case, we can also write a local transition kernel, which is useful for simulating conditioned trajectories step by step. The one-step survival propagator in the presence of resetting is, to leading order in Δt,

$$p_{\mathrm{surv}}^{(r)}(y, t+\Delta t \mid x, t; x_T) = (1 - r\Delta t)\, p_{\mathrm{abs}}(y, t+\Delta t \mid x, t; x_T) + r\Delta t\, \delta(y), \tag{S425}$$

for $y < x_T$.

Conditioning this one-step kernel on first arrival at x_T at time t_f gives

$$p_{\mathrm{FPbb}}^{(r)}(y, t+\Delta t \mid x, t; x_T, t_f) = p_{\mathrm{surv}}^{(r)}(y, t+\Delta t \mid x, t; x_T)\, \frac{f_{\mathrm{FP}}^{(r)}(t_f - t - \Delta t \mid y; x_T)}{f_{\mathrm{FP}}^{(r)}(t_f - t \mid x; x_T)}, \tag{S426}$$

for $y < x_T$.

This local kernel gives a direct numerical prescription for generating reset-diffusion trajectories conditioned on first arrival at the target at the final time. It also lets us ask how successful trajectories are organized when unsuccessful excursions are physically terminated by resetting.

2. Decomposition into failed and successful excursions

The renewal structure of the reset process suggests a decomposition of a successful trajectory into a sequence of failed excursions terminated by resets, followed by one final successful excursion that first reaches the target. If the trajectory experiences n resets before the final first-passage event, we denote by Δ_i the duration between the $(i-1)$th and ith resets. The contribution to the first-passage-time density at t_f from trajectories with exactly n resets is

$$P_n(t_f) = \int_0^{t_f} d\Delta_1 \int_0^{t_f - \Delta_1} d\Delta_2 \cdots \int_0^{t_f - \sum_{i=1}^{n-1} \Delta_i} d\Delta_n\, g(\Delta_1, \ldots, \Delta_n). \tag{S427}$$

where

$$g(\Delta_1, \ldots, \Delta_n) = \prod_{i=1}^{n} \left[r e^{-r\Delta_i} S_0(\Delta_i) \right] e^{-r\left(t_f - \sum_{i=1}^{n} \Delta_i\right)} f_{\mathrm{FP}}\left(t_f - \sum_{i=1}^{n} \Delta_i \right) \tag{S428}$$

$$= r^n e^{-r t_f} \left[\prod_{i=1}^{n} S_0(\Delta_i) \right] f_{\mathrm{FP}} \left(t_f - \sum_{i=1}^{n} \Delta_i \right). \tag{S429}$$

bioRxiv preprint doi: https://doi.org/10.64898/2026.09.16.750450; this version posted September 17, 2026. The copyright holder for this preprint (which was not certified by peer review) is the author/funder, who has granted bioRxiv a license to display the preprint in perpetuity. It is made available under a CC-BY 4.0 International license.

Here $S_0(\Delta)$ is the no-reset survival probability and $f_{\mathrm{FP}}(s)$ is the no-reset first-passage-time density. The factor

$$re^{-r\Delta_i}S_0(\Delta_i) \tag{S430}$$

is the probability density that the ith failed excursion survives below the target for a duration Δ_i and is then terminated by a reset, while

$$e^{-r\left(t_f-\sum_{i=1}^{n}\Delta_i\right)}f_{\mathrm{FP}}\left(t_f-\sum_{i=1}^{n}\Delta_i\right) \tag{S431}$$

is the probability density that the final excursion experiences no reset and first reaches the target after the remaining time

$$s=t_f-\sum_{i=1}^{n}\Delta_i. \tag{S432}$$

The most probable number of resets n^* is obtained by maximizing $P_n(t_f)$ over integer n. For a symmetric stationary point of the joint distribution of excursion durations, the failed excursions have a common duration,

$$\Delta_1=\cdots=\Delta_{n^*}=\Delta^*, \tag{S433}$$

so that

$$t_f=n^*\Delta^*+s^*. \tag{S434}$$

The corresponding stationary durations Δ^* and s^* satisfy

$$\frac{d}{d\Delta}\left[n^*\log S_0(\Delta)+\log f_{\mathrm{FP}}(t_f-n^*\Delta)\right]=0. \tag{S435}$$

This construction identifies the dominant reset-conditioned trajectories in terms of repeated failed excursions followed by a final successful first-passage bridge. These dominant trajectories can be compared with the mean conditioned trajectories discussed above, although the most probable and mean trajectories need not coincide.

3. Mean temporal organization of reset-conditioned trajectories

The weight $P_n(t_f)$ also provides access to the statistics of the reset-conditioned ensemble. In particular, the mean number of resets is given by

$$\langle n\rangle=\frac{\sum_{n=0}^{\infty}nP_n(t_f)}{\sum_{n=0}^{\infty}P_n(t_f)}. \tag{S436}$$

For trajectories that undergo at least one reset, the mean duration of a failed excursion can be computed from

$$\langle\Delta\rangle=\frac{\sum_{n=1}^{\infty}\int d\Delta_1\cdots d\Delta_n\left(\frac{1}{n}\sum_{i=1}^{n}\Delta_i\right)g(\Delta_1,\ldots,\Delta_n)}{\sum_{n=1}^{\infty}P_n(t_f)}. \tag{S437}$$

Similarly, the mean duration of the final successful excursion is

$$\langle s\rangle=\frac{\sum_{n=0}^{\infty}\int d\Delta_1\cdots d\Delta_n\left(t_f-\sum_{i=1}^{n}\Delta_i\right)g(\Delta_1,\ldots,\Delta_n)}{\sum_{n=0}^{\infty}P_n(t_f)}. \tag{S438}$$

These averaged quantities characterize the temporal organization of reset-conditioned trajectories and can be compared with the dominant trajectory obtained from the saddle-point construction above.

For small diffusion constant, successful trajectories are dominated by a single long successful excursion, so that

$$\langle s\rangle\approx t_f,\qquad\langle n\rangle\approx 0. \tag{S439}$$

In this regime, resets are strongly suppressed because a reset leaves less time for the particle to reach the target within the prescribed final time. Failed excursions, when they occur, are therefore pushed toward short durations. As the diffusion constant increases, trajectories can accommodate more failed excursions while still reaching the target at time t_f, leading initially to an increase in the mean number of resets. At still larger diffusion constant, the behavior crosses over toward the finite large-D limit derived below.

The relative sizes of $\langle\Delta\rangle$ and $\langle s\rangle$ are not fixed across parameter space. Instead, they reflect how the fixed total duration t_f is partitioned between failed excursions and the final successful excursion. For small diffusion constant, the conditioned trajectory is dominated by the final successful excursion, so $\langle s\rangle$ is close to t_f while failed excursions are rare and short. As the diffusion constant increases, the final successful excursion can reach the target in a shorter time, leaving more of the conditioned trajectory available for reset-terminated failed excursions. In this regime, $\langle\Delta\rangle$ can become comparable to, or even larger than, $\langle s\rangle$.

bioRxiv preprint doi: https://doi.org/10.64898/2026.09.16.750450; this version posted September 17, 2026. The copyright holder for this preprint (which was not certified by peer review) is the author/funder, who has granted bioRxiv a license to display the preprint in perpetuity. It is made available under a CC-BY 4.0 International license.

4. Mean number of resets in the conditioned first-passage ensemble

The decomposition above also gives a useful way to compute the mean number of resets directly from the reset-modified first-passage-time density. Summing over all possible numbers of resets gives

$$f_{\mathrm{FP}}^{(r)}(t_f) = \sum_{n=0}^{\infty} P_n(t_f). \tag{S440}$$

Writing

$$P_n(t_f) = r^n e^{-rt_f} A_n(t_f), \tag{S441}$$

where

$$A_n(t_f) = \int d\Delta_1 \cdots d\Delta_n \left[\prod_{i=1}^{n} S_0(\Delta_i)\right] f_{\mathrm{FP}}\left(t_f - \sum_{i=1}^{n} \Delta_i\right), \tag{S442}$$

with the integrations restricted to $\sum_{i=1}^{n} \Delta_i \leq t_f$, we obtain

$$f_{\mathrm{FP}}^{(r)}(t_f) = e^{-rt_f} \sum_{n=0}^{\infty} r^n A_n(t_f). \tag{S443}$$

Since the reset rate appears as a factor r^n for trajectories containing n reset events, differentiation with respect to r gives

$$\langle n \rangle = r \frac{\partial}{\partial r} \log\left[e^{rt_f} f_{\mathrm{FP}}^{(r)}(t_f)\right], \tag{S444}$$

or equivalently

$$\langle n \rangle = r \frac{\partial}{\partial r} \log f_{\mathrm{FP}}^{(r)}(t_f) + rt_f. \tag{S445}$$

This expression relates the mean number of resets in the conditioned ensemble directly to the sensitivity of the first-passage-time density to the reset rate.

The limiting behavior can be understood directly from the reset-sector weights. In the small-D limit, a trajectory that reaches the target at the prescribed time t_f is strongly penalized if a finite portion of the available time is spent in failed excursions. Indeed, writing

$$s = t_f - \sum_{i=1}^{n} \Delta_i, \tag{S446}$$

the ratio of no-reset first-passage densities is

$$\frac{f_{\mathrm{FP}}(s)}{f_{\mathrm{FP}}(t_f)} = \left(\frac{t_f}{s}\right)^{3/2} \exp\left[-\frac{L^2}{4D}\left(\frac{1}{s} - \frac{1}{t_f}\right)\right], \qquad L = x_T - x_i. \tag{S447}$$

As $D \to 0$, the dominant reset-containing histories therefore have a total failed-excursion time that shrinks to zero. For fixed n this gives

$$\frac{P_n(t_f)}{P_0(t_f)} \sim \left(\frac{4rDt_f^2}{L^2}\right)^n, \tag{S448}$$

and hence

$$\langle n \rangle \to 0 \qquad (D \to 0). \tag{S449}$$

The large-D limit is different. Although each no-reset survival factor becomes small,

$$S_0(\Delta) \sim \frac{L}{\sqrt{\pi D \Delta}}, \tag{S450}$$

bioRxiv preprint doi: https://doi.org/10.64898/2026.09.16.750450; this version posted September 17, 2026. The copyright holder for this preprint (which was not certified by peer review) is the author/funder, who has granted bioRxiv a license to display the preprint in perpetuity. It is made available under a CC-BY 4.0 International license.

the one-reset contribution remains of the same order in D as the zero-reset contribution. In particular,

$$P_0(t_f) \sim e^{-rt_f} \frac{L}{2\sqrt{\pi D} t_f^{3/2}}, \tag{S451}$$

while

$$P_1(t_f) \sim e^{-rt_f} r \frac{L}{\sqrt{\pi D t_f}}. \tag{S452}$$

Therefore,

$$\frac{P_1(t_f)}{P_0(t_f)} \to 2rt_f. \tag{S453}$$

All sectors with $n \geq 2$ are suppressed relative to these leading contributions, so that

$$\langle n \rangle \to \frac{2rt_f}{1+2rt_f} \qquad (D \to \infty). \tag{S454}$$

Thus the mean number of resets vanishes in the small-diffusion limit but approaches a finite value in the large-diffusion limit.

bioRxiv preprint doi: https://doi.org/10.64898/2026.09.16.750450; this version posted September 17, 2026. The copyright holder for this preprint (which was not certified by peer review) is the author/funder, who has granted bioRxiv a license to display the preprint in perpetuity. It is made available under a CC-BY 4.0 International license.

[1] Milton Abramowitz, Irene A Stegun, and Jacques E Romain. Handbook of mathematical functions, with formulas, graphs, and mathematical tables, 1966. Chapter 13: Confluent Hypergeometric Functions.

[2] Johannes Bäumler. Recurrence and transience of symmetric random walks with long-range jumps. *Electron. J. Probab.*, 28:1–24, 2023.

[3] Benjamin Besga, Alfred Bovon, Artyom Petrosyan, Satya N. Majumdar, and Sergio Ciliberto. Optimal mean first-passage time for a Brownian searcher subjected to resetting: Experimental and theoretical results. *Phys. Rev. Res.*, 2(3):032029(R), 2020.

[4] Uttam Bhat, Caterina De Bacco, and S Redner. Stochastic search with Poisson and deterministic resetting. *J. Stat. Mech.: Theory Exp.*, 2016(8):083401, 2016.

[5] David Blackwell. On an equation of wald. *The Annals of Mathematical Statistics*, 17(1):84–87, 1946.

[6] K.L. Chung. Appendix 1: Pòlya's work in probability. In Gerald L. Alexanderson, editor, *The Random Walks of George Pòlya*, pages 193–200. Mathematical Association of America, Washington, DC, 2000.

[7] Benjamin De Bruyne, Satya N Majumdar, and Grégory Schehr. Optimal resetting Brownian bridges via enhanced fluctuations. *Phys. Rev. Lett.*, 128(20):200603, 2022.

[8] Peter G Doyle and J Laurie Snell. *Random walks and electric networks*, volume 22. American Mathematical Soc., 1984.

[9] Rick Durrett. *Probability: Theory and Examples*, volume 49. Cambridge University Press, 5 edition, 2019.

[10] Martin R Evans, Satya N Majumdar, and Kirone Mallick. Optimal diffusive search: nonequilibrium resetting versus equilibrium dynamics. *J. Phys. A: Math. Theor.*, 46(18):185001, 2013.

[11] Alexander K Hartmann and Satya N Majumdar. Diffusion with stochastic resetting on a lattice. *Phys. Rev. E*, 112(3):034102, 2025.

[12] T. E. Holy and S. Leibler. Dynamic instability of microtubules as an efficient way to search in space. *Proc. Natl. Acad. Sci. U.S.A.*, 91(12):5682–5685, 1994.

[13] J. Kondev, M. Kirschner, H. G. Garcia, G. L. Salmon, and R. Phillips. Biological processes as exploratory dynamics. *Biophys. J.*, 125(12):2812–2837, 2025.

[14] Thomas Koshy. *Catalan numbers with applications*. Oxford University Press, 2008.

[15] Manjunath Krishnapur and Yuval Peres. Recurrent graphs where two independent random walks collide finitely often. *Electron. Commun. Probab.*, 9:72–81, 2004.

[16] Steven P. Lalley. One-Dimensional Random Walks. Lecture notes for Statistics 312: Stochastic Processes, University of Chicago, Autumn 2016, 2016. Available at `https://galton.uchicago.edu/~lalley/Courses/312/RW.pdf`; accessed 2026-06-17.

[17] Gregory F Lawler and Vlada Limic. *Random walk: a modern introduction*, volume 123. Cambridge University Press, 2010.

[18] Jack Murtagh. Socially awkward math. *Sci. Am.*, 334(3):72, 2026. Published online February 17, 2026.

[19] Edward W. Ng and Murray Geller. A table of integrals of the error functions. *Journal of Research of the National Bureau of Standards, Section B: Mathematical Sciences*, page 1, 1969.

[20] Arnab Pal, Rakesh Chatterjee, Shlomi Reuveni, and Anupam Kundu. Local time of diffusion with stochastic resetting. *J. Phys. A: Math. Theor.*, 52(26):264002, 2019.

[21] Georg Pólya. Über eine aufgabe der wahrscheinlichkeitsrechnung betreffend die irrfahrt im straßennetz. *Math. Ann.*, 84(1):149–160, 1921.

[22] George Pólya. *George Pólya: Collected Papers: Probability, Combinatorics, Teaching and Learning in Mathematics*, volume 4 of *Mathematicians of Our Time*. The MIT Press, sep 1984.

[23] Richard P Stanley. *Catalan numbers*. Cambridge University Press, 2015.

[24] Eric W. Weisstein. Pólya's Random Walk Constants. `https://mathworld.wolfram.com/PolyasRandomWalkConstants.html`, 2026. From MathWorld—A Wolfram Resource. Accessed 17 June 2026.
